# GATA2 Promotes Hematopoietic Development and Represses Cardiac Differentiation of Human Mesoderm

**DOI:** 10.1016/j.stemcr.2019.07.009

**Published:** 2019-08-08

**Authors:** Julio Castaño, Sergi Aranda, Clara Bueno, Fernando J. Calero-Nieto, Eva Mejia-Ramirez, Jose Luis Mosquera, Enrique Blanco, Xiaonan Wang, Cristina Prieto, Lorea Zabaleta, Elisabetta Mereu, Meritxell Rovira, Senda Jiménez-Delgado, Daniel R. Matson, Holger Heyn, Emery H. Bresnick, Berthold Göttgens, Luciano Di Croce, Pablo Menendez, Angel Raya, Alessandra Giorgetti

**Affiliations:** 1 Center of Regenerative Medicine in Barcelona (CMRB), Hospital Duran i Reynals, Gran Via de L'Hospitalet, 199-203, Hospitalet de Llobregat, Barcelona 08908, Spain; 2 Center for Networked Biomedical Research on Bioengineering, Biomaterials, and Nanomedicine (CIBER-BBN), Madrid 28029, Spain; 3 Center for Genomic Regulation (CRG), The Barcelona Institute of Science and Technology, Universitat Pompeu Fabra, Barcelona 08003, Spain; 4 Josep Carreras Leukemia Research Institute and Department of Biomedicine, School of Medicine, University of Barcelona, Barcelona 08036, Spain; 5 Department of Hematology, Wellcome and MRC Cambridge Stem Cell Institute and Cambridge Institute for Medical Research, University of Cambridge, Cambridge, UK; 6 Bioinformatics Unit, Bellvitge Biomedical Research Institute (IDIBELL), Barcelona, 08908 Spain; 7 Laboratory of Hematological Diseases, Fundación Inbiomed, San Sebastian, 20009, Spain; 8 CNAG-CRG, Center for Genomic Regulation (CRG), Barcelona Institute of Science and Technology (BIST), Barcelona, Spain; 9 Department of Cell and Regenerative Biology, UW-Madison Blood Research Program, Carbone Cancer Center, University of Wisconsin School of Medicine and Public Health, Madison, WI 53705, USA; 10 Universitat Pompeu Fabra, Barcelona, Spain; 11 Institució Catalana de Recerca i Estudis Avançats (ICREA), Barcelona 08010, Spain; 12 Centro de Investigación Biomedica en Red en Cancer (CIBERONIC) ISCIII, Barcelona, Spain

**Keywords:** GATA2, human iPSCs, hemogenic specification, hematopoiesis, mesoderm diversification, cardiac development

## Abstract

In vertebrates, GATA2 is a master regulator of hematopoiesis and is expressed throughout embryo development and in adult life. Although the essential role of GATA2 in mouse hematopoiesis is well established, its involvement during early human hematopoietic development is not clear. By combining time-controlled overexpression of *GATA2* with genetic knockout experiments, we found that GATA2, at the mesoderm specification stage, promotes the generation of hemogenic endothelial progenitors and their further differentiation to hematopoietic progenitor cells, and negatively regulates cardiac differentiation. Surprisingly, genome-wide transcriptional and chromatin immunoprecipitation analysis showed that GATA2 bound to regulatory regions, and repressed the expression of cardiac development-related genes. Moreover, genes important for hematopoietic differentiation were upregulated by GATA2 in a mostly indirect manner. Collectively, our data reveal a hitherto unrecognized role of GATA2 as a repressor of cardiac fates, and highlight the importance of coordinating the specification and repression of alternative cell fates.

## Highlights

• GATA2 promotes hemogenic emergence during human iPSC differentiation
• GATA2 enhances hematopoietic differentiation of human iPSCs
• GATA2 acts as a direct repressor of cardiac fates during mesoderm specification

## Graphical Abstract

**Figure:**
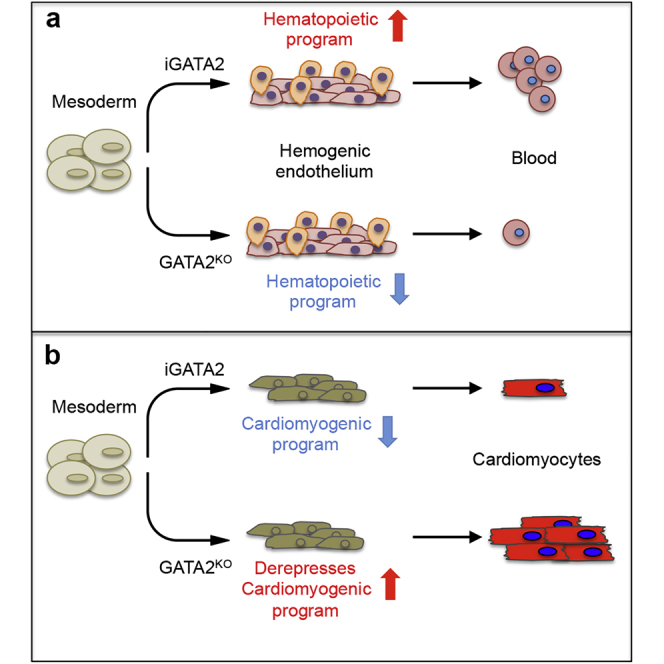

## Teaser

Giorgetti A. and colleagues demonstrate that GATA2 induction in early mesodermal cells leads to the robust generation of hemogenic endothelial and hematopoietic cells from human iPSCs. They show the ability of GATA2 to instruct mesodermal precursors toward a hematopoietic cell fate and concurrently inhibit cardiac fates. This study expands our understanding of the regulatory networks that control early human hematopoiesis.

## Introduction

During embryonic development, hematopoietic stem cells (HSCs) emerge from hemogenic endothelium in the ventral wall of the aorta-gonad-mesonephros (AGM) region (Dzierzak and Speck, 2008, Ivanovs et al., 2011, Ivanovs et al., 2014), and their specification is tightly orchestrated by temporal changes in the expression of master regulators during endothelial-to-hematopoietic transition (EHT). While animal models have been crucial in identifying several master regulators, such as GATA2, RUNX1, and TAL1 (Robert-Moreno et al., 2005, Wilson et al., 2010a, Wilson et al., 2010b), how these factors drive human HSC emergence during EHT remains poorly understood.

GATA2 belongs to an evolutionarily conserved family of zinc finger transcription factors comprising six members: GATA1 to GATA6 (Merika and Orkin, 1993, Molkentin, 2000). GATA2, together with GATA1 and GATA3, are categorized as “hematopoietic” GATA factors and regulate the development of diverse hematopoietic lineages (Bresnick et al., 2012, Johnson et al., 2012, Katsumura et al., 2017, Orkin, 1992). The importance of GATA2 in HSC specification was first highlighted by gene targeting studies, because ablation of *Gata2* is embryonic lethal at embryonic day (E)10.5 due to the collapse of primitive and definitive hematopoiesis (Gao et al., 2013, Ling et al., 2004, Tsai and Orkin, 1997). Notably, analysis of chimeric embryos generated with *Gata2*-null embryonic stem cells (ESCs) indicated that these cells failed to contribute to any hematopoietic lineage (Tsai et al., 1994). Likewise, mouse *Gata2*-null endothelial cells failed to produce HSCs because of impaired EHT (de Pater et al., 2013, Gao et al., 2013, Johnson et al., 2012, Lim et al., 2012). A primary role of GATA2 in promoting EHT has been recently demonstrated in humans (Gomes et al., 2018, Kang et al., 2018, Zhou et al., 2019).

In adult hematopoiesis, GATA2 is expressed at high levels in HSCs, early hematopoietic progenitors, and in erythroid/megakaryocyte lineages (Vicente et al., 2012). Recent studies showed that *GATA2* haploinsufficiency is associated with some familial cases of myelodysplastic syndrome, bone marrow failure, immunodeficiency, and MonoMAc syndrome (Dickinson et al., 2011, Hahn et al., 2011, Wlodarski et al., 2016), further supporting its important role in HSCs. Conversely, enforced expression of *GATA2* in cord blood-derived HSCs confers increased quiescence, an important hallmark of HSCs (Tipping et al., 2009).

We sought to explore the role of GATA2 during human hematopoietic development by inducing *GATA2* expression in differentiating human induced pluripotent stem cells (hiPSCs) (Takahashi et al., 2007). We show that *GATA2* induction during mesoderm patterning robustly promotes the generation of hemogenic endothelial progenitors (HEPs), and their further differentiation into hematopoietic progenitor cells (HPCs). Global transcriptome analysis and chromatin immunoprecipitation sequencing (ChIP-seq) combined with DNA massive sequencing revealed that GATA2 directly represses genes that promote cardiac cell fate differentiation and activates master hematopoietic regulators via direct and indirect mechanisms. Remarkably, *GATA2* knockout impaired hematopoietic development and enhanced cardiac potential of mesodermal progenitors.

## Results

### GATA2 Promotes Robust Hematopoietic Differentiation

**Figure 1:**
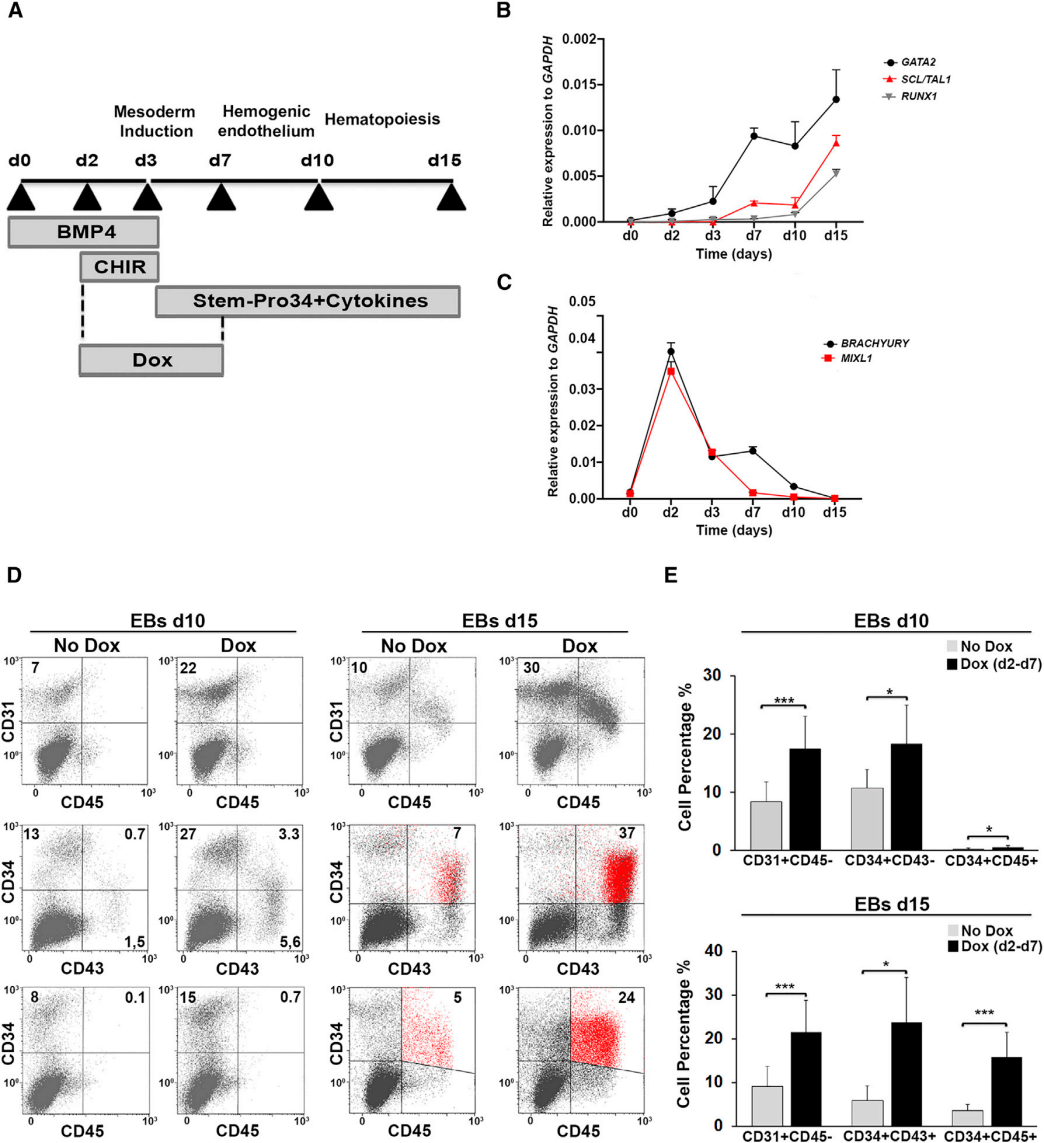
Early GATA2 Induction Enhances Hematopoietic Development from hiPSCs (A) hiPSC hematopoietic differentiation based on EB generation. (B) Time course of endogenous *GATA2*, *SCL/TAL1*, and *RUNX1* expression during EB development, normalized to *GAPDH*. (C) Time course of endogenous mesodermal marker expression (*BRACHYURY* and *MIXL1*) during EB development, normalized to *GAPDH*. In (B) and (C), data represent the mean ± SD of three independent experiments. (D) Representative flow cytometry analysis of HEPs (CD31^+^CD34^+^CD45^–^ and CD34^+^CD43^–^CD45^–^) and HPCs (CD34^+^CD45^+^ and CD43^+^CD45^+^) in EBs at days 10 and 15 in control and Dox-treated cells. (E) Quantitative summary of HEP and HPC analysis at days 10 and 15 of EB differentiation in control and Dox-treated cells. Data represent the mean ± SD of 10 independent experiments. ^∗^p < 0.05, ^∗∗∗^p < 0.001.

To analyze the impact of GATA2 in early human hematopoiesis, we first examined endogenous GATA2 expression in hiPSCs induced to form embryoid bodies (EBs) in serum-free medium with the successive addition of BMP4 (days 0–3), CHIR92001 (days 2–3), and hematopoietic cytokines (days 3–15) (Figure 1A). This protocol promotes mesoderm induction (days 2–3), specification of mesodermal cells to bipotential hemato-endothelial progenitors (CD31^+^CD34^+^CD43^-^CD45^−^; days 3–10) that can originate both endothelial and hematopoietic cells and could be considered equivalent to HEPs (Ayllon et al., 2015), and further commitment of HEPs to definitive HPCs (CD34^+^CD43^+^CD45^+^; days 10–15) (Giorgetti et al., 2017, Sturgeon et al., 2014). *GATA2* was initially expressed at day 2 (Figure 1B), at the onset of mesoderm formation marked by the expression of *T* and *MIXL1* (Figure 1C). Its expression then progressively increased along with the emergence of HEPs and HPCs, in parallel with the master hemogenic regulators *RUNX1* and *SCL* (Figure 1B).

We next established transgenic hiPSCs in which the expression of transgenic *GATA2* could be temporally controlled by doxycycline (Dox) administration (hereafter termed iGATA2-hiPSCs) (Figure S1A). Robust transgenic overexpression of *GATA2* was confirmed in four clones (CL6, CL9, CL201, CL204) derived from two independent iGATA2-hiPSC lines by western blotting after 2 days of Dox treatment (Figure S1B). qRT-PCR analysis and *in vivo* functional assays showed that iGATA2-hiPSCs retained the expression of pluripotency markers and also the capacity to generate teratomas (Figure S1C).

Then, considering the expression of endogenous *GATA2*, we induced *GATA2* expression from day 2 to 7 in EBs generated from iGATA2-hiPSCs (Figures 1A and S1D–S1G). Flow cytometry analysis showed that enforced expression of *GATA2* significantly enhanced the production of HEPs (∼2.5-fold increase of CD31^+^CD34^+^CD45^−^ cells and ∼2-fold increase of CD34^+^CD43^–^CD45^–^ cells) in EBs at day 10 (Figures 1D and 1E), and promoted the generation of HPCs (∼5-fold increase of CD34^+^CD43^+^CD45^+^ cells) at day 15 (Figures 1D and 1E).

**Figure 2:**
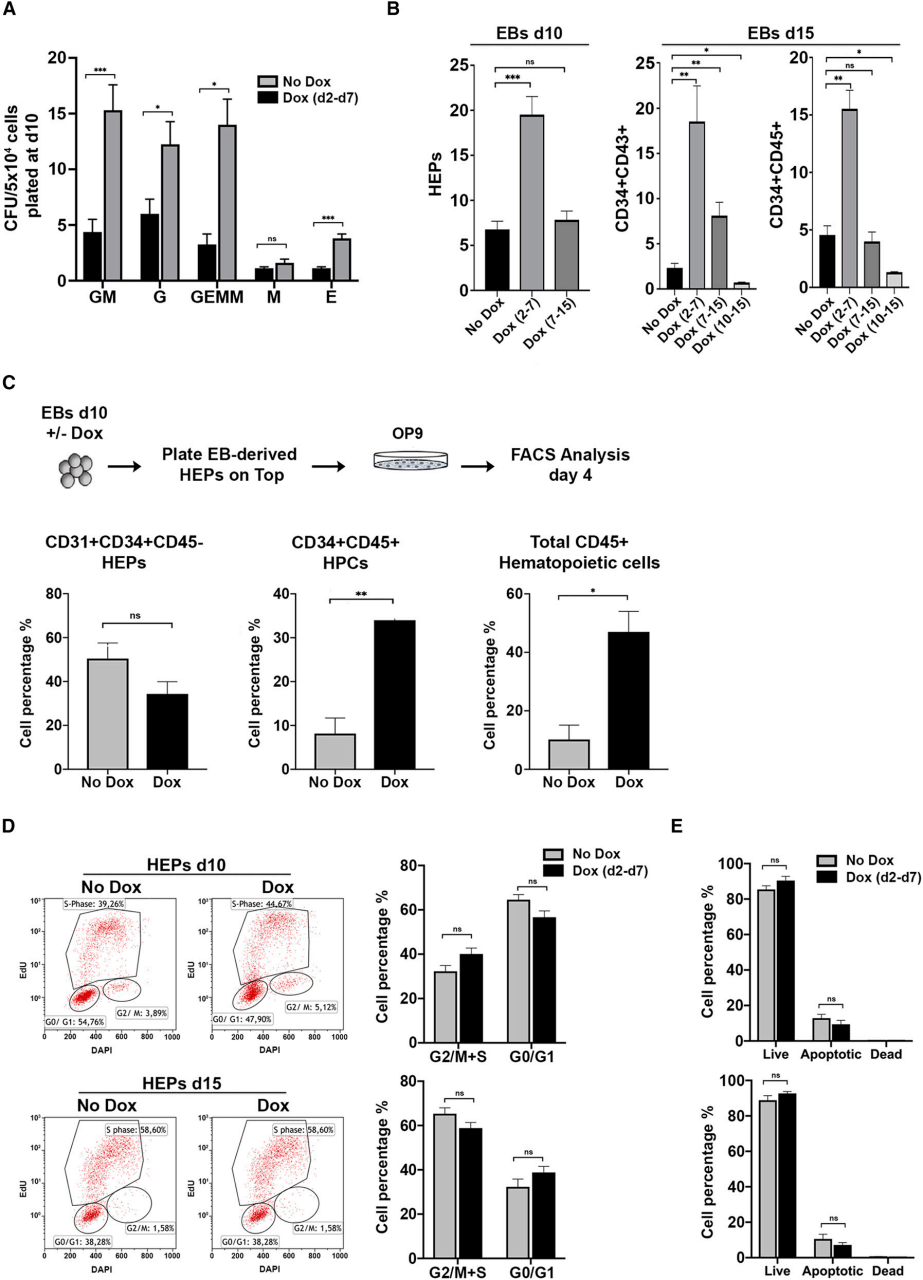
GATA2 Induction Promotes Hemogenic Endothelium Transition (A) CFU potential of day 10 EB progenitors in control and Dox-treated cells. Colonies were counted from each group after 2 weeks of culture and scored for the following morphological subsets: burst-forming unit-erythroid (E); CFU-granulocyte, macrophage (GM); CFU-granulocyte, erythroid, macrophage, megakaryocyte (GEMM); CFU-granulocyte (CFU-G); and CFU-macrophage (CFU-M). Data represent the mean ± SD of the total number of colonies per 50,000 cells seeded of 6 independent experiments. (B) Quantitative summary of HEP and HPC analysis at days 10 and 15 of EB differentiation following stepwise treatment with Dox. Data represent the mean ± SD of 6 independent experiments. (C) Schematic of sorted HEP cell differentiation using OP9 coculture (upper panel). GATA2-induced HEPs showed a higher capacity to differentiate into CD34^+^/CD45^+^ and CD45^+^ hematopoietic cells (lower panel). Data represent the mean ± SD of 3 independent experiments. (D) Representative dot plots and bar graphs show cell-cycle analysis using EdU and DAPI staining on HEPs and HPCs treated or not with Dox. Data represent the mean ± SD of 3 independent experiments. (E) Apoptosis analysis in HEPs and HPCs in control and Dox-treated cells. Data represent the mean ± SD of 3 independent experiments. ^∗^p < 0.05, ^∗∗^p < 0.01, ^∗∗∗^p < 0.001.

We used colony-forming unit (CFU) assays to confirm that GATA2 overexpression promotes hematopoiesis from iGATA2-hiPSCs. Dox treatment (days 2–7) significantly increased the total number of hematopoietic CFCs in day 10 EBs (Figure 2A). Notably, CFU scoring revealed an enhancement in all types of hematopoietic colonies (Figure 2A), suggesting that GATA2 expression promotes hematopoietic commitment by inducing mesodermal specification to HEPs at very early stages.

To better understand the role of GATA2 in early hematopoiesis, we treated iGATA2-hiPSCs with Dox at distinct stages of EB development (days 2–7, 7–15, and 10–15). Induction of GATA2 at days 2–7 had the greatest effect on HEP and HPC generation (Figure 2B), while treatment at days 7–15 had no significant effects on the HEP population, and only a small effect was observed for early HPCs. By contrast, GATA2 overexpression at days 10–15 led to a significant decrease in HPC numbers (Figure 2B), consistent with previous findings showing that high GATA2 expression in HSCs blocks normal hematopoiesis (Persons et al., 1999, Tipping et al., 2009).

To address whether the higher hematopoietic output could be a consequence of a higher HEP generation, CD34^+^ cells were purified from day 10 EBs with or without Dox treatment, and were cultured on OP9 stromal cells (Choi et al., 2009, Ramos-Mejia et al., 2014) (Figure 2C, upper panel). After 4 days of coculture, both CD34^+^CD45^+^ and total CD45^+^ cell subpopulations increased significantly in the cocultures derived from Dox-treated cells (∼3- and 4-fold, respectively), whereas the number of HEPs decreased slightly (Figure 2C, lower panel). To further characterize the effect of GATA2 during mesoderm patterning, we performed single-cell cloning assays of mesodermal cells (KDR^+^CD34^–^CD31^–^) using OP9 stromal cells and conditions that support hemato-endothelial differentiation. Dox treatment slightly increased the hematopoietic/endothelial ratio over control (no-Dox) (Figure S2A, upper table). We repeated single-cell clonal analysis of CD31^+^CD34^+^CD43^–^ cells purified from day 7 EBs with or without Dox, finding that Dox-treated cells showed an increased number of hematopoietic colonies but a smaller proportion of endothelial growth (Figure S2A, lower table), overall increasing the hematopoietic/endothelial ratio by >4-fold.

Because GATA2 has been associated with adult HSC survival (Tipping et al., 2009), we questioned whether the increase in hematopoiesis was the consequence of GATA2-mediated proliferation/survival of emerging HEPs and HPCs. We analyzed apoptosis and cell-cycle distribution after GATA2 induction in HEPs and HPCs at days 10 and 15 of EB development, respectively, finding that GATA2 induction did not affect survival or proliferation of differentiating cells (Figures 2D, 2E, and S2B).

Overall, our data are consistent with recent reports (Kang et al., 2018, Zhou et al., 2019) showing that, rather than induction of HEP specification or the selective proliferation/survival of HEPs/HPCs, GATA2 induces hematopoietic development by promoting EHT.

### GATA2 Activates the Hematopoietic Program and Inhibits Cardiac Genes

To gain mechanistic insight into how GATA2 promotes hematopoietic development, we performed RNA sequencing (RNA-seq) of fluorescence-activated cell sorting (FACS)-sorted control or GATA2-overexpressing HEPs from day 2 to 7 of differentiation. We used the criteria of >1.5-fold change and adjusted p value < 0.05 to identify differentially expressed genes (DEGs) in the two treatment groups.

Among 1,127 genes significantly deregulated by GATA2 induction, 700 were downregulated and 427 were upregulated (Table S2). Consistent with the *in vitro* results, GATA2 activated a broad spectrum of genes regulating HSC/HPC development (e.g., *RUNX1*, *MYB*, *STAT1*, *ITGA2B/CD41*, *SPN/CD43*, *SPI1/PU.1*, *ZBT3*, and *ALDH1A1*), as well as of genes of myeloid (*CD33*, *CD53*, *CD48*, *CSFR1*, and *MPO*) and erythroid (*NFE2*, *GATA1*, *KLF1*, *HBZ*, *HBE1*, *HBA1*, and *HBG2*) lineages (Figure S3A). Proinflammatory cytokines have been proposed as positive regulators of definitive hematopoiesis in the mouse AGM region and its zebrafish equivalent (Espin-Palazon et al., 2014, He et al., 2015, Sawamiphak et al., 2014). In accord with this, gene ontology (GO) and gene set enrichment analysis showed that GATA2-overexpressing HEPs were highly enriched for genes associated with immune response (*IRF7*, *IFI27*, *IFIT1*, *TMEM173*, *IFI6*, *IFITM1*, *TRIM6*, *TRIM14*, and *TRIM25*) (Figure S3A). Surprisingly, several highly significant GO categories of downregulated genes were related to heart development and cardiogenesis (Figure S3B), including transcription factors such as *TBX3*, *MYOCD*, *PTX2*, *NR2F2*, and *FOXC2*, and structural genes including *TNNC1*, *RYR3*, *SPNS2*, *DVL3*, *SMO*, *NEBL*, *HEG1*, and *CCM2L* (Figure S3B).

Furthermore, expression of genes related to angiogenesis and endothelial cell differentiation (*JAG-1*, *KDR*, *SOX17*, *PCDH12*, *TEK*, *ESM1*, and *SCUBE1*) were also found to be downregulated (Figure S3B). These data strongly suggest that GATA2 has a dual activity during mesodermal patterning.

### GATA2 Directly Binds Cardiac Genes

**Figure 3:**
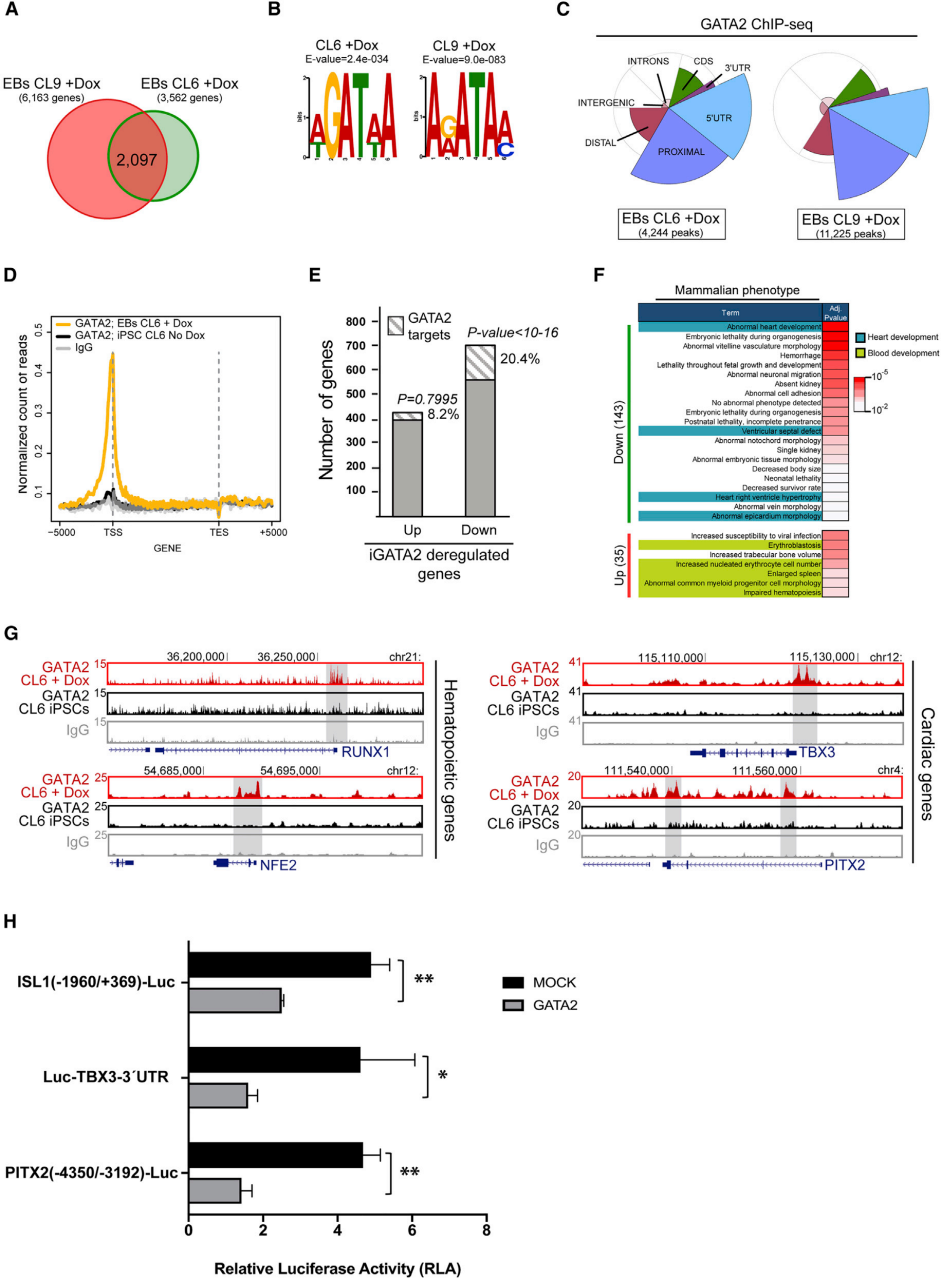
Identification of GATA2 Target Genes during Mesoderm Specification (A) Venn diagram indicating the overlap between the number of genes found targeted by GATA2 in each replicate of the ChIP-seq of GATA2 in EBs (plus Dox). (B) MEME-ChIP motif analysis on the sequence of the GATA2 peaks in two independent ChIP-seq replicates showing an enrichment of GATA2 motifs at the center of the ChIP-seq peaks, as the top-ranked motif. (C) Genomic distribution of ChIP-seq peaks of GATA2 compared with the whole genome in two independent clones. The pie chart represents the distribution of GATA2 peaks corrected by the genome-wide distribution of each gene feature (background circle distribution). The pie chart indicates that GATA2 preferentially occupies TSS neighborhood regions, including 5′ UTR and PROXIMAL regions. The DISTAL region is the region within 2.5 and 0.5 kbp upstream of the TSS. The PROXIMAL region is the region within 0.5 kbp of the TSS. CDS is the protein CoDing Sequence. INTRONS are intronic regions. INTERGENIC is the remainder of the genome. TSS is the transcription start site. (D) Meta-gene plot showing the GATA2 ChIP-seq profile occupancy in iPSCs (CL6 no-Dox) and EBs (CL6 plus Dox) and IgG from −5 kb of the TSS until +5 kb of transcription end site (TES). (E) Histogram of the proportion of GATA2 target genes deregulated after induction of GATA (iGATA2) (up or down, 1.5-fold change, false discovery rate < 0.05) during EB development (p value was calculated using chi-square test). (F) Functional analysis on the mammalian phenotype enrichment ontology for the deregulated GATA2 target genes (MGI database). (G) UCSC genome browser screenshots of several developmental hematopoietic and cardiac genes targeted by GATA2. (H) Relative luciferase activity of COS7 cells transfected with reporter plasmids for *ISL1* (−1,960/+369), *PITX2* (−4,350/−3,192), or *TBX3* 3′ UTR genes and pWPI-GATA2 plasmid. Relative luciferase activity is shown as the ratio of luciferase activity to that in cells cotransfected with mock vector. A representative result of three independent experiments performed in triplicate is shown as the mean ± SD. Differences were determined using Student's t test; ^∗^p < 0.05, ^∗∗^p < 0.01.

To determine the direct effects of GATA2 on gene transcription, we performed ChIP-seq analysis for GATA2 occupancy on FACS-sorted GFP^+^ cells at day 7 of EB development, coinciding with the maximum expression of the GATA2 transgene (Figure S1E). We identified 2,097 GATA2 binding-associated genes (Figures 3A, S3C, and S3D). GATA2 was found to be enriched around the transcriptional start site of many genes harboring a GATA2 binding motif (Figures 3B–3D and S3E), indicating that GATA2 does not occupy unscheduled genomic binding sites in iGATA2-hiPSCs. Integrated analysis with RNA-seq data indicated that only 8% of upregulated genes were decorated with GATA2 (35 genes in total), whereas up to 20% of downregulated genes were occupied by GATA2 (143) (Figure 3E). Probabilistic analysis indicated that GATA2 occupancy on repressed genes was significantly higher than would be expected by random chance (p < 10^−16^), suggesting a predominant function of GATA2 repressing gene transcription during mesodermal lineage differentiation (Figure 3E). Among the upregulated GATA2 target genes, we identified determinants for hematopoietic development such as *RUNX1* and *NFE2* (Figures 3F and 3G). Of note, several of the downregulated GATA2 target genes were associated with heart development, such as *TBX3* and *PITX2* (Figures 3F and 3G), confirming the RNA-seq data.

The specific binding of GATA2 to proximal gene regions of cardiac regulators strongly suggests that GATA2 directly controls the repression of genes involved in cardiac development. To test this, we used a luciferase reporter assay in COS7 cells for three cardiac genes (*TBX3*, *PITX2*, and *ISL-1*), according to the ChIP-seq data. Luciferase activity driven by the 3′ UTR of the *TBX3* promoter, the −1,960/+369 region of the *ISL-1* promoter, and the −4,350/−3,192 region of the *PITX2* promoter, was significantly reduced by GATA2 coexpression (Figure 3H). Thus, in addition to validating GATA2 as a pro-hematopoietic fate regulator, these data point to an additional and undescribed role for GATA2 in repressing cardiac regulation.

### Single-Cell RNA-Seq of Mesodermal and Hemato-Endothelial Progenitors

To better characterize the effect of GATA2 overexpression on mesodermal diversification, we performed single-cell RNA-seq (scRNA-seq) on FACS-purified mesodermal (KDR^+^CD34^–^CD31^–^; EBs d5) and hemato-endothelial progenitor (KDR^+^CD34^+^CD43^–^; EBs d7) cells with or without Dox (Figure S4A). EB-derived cells from day 2 were profiled as the starting population.

**Figure 4:**
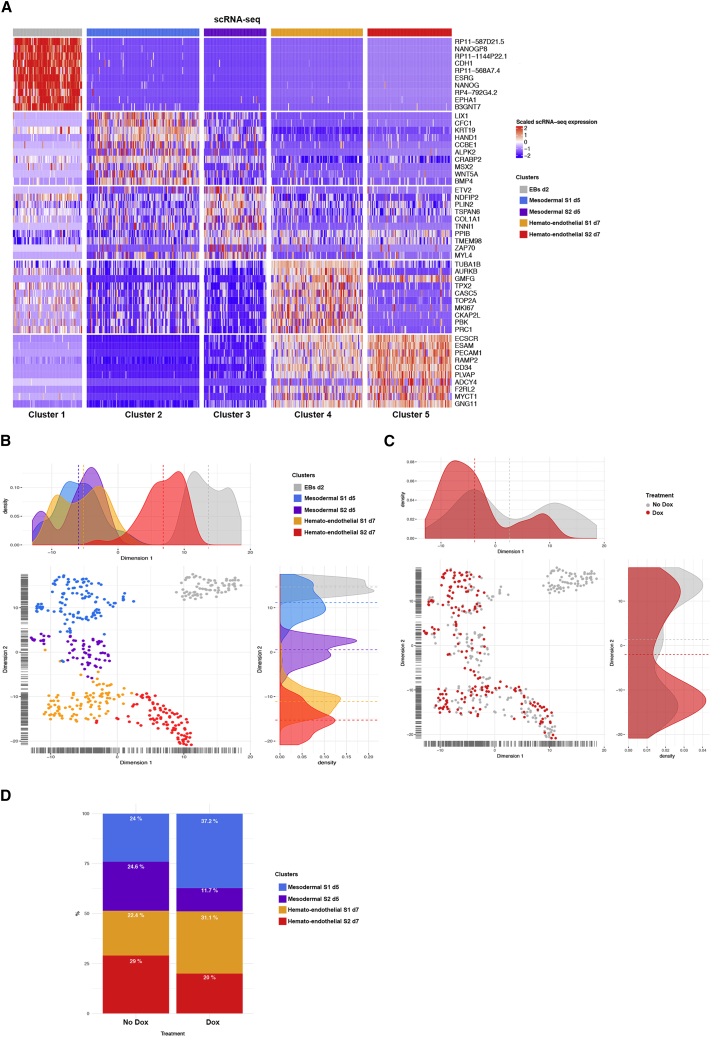
Single-Cell RNA-Seq Analysis Reveals GATA2 as a Driver of Hemato-Endothelial Specification (A) Heatmap showing the expression pattern of top 10 gene markers distinguishing 5 clusters from scRNA-seq analysis. Colored top bar indicates assigned cluster. Red indicates the highest scaled expression and blue the lowest. (B) t-SNE visualization plot of day 2 (d2) EBs, mesodermal d5 and hemato-endothelial d7 cells according to clusters in (A). (C) t-SNE visualization plot of single cells of each cluster retrospectively colored by Dox (red) and no-Dox treatment (gray). (D) Stacked bar diagram showing the percentage of cells in each cluster with respect to treatment conditions.

In unsupervised clustering based on the Seurat method (Butler et al., 2018), we identified five transcriptionally distinct cell clusters (Figure 4A). The t-distributed stochastic neighbor embedding (t-SNE) projection visualized that day 2 EB-derived cells formed one expression signature cluster, whereas mesodermal (day 5) and hemato-endothelial (day 7) cells showed the presence of two subpopulations in each cluster (Figure 4B). We assigned biological identities to each cluster based on the expression of key marker genes (adjusted p value < 0.05) (Table S3). Cluster 1 (EBs day 2) showed high expression of pluripotency genes (*NANOG*, *OCT4/POU5f1*, *ZFP42/REX1*, *DPPA4*, and *SALL2*), indicating maintenance of undifferentiated pluripotent cells. Cluster 2 (mesodermal S1) showed a multi-lineage mesodermal identity (*BMP4*, *MSX2*, *PDGFRA*, *LGR5*, *CDX2*, *FRZB*, *HOXA1*, *BMP5*, *FTH1*, *WNT5A*, *WNT5B*, *HAND1*, *TNNT1*, and *TBX3*). Cluster 3 (mesodermal S2) lacked expression of mesodermal markers, but co-expressed *ETV2*, its target SCL/*TAL1*, and *EGFL7*, which are responsible for restricting mesoderm specification to endothelium fate (Wareing et al., 2012), and genes suggestive of a mesenchymal phenotype (*COL1A1*, *COL6A3*, *COL3A1*, *COL6A2*, *ACTA2*, and *LUM*). Cluster 4 (hemato-endothelial S1) and cluster 5 (hemato-endothelial S2) shared the expression of several key hemato-endothelial markers (*CDH5*, *PECAM1*, *ICAM2*, *CD40*, *ESAM*, *FLI1*, *ERG*, *ETS1*, and *HHEX*); however, hemato-endothelial S2 cells showed a more restrictive endothelial identity (expressing selectively *NOTCH4*, *SOX7*, *NRP2*, *TEK*, and *NOS3*). Also, we noted that hemato-endothelial S1 was enriched for genes that regulate cell cycle (*AURKB*, *TOP2A*, *CDK1*, *MKI67*, *BRCA2*, *CASC5*, and *CDCA5*).

Next, we retrospectively colored each cluster on the basis of Dox and no-Dox conditions (Figure 4C). For each cluster, the number of cells per condition was calculated and each Dox condition was compared with that of the corresponding no-Dox condition (Table S3). A chi-square test showed that treatment and cluster populations were not independent (p = 0.0002687). Considering an adjusted p value < 0.05, the *post hoc* comparisons showed that, at day 5 of EB development, the proportion of mesodermal S1 cells increased significantly in Dox conditions (chi-square = 6.816191, adjusted p value = 0.018067005) (Figure 4D). At day 7, GATA2 overexpression induced an enrichment of the hemato-endothelial S1 cluster (chi-square = 3.082502, adjusted p value = 0.079138704) (Figure 4D).

Based on these data, we speculate that GATA2 enhances the proportion of cells with a multi-lineage mesodermal signature and further increases the probability, at the single-cell level, that a mesodermal progenitor acquired a hemato-endothelial transcriptional profile. These data are consistent with our FACS analysis (Figure S4A) and are in line with recent findings showing that GATA2 overexpression enhances the generation of mesodermal cells and further promotes EHT (Zhou et al., 2019).

Finally, we performed a new clustering analysis of mesodermal (day 5) and hemato-endothelial single cells (day 7) based on DEGs (1,127 genes) identified in our bulk RNA-seq analysis (HEPs day 10) (Table S4). Using t-SNE and hierarchical clustering to visualize the data, five main clusters were found (Figures S4B and S4C). GO enrichment analysis and over-representation analysis (Boyle et al., 2004) revealed that cluster 0 and cluster 4 were associated with hemato-endothelial progenitors (*ESCR*, *CDH5*, *ERG*, *SOX17*, and *HOXA9*), cluster 3 mesoderm (*TBX3* and *MSX1*), cluster 1 mesenchymal (*FN1*, *LAMA1*, and *COL5A1*), and cluster 2 epiblast (*EPCAM* and *CDH1*) (Figure S4D). These data are in line with the scRNA-seq analysis and suggest that most of the DEGs (1,056 genes) of day 10 were already differentially expressed in our single cells at d5 and d7.

### GATA2 Knockout Inhibits Hematopoietic Development and Favors Cardiomyogenesis

**Figure 5:**
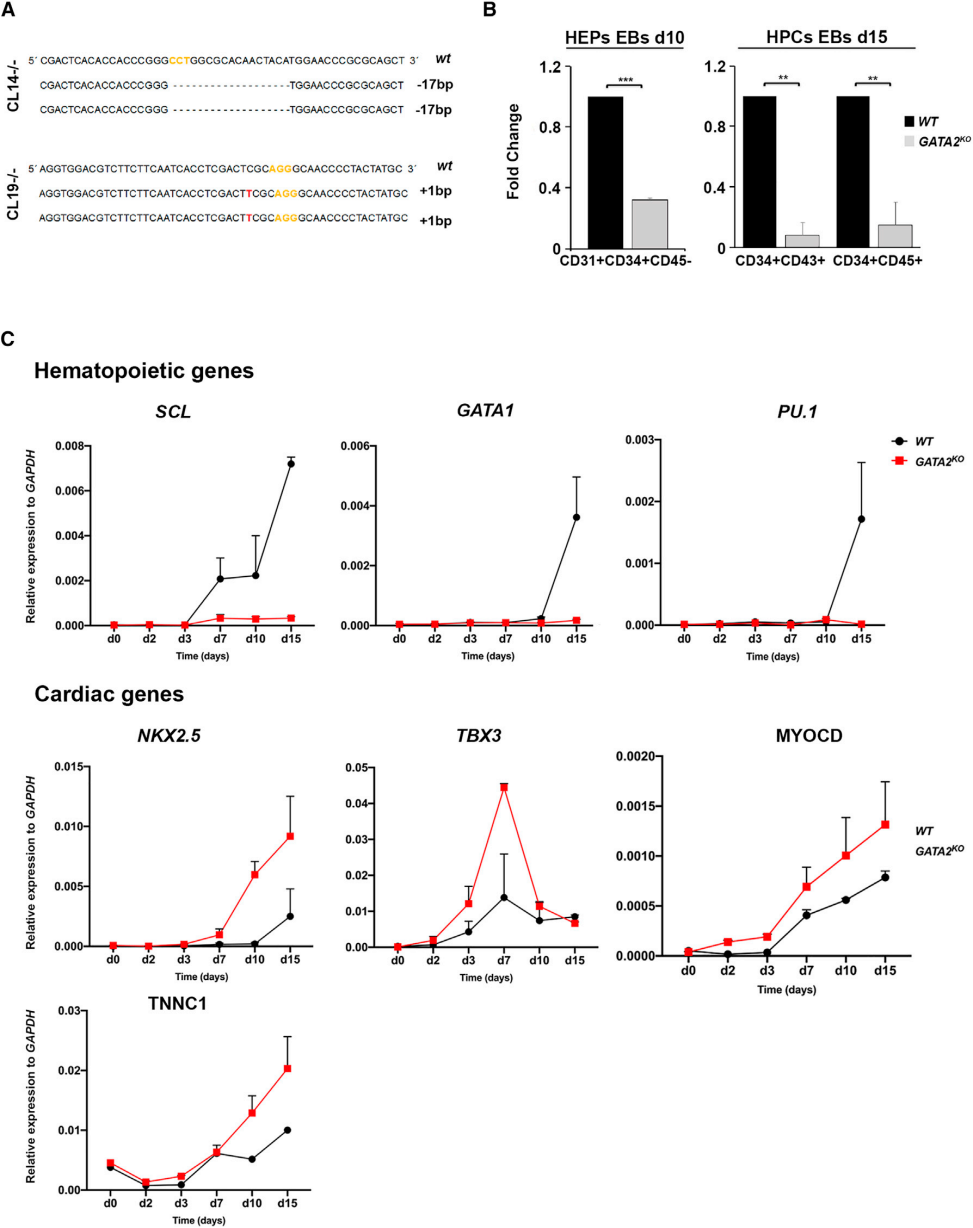
GATA2 Is Essential for HPC Development (A) Representative sequencing of targeted homozygous hiPSC clones at the *GATA2* locus using sgRNA1 and sgRNA2; PAM sequences are labeled in yellow. (B) Quantitative summary of HEP and HPC FACS analysis at d10 and d15 of EB development in wild-type (WT) and hiPSC-GATA2^KO^ clones. Data represent the mean ± SD fold change relative to *WT* iPSC lines. (C) qRT-PCR analysis of hematopoietic and cardiac regulators in WT and hiPSC-GATA^KO^ clones during EB development. Data represent the mean ± SD of 3 independent experiments. ^∗∗^p < 0.01, ^∗∗∗^p < 0.001.

Our results so far strongly suggest that transient GATA2 expression promotes hematopoietic differentiation and represses alternative mesodermal fates during HEP specification. To address whether GATA2 is necessary for specification of HEPs, we used CRISPR/Cas9 gene editing to target exon 2 of *GATA2* and generate knockout hiPSC-GATA2 clones (hiPSC-GATA2^KO^). After expanding individual clones, we selected two targeted clones with biallelic mutations (CL14 and CL19; Figure 5A). Immunocytochemistry and qRT-PCR analysis confirmed the absence of GATA2 expression during hiPSC-GATA2^KO^ differentiation (Figures S5A and S5B). No predicted CRISPR off-targets were detected by genomic sequencing of hiPSC-GATA2^KO^ clones (Figure S5C). The hiPSC-GATA2^KO^ clones retained a normal karyotype and maintained the expression of pluripotency markers (Figures S5D and S5E), confirming that the *GATA2* knockout is compatible with human stem cell pluripotency.

We differentiated wild-type and hiPSC-GATA2^KO^ clones into hematopoietic cells as described earlier (see Figure 1A). As expected, *GATA2* knockout significantly affected HEP generation (CD31^+^CD34^+^CD45^–^) in day 10 EBs (Figure 5B) and markedly decreased the number of HPCs (CD34^+^CD43^+^CD45^+^) in day 15 EBs (Figure 5B). Moreover, we consistently failed to detect hematopoietic CFCs at day 10 of EB development (data not shown).

In line with the transcription and ChIP-seq data, qRT-PCR analysis revealed that the cardiac regulators *TBX3*, *NKX2.5*, and *MYOCD* were progressively upregulated during hiPSC-GATA2^KO^ EB development, whereas the hematopoietic transcription factors *SCL*, *GATA1*, and *PU.1* were markedly suppressed (Figure 5C).

**Figure 6:**
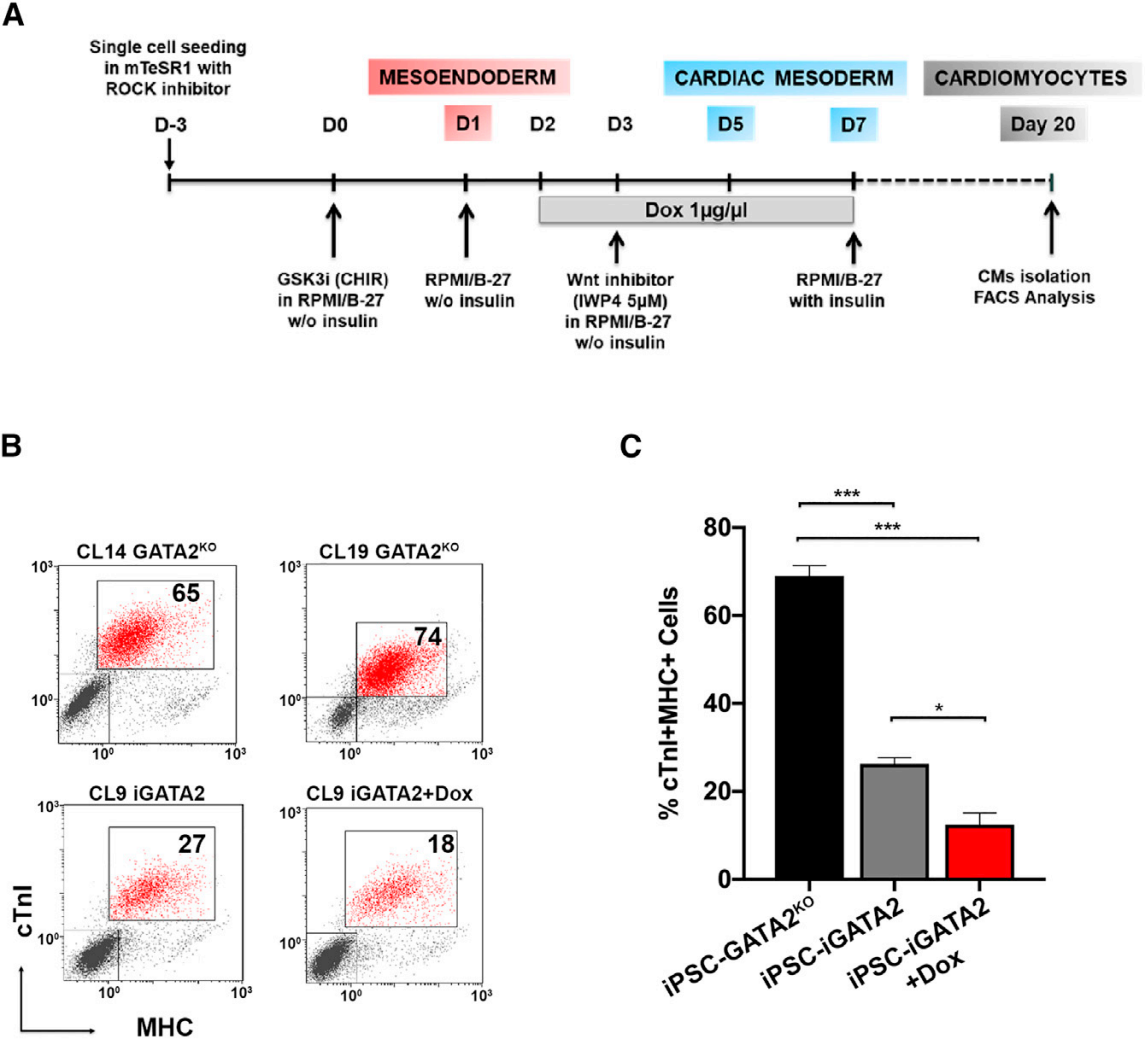
GATA2 Knockout Promotes Cardiomyocyte Differentiation (A) Schematic of the cardiomyocyte differentiation protocol. (B) Representative FACS analysis of the percentage of cTnI^+^ MHC^+^ cells in hiPSC-GATA2^KO^ and iGATA2-iPSC lines (with and without Dox treatment) at day 20 of differentiation. (C) Bar graph showing the mean percentage of cTnI^+^ MHC^+^ cells in (B). Data represent the mean ± SD of 3 independent experiments. ^∗^p < 0.05, ^∗∗∗^p < 0.001.

As these observations strongly suggest that interfering with *GATA2* expression might be an effective strategy to generate cardiomyocytes *ex vivo*, we differentiated hiPSC-GATA2^KO^ and iGATA2-hiPSC lines (with or without Dox administration, days 2–7) to cardiomyocytes using a well-characterized protocol (Lian et al., 2013) (Figure 6A), and measured the expression of the cardiac structural proteins troponin I (cTnI) and myosin heavy chain (MHC) after 20 days. Remarkably, we found a ∼3-fold increase in the number of cTnI^+^ MHC^+^ cells in differentiated hiPSC-GATA2^KO^ cells compared with iGATA2-iPSC cells in the absence of Dox (Figures 6B and 6C). Conversely, Dox administration led to a significant decrease in the number of cTnI^+^ MHC^+^ cells in the iGATA2-iPSC line (Figures 6B and 6C). As a functional readout of cardiomyocyte generation, we monitored for the appearance of beating cells. Whereas the majority of hiPSC-GATA2^KO^ cells started beating at days 8–10 of differentiation and persisted throughout the 20-day experiment (Videos S1 and S2), very few iGATA2-iPSC cells treated with Dox were beating (Video S3). Taken together, these results suggest that GATA2 is both an activator of hematopoiesis and a repressor of cardiac cell fate.

Video S1. hiPSC-GATA2^KO^ CL14 Cardiomyocytes

Video S2. hiPSC-GATA2^KO^ CL19 Cardiomyocytes

Video S3. hiGATA2-iPSC Cardiomyocytes Dox Treated

## Discussion

GATA2 has long been implicated as a master regulator of murine hematopoiesis (Tsai et al., 1994, Tsai and Orkin, 1997), and its dysregulation is associated with human immunodeficiency syndromes (Spinner et al., 2014). However, its role in early human hematopoiesis is less clear. Human iPSCs are a useful model to interrogate the molecular mechanisms driving early hematopoietic development.

To our knowledge, this is the first study showing a dual activity of GATA2 in human early hematopoiesis. We show that GATA2 drives mesoderm progenitors to differentiate into blood cells and represses cardiac fates. Indeed, our *GATA2* knockout model confirmed that loss of GATA2 activity impairs hematopoietic development and enhances cardiomyocyte differentiation.

Earlier studies both *in vivo* and in stem cell models suggested that hematopoietic and cardiac lineages develop in close proximity and are mutually antagonistic (Bussmann et al., 2007, Chagraoui et al., 2018, Chan et al., 2013, Freire et al., 2017, Kouskoff et al., 2005, Liu et al., 2013, Schoenebeck et al., 2007, Van Handel et al., 2012). For example, overexpression of *Scl* in mesodermal cells promotes hematopoietic development at the expense of cardiomyogenesis in differentiated mouse ESCs (Ismailoglu et al., 2008), whereas *Scl*-deficient mice show ectopic cardiomyocytes in yolk salc endothelium and die at E9.5 due to the complete absence of hematopoiesis (Shivdasani et al., 1995, Van Handel et al., 2012). Similarly, *Etv2/Er71* deficiency leads to a complete block of hemato-endothelial development and a concomitant expansion of the cardiac lineage in mutant embryos (Koyano-Nakagawa and Garry, 2017, Lee et al., 2008, Liu et al., 2012, Rasmussen et al., 2011), whereas *Etv2* overexpression in differentiating mouse ESCs shows the opposite phenotype (Liu et al., 2012).

To date, a direct role for GATA2 in cardiac development has not been demonstrated. *Gata2*-null mice have no apparent cardiovascular phenotype, but to our knowledge studies specifically addressing heart malformation in *Gata2* mutants have not been performed. Whether GATA2 overexpression *in vivo* enhances hematopoietic cell specification while concurrently retarding cardiac development should be addressed in the future.

GATA2 is known to cooperate with ER71/ETV2 and SCL/TAL1 to regulate endothelial and hematopoietic programs in stem cells (Elcheva et al., 2014, Shi et al., 2014). The recent observation that coexpression of ER71/ETV2, SCL/TAL1, and GATA2 during mouse ESC differentiation enhances FLK-1^+^ hemangioblast production while blocking cardiac output (Liu et al., 2013) suggests a critical regulatory relationship between these factors during mesoderm diversification. Nevertheless, the gene regulatory network governing hematopoietic and cardiac development is poorly understood. Our bulk RNA-seq data revealed that GATA2 overexpression failed to upregulate ER71/ETV2 expression in our HEP model, which is consistent with the finding that ER71 is expressed before GATA2 during both early mouse and ESC development (Liu et al., 2013). Based on recent studies (Chagraoui et al., 2018, Org et al., 2015), it is reasonable to presume that GATA2 and SCL collaborate to promote blood specification at the expense of cardiac fates. Yet, our deep DNA motif enrichment analysis of GATA2 targets demonstrate the absence of SCL binding sites (CANNTG) in both GATA2-bound activated and repressed genes, which suggests that these two factors—at least in our experimental model—act independently. Of note, recent analysis at the single-cell level revealed that only a subset of mouse E8.5 *Scl*^−/−^ endothelial cells upregulated expression of a few cardiac-related genes. However, those cells did not display a full cardiomyocyte transcriptional program and continued to express key endothelial markers (Pijuan-Sala et al., 2019, Scialdone et al., 2016). Therefore, the role of SCL in the specification of hematopoietic fate and as a cadiac repressor needs to be determined.

ChIP-seq indicated that GATA2 acts more as a repressor than an activator during mesodermal diversification. Specifically, GATA2 binds directly to cardiac regulator promoters, leading to their downregulation. Although previous studies in HPCs and mature blood cells have shown that endogenous GATA2 preferentially occupies sites distant to promoters (Calero-Nieto et al., 2014, Fujiwara et al., 2009, Huang et al., 2016, Wilson et al., 2015), our analysis revealed GATA2 binding promoter regions. Whether this apparent discrepancy is a consequence of cell-type-specific differences in GATA2 occupancy, the fact that GATA2 is overexpressed in our studies, or both, is unclear.

Enforced expression of instructive factors is an accepted strategy to guide lineage fate commitment (Doulatov et al., 2013, Elcheva et al., 2014, Nakajima-Takagi et al., 2013, Navarro-Montero et al., 2017, Ramos-Mejia et al., 2014, Sugimura et al., 2017), and offers the possibility to generate any differentiated cell type from hPSCs. Our study provides cellular and molecular evidence that GATA2 induction promotes an enhancement of mesodermal cells with hemato-endothelial potential, and decreases the probability that alternative mesodermal fates occur. Accordingly, GATA2 could be a target for manipulation to improve the yield of target cells (blood or cardiomyocytes) from hPSCs for drug screening and disease modeling.

In summary, we establish a novel role for GATA2 during mesodermal lineage specification and provide new insights into the complex regulatory network that controls human early hematopoietic development.

## Experimental Procedures

### Human iPSC Culture

Human GATA2-hiPSC and iPSC-GATA2^KO^ lines were maintained on Matrigel-coated 60-mm plates in mTESR1 medium (Giorgetti et al., 2017). Culture medium was changed daily and cells were passaged weekly by EDTA dissociation.

### EB-Based Hematopoietic Differentiation

Human iPSCs were differentiated as described (Giorgetti et al., 2017). In brief, EBs were treated with the GSK3 inhibitor CHIR99021 (3 μM) from day 2 to 3 of culture (Sturgeon et al., 2014). From day 3, EBs were cultured in differentiation medium until day 15. To induce transgene GATA2 expression Dox (1 μg/mL) was added from day 2 to 7 of differentiation.

### Cardiac Differentiation

Human iPSCs were differentiated in monolayer cultures with modulators of canonical Wnt signaling (Lian et al., 2013). Contracting cardiomyocytes could be observed between day 8 and 10 of differentiation. Differentiated cells were disaggregated at day 20 with 0.25% trypsin-EDTA for 5–8 min at 37°C for FACS analysis.

### ChIP-Seq Combined with DNA Massive Sequencing

GATA2-iPSCs (CL6 and CL9) were differentiated in the presence of Dox (from day 2 to 7). ChIP experiments were performed with ∼1.5 × 10^6^ cells using the ChIP-IT High Sensitivity Kit from Active Motif (no. 53040), and specific antibodies for GATA2 (Santa Cruz; sc-9008) or rabbit IgG as isotype control (Abcam; 172730), Libraries were prepared according to Illumina instructions and sequenced using the HiSeq 2000 platform (Illumina).

### Statistical Analysis

All data are expressed as mean ± SD. Statistical comparisons were performed using Student's t test (95% confidence interval). Statistical significance was defined as p < 0.05.

## Author Contributions

Contribution, J.C. conceived the study, designed and performed the experiments, analyzed the data, and wrote the manuscript. S.A., F.J.C.-N., J.L.M., E.M.-R., C.B., E.B., X.W., C.P., L.Z., S.J.-D., E.M., D.R.M., and M.R. performed the experiments and analyzed the data. H.H., E.H.B., B.G., L.d.C., P.M., and A.R. analyzed the data and interpreted the results. A.G. conceived the study, designed and performed experiments, analyzed data, and wrote the manuscript.

## Accession Numbers

The accession number for the ChiP-seq data reported in this paper is GEO: GSE107639; the accession number for the single-cell RNA-seq data is GEO: GSE133996; and the accession number for the RNA-seq data is GEO: GSE118980.

## Supplemental Information

Document S1. Supplemental Experimental Procedures, Figures S1–S5, and Table S1

Table S2. GATA2 Downregulated Genes in HEPs Day 10 EBs, Related to Figure 3

Table S3. scRNA-Seq Marker List for EBs d2, Related to Figure 4

Table S4. Marker List by Cluster Based on DEGs of d10, Related to Figure S4

Document S2. Article plus Supplemental Information
