# Supplementary material for: GATA2 Promotes Hematopoietic Development and Represses Cardiac Differentiation of Human Mesoderm
Source: Stem Cell Reports. 2019 Aug 8;13(3):515–29. doi: 10.1016/j.stemcr.2019.07.009 (PMC6742600; doi:10.1016/j.stemcr.2019.07.009)
Supplement: Document S1. Supplemental Experimental Procedures, Figures S1–S5, and Table S1 [file mmc1.pdf]

Stem Cell Reports, Volume *13*

Supplemental Information

GATA2 Promotes Hematopoietic Development and Represses Cardiac Differentiation of Human Mesoderm

Julio Castaño, Sergi Aranda, Clara Bueno, Fernando J. Calero-Nieto, Eva Mejia-Ramirez, Jose Luis Mosquera, Enrique Blanco, Xiaonan Wang, Cristina Prieto, Lorea Zabaleta, Elisabetta Mereu, Meritxell Rovira, Senda Jiménez-Delgado, Daniel R. Matson, Holger Heyn, Emery H. Bresnick, Berthold Göttgens, Luciano Di Croce, Pablo Menendez, Angel Raya, and Alessandra Giorgetti

Inventory of Supplemental Information

- Figure S1 with Legend
- Figure S2 with Legend
- Figure S3 with Legend
- Figure S4 with Legend
- Figure S5 with Legend
- Table S1
- Table S2
- Table S3
- Table S4
- Movie S1
- Movie S2
- Movie S3
- Supplemental Experimental Procedures
- Supplemental References

Supplemental Figures and legends

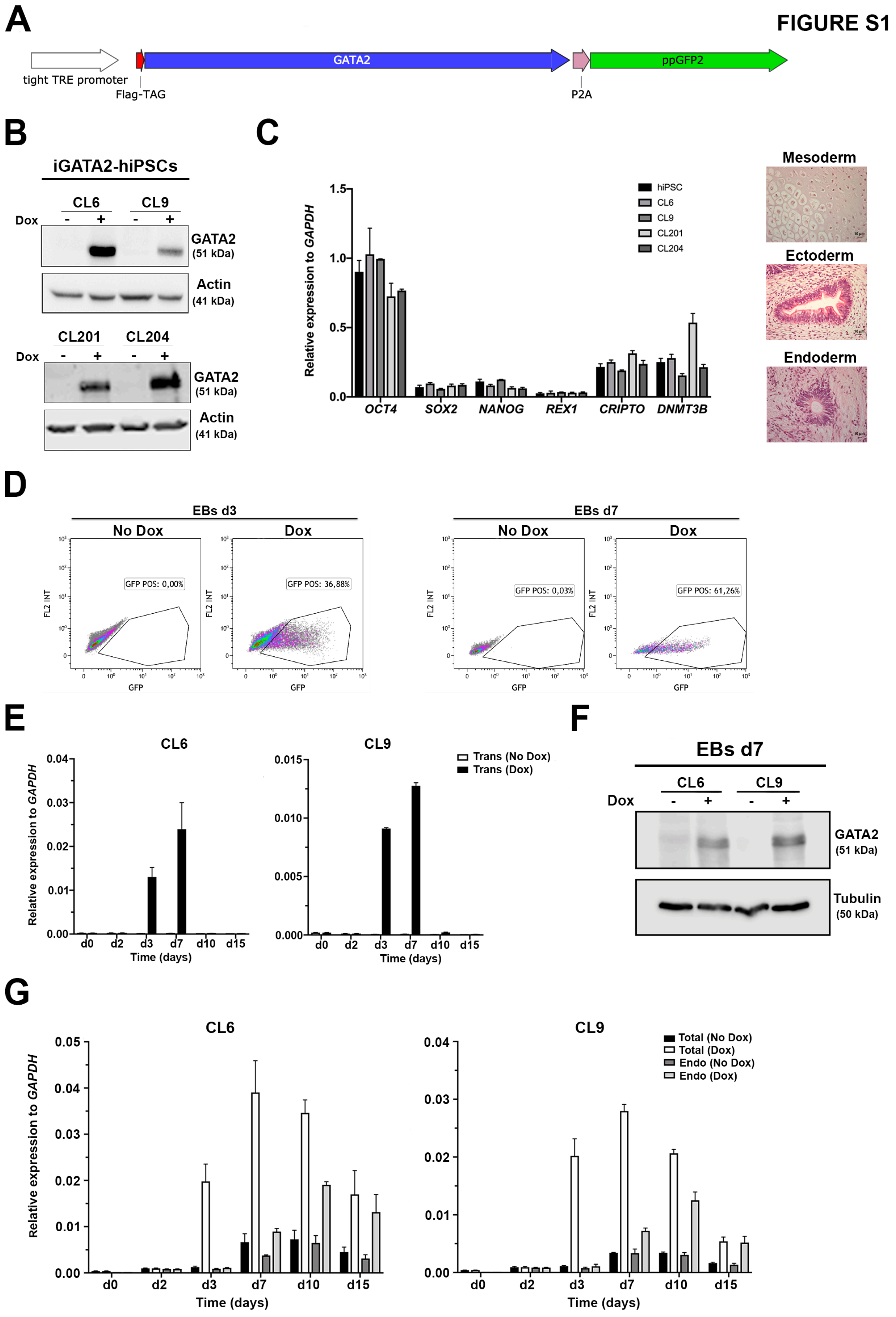

Figure S1. Generation of iGATA2-iPSC lines. Related to Figure 1:
(A) Schematic illustration of the cassette used to generate inducible GATA2-iPSC lines (iGATA2-iPSC). (B) Western blot analysis of GATA2 protein expression in four selected hiPSC lines with and without Dox treatment. (C) qRT-PCR analysis of pluripotency genes (left panel) and teratoma assay (right panel) showing that *GATA2* transgene does not interfere with hiPSC pluripotency. The data represent the mean ± SD of 3 independent experiments. Scale bars are 10 μm. (D) Representative flow cytometry analysis of GFP expression after Dox treatment in EBs d3 and d7. (E) qRT-PCR analysis showing *GATA2* transgene induction in 2 selected hiPSC clones (CL6 and CL9) during EB differentiation. (F) Western blot analysis of GATA2 protein expression in EBs d7 of differentiation for two selected hiPSC lines with and without Dox treatment. (G) qRT-PCR analysis showing endogenous and wild type *GATA2* expression in 2 selected hiPSC clones (CL6 and CL9) during EB differentiation with and without Dox treatment.

A

FIGURE S2

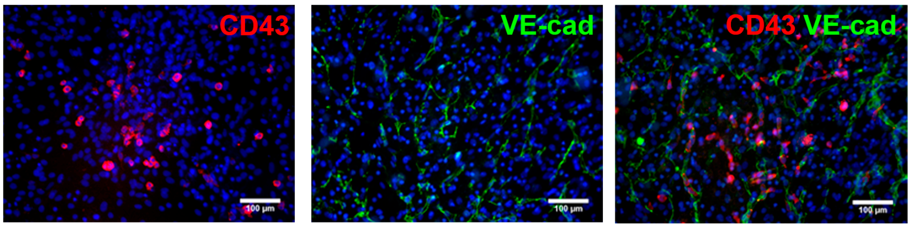

Hematopoietic colonies (HC) **Endothelial colonies (EC)** **Mixed colonies (MC)**

Colonies per 192 wells (KDR+CD31-CD34- EBs d5)

Condition	HC	EC	MC	HC/EC
No Dox	3	8	1	0.4
Dox	5	7	3	0.7

Colonies per 288 wells (CD31+CD34+CD43- EBs d7)

Condition	HC	EC	MC	HC/EC
No Dox	8	11	9	0.72
Dox	12	4	12	3

B

HEPs d10

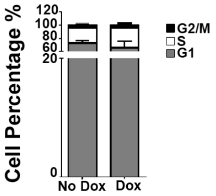

HEPs d10	G1	G2/M	S
No Dox	72.8 ±3.83	4.8±1.19	23.13±3.6
Dox	70.7±2.86	4.53±1.47	26±1.53

HPCs d15

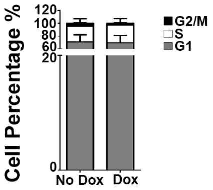

HPCs d15	G1	G2/M	S
No Dox	70.83 ±4.24	4.4±1.11	24.77±11.5
Dox	76.1±2.97	2.93±1.10	27.4±1.10

Figure S2. GATA2 promote hematopoietic differentiation. Related to Figure 2: (A) Representative pictures of hematopoietic (HC), endothelial (EC) and Mixed colonies (MC) derived from single mesodermal cell (KDR+CD31-CD34-; EBs d5) and/or Hemato-endothelial cells (CD31+CD34+CD43-, EBs d7) cultured on OP9 in Dox and No Dox conditions (top table). The number of HC, EC and MC was scored based on CD43 and Ve-cad expression after 7 days of culture (botton table). Scale bars are 100 µm. (B) Flow cytometry analysis of cell cycle of HEPs and HPCs in Dox and No Dox conditions. A similar number of cycling cells (S, G2, and M) were found in HEPs and HPCs of control and Dox-treated cells.

FIGURE S3

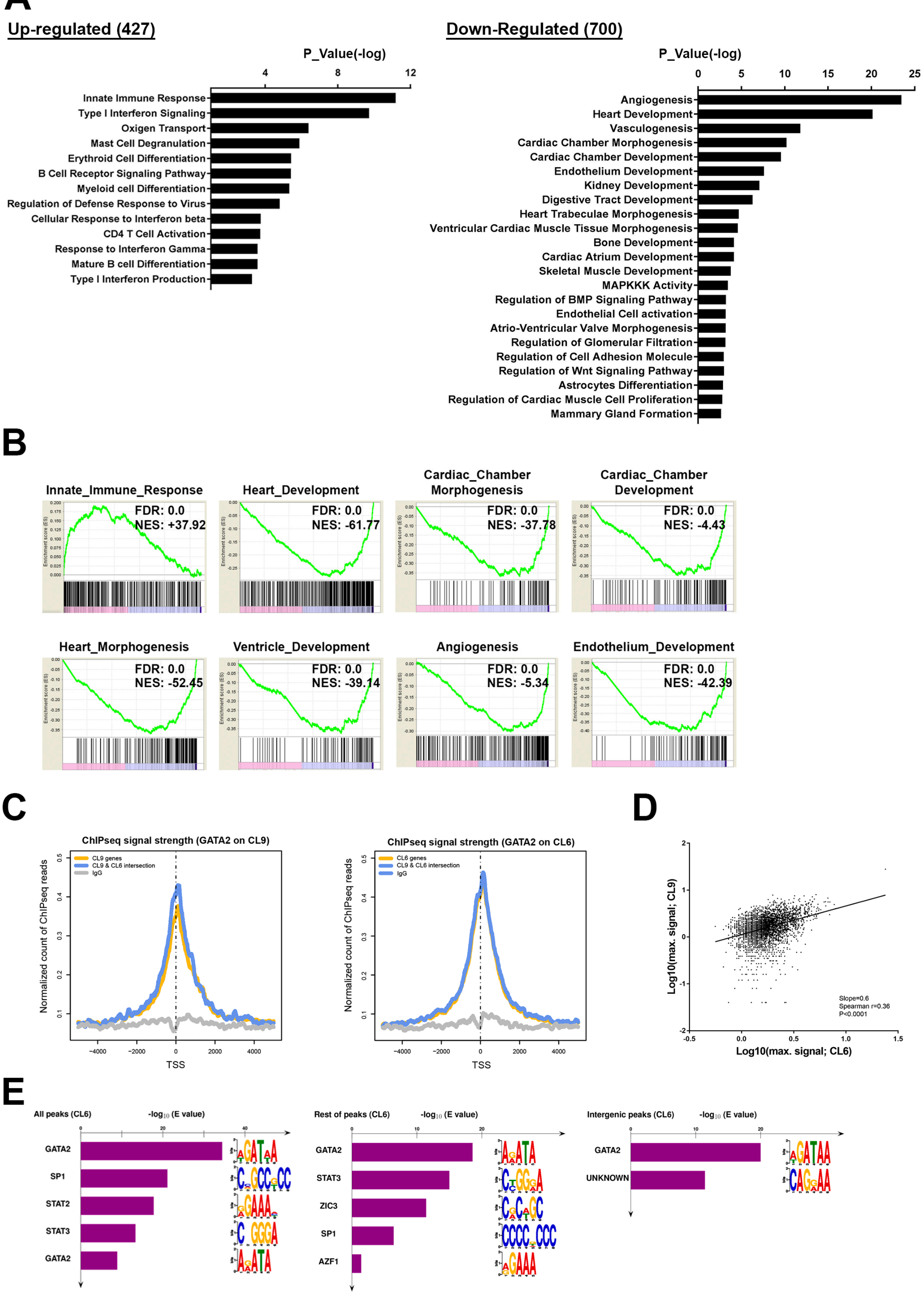
A
Up-regulated (427)
P_Value(-log)
4
8
12
Innate Immune Response
Type I Interferon Signaling
Oxigen Transport
Mast Cell Degranulation
Erythroid Cell Differentiation
B Cell Receptor Signaling Pathway
Myeloid cell Differentiation
Regulation of Defense Response to Virus
Cellular Response to Interferon beta
CD4 T Cell Activation
Response to Interferon Gamma
Mature B cell Differentiation
Type I Interferon Production
Down-Regulated (700)
P_Value(-log)
0
5
10
15
20
25
Angiogenesis
Heart Development
Vasculogenesis
Cardiac Chamber Morphogenesis
Cardiac Chamber Development
Endothelium Development
Kidney Development
Digestive Tract Development
Heart Trabeculae Morphogenesis
Ventricular Cardiac Muscle Tissue Morphogenesis
Bone Development
Cardiac Atrium Development
Skeletal Muscle Development
MAPKKK Activity
Regulation of BMP Signaling Pathway
Endothelial Cell activation
Atrio-Ventricular Valve Morphogenesis
Regulation of Glomerular Filtration
Regulation of Cell Adhesion Molecule
Regulation of Wnt Signaling Pathway
Astrocytes Differentiation
Regulation of Cardiac Muscle Cell Proliferation
Mammary Gland Formation
B
Innate_Immune_Response
FDR: 0.0
NES: +37.92
Heart_Development
FDR: 0.0
NES: -61.77
Cardiac_Chamber Morphogenesis
FDR: 0.0
NES: -37.78
Cardiac_Chamber Development
FDR: 0.0
NES: -4.43
Heart_Morphogenesis
FDR: 0.0
NES: -52.45
Ventricle_Development
FDR: 0.0
NES: -39.14
Angiogenesis
FDR: 0.0
NES: -5.34
Endothelium_Development
FDR: 0.0
NES: -42.39
C
ChIPseq signal strength (GATA2 on CL9)
CL9 genes
CL9 & CL6 intersection
IgG
Normalized count of ChIPseq reads
TSS
ChIPseq signal strength (GATA2 on CL6)
CL6 genes
CL9 & CL6 intersection
IgG
Normalized count of ChIPseq reads
TSS
D
Log10(max. signal; CL9)
Log10(max. signal; CL6)
Slope=0.6
Spearman r=0.36
P<0.0001
E
All peaks (CL6)
-log10 (E value)
GATA2
SP1
STAT2
STAT3
GATA2
Rest of peaks (CL6)
-log10 (E value)
GATA2
STAT3
ZIC3
SP1
AZF1
Intergenic peaks (CL6)
-log10 (E value)
GATA2
UNKNOWN

Figure S3. Gene expression and ChIP-Seq analysis in GATA2 induced Cells. Related to Figure 3: (A) GO function classification analysis of up-regulated and down-regulated genes in GATA2-induced HEPs compared with control cells (HEPs No-Dox). (B) Gene set enrichment analysis (GSEA) showing genes up- and downregulated in HEPs at day 10 of EB differentiation following Dox-inducible *GATA2* expression from day 2 to 7. The false discovery rate (FDR) q value and normalized enrichment score (NES) are shown. (C) Meta-gene plots showing the GATA2 ChIP-seq occupancy profile on CL9 or CL6 replicates for the respective target genes found in each replicate and the common bona fide GATA2 target genes. IgG distribution is indicated with the grey line. (D) Correlation plot between GATA2 ChIP-seq replicates CL6 and CL9 replicates on CL6 peaks found by MACS in CL6. (E) The histograms indicate the Top ranked motifs identified by DREME tool.

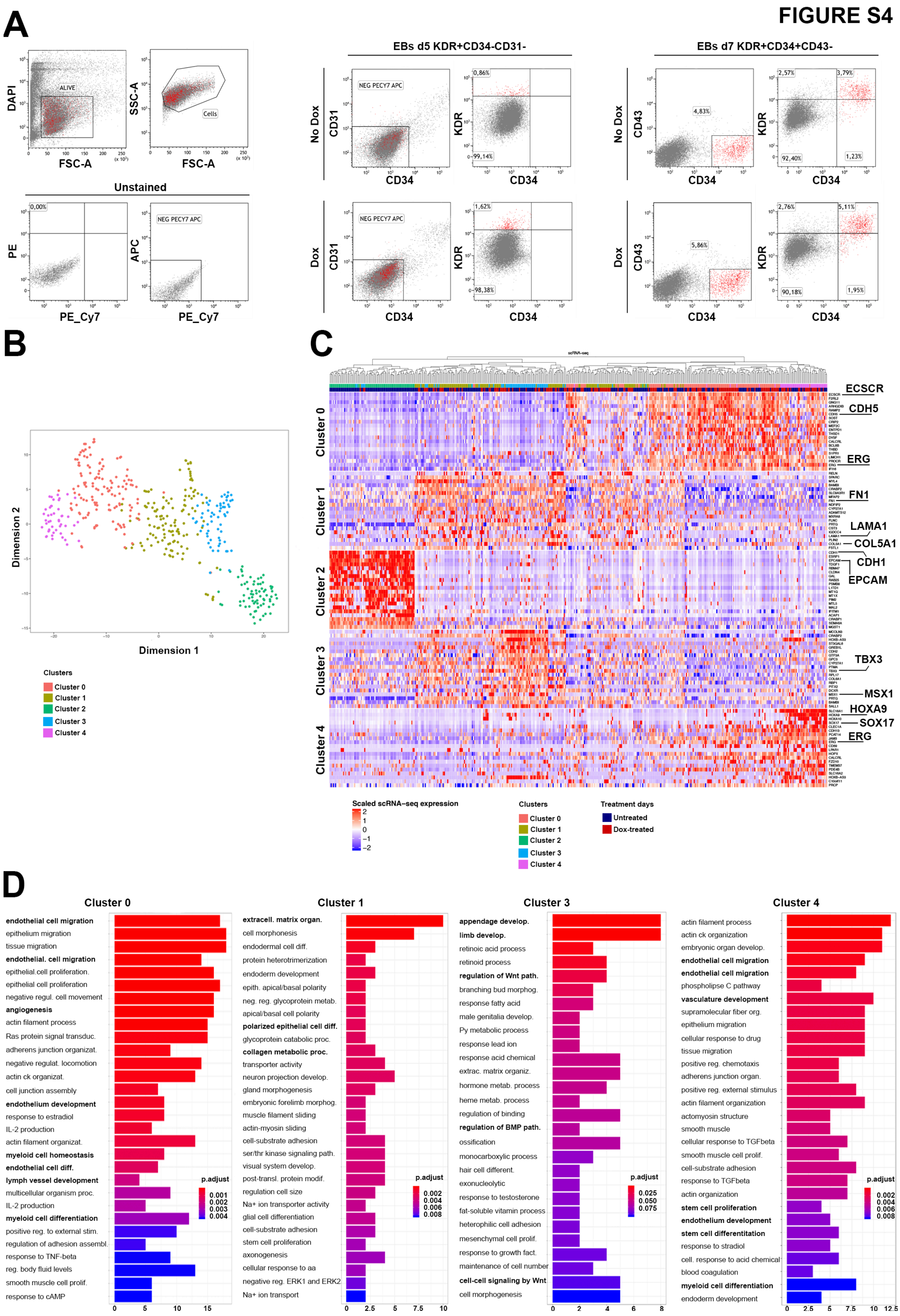
FIGURE S4
A
EBs d5 KDR+CD34-CD31-
EBs d7 KDR+CD34+CD43-
Unstained
No Dox
Dox
DAPI
SSC-A
FSC-A
PE
APC
PE_Cy7
CD31
CD34
KDR
CD43
B
Dimension 1
Dimension 2
Clusters
Cluster 0
Cluster 1
Cluster 2
Cluster 3
Cluster 4
C
ECSCR
CDH5
ERG
FN1
LAMA1
COL5A1
CDH1
EPCAM
TBX3
MSX1
HOXA9
SOX17
ERG
Scaled scRNA-seq expression
Treatment days
Untreated
Dox-treated
D
Cluster 0
endothelial cell migration
epithelium migration
tissue migration
endothelial. cell migration
epithelial.cell proliferation.
epithelial cell proliferation
negative regul. cell movement
angiogenesis
actin filament process
Ras protein signal transduc.
adherens junction organizat.
negative regulat. locomotion
actin ck organizat.
cell junction assembly
endothelium development
response to estradiol
IL-2 production
actin filament organizat.
myeloid cell homeostasis
endothelial cell diff.
lymph vessel development
multicellular organism proc.
IL-2 production
myeloid cell differentiation
positive reg. to external stim.
regulation of adhesion assembl.
response to TNF-beta
reg. body fluid levels
smooth muscle cell prolif.
response to cAMP
Cluster 1
extracell. matrix organ.
cell morphogenesis
endodermal cell diff.
protein heterotrimerization
endoderm development
epith. apical/basal polarity
neg. reg. glycoprotein metab.
apical/basal cell polarity
polarized epithelial cell diff.
glycoprotein catabolic proc.
collagen metabolic proc.
transporter activity
neuron projection develop.
gland morphogenesis
embryonic forelimb morphog.
muscle filament sliding
actin-myosin sliding
cell-substrate adhesion
ser/thr kinase signaling path.
visual system develop.
post-transl. protein modif.
regulation cell size
Na+ ion transporter activity
glial cell differentiation
cell-substrate adhesion
stem cell proliferation
axonogenesis
cellular response to aa
negative reg. ERK1 and ERK2
Na+ ion transport
Cluster 3
appendage develop.
limb develop.
retinoic acid process
retinoid process
regulation of Wnt path.
branching bud morphog.
response fatty acid
male genitalia develop.
Py metabolic process
response lead ion
response acid chemical
extrac. matrix organiz.
hormone metab. process
heme metab. process
regulation of binding
regulation of BMP path.
ossification
monocarboxylic process
hair cell different.
exonucleolytic
response to testosterone
fat-soluble vitamin process
heterophilic cell adhesion
mesenchymal cell prolif.
response to growth fact.
maintenance of cell number
cell-cell signaling by Wnt
cell morphogenesis
Cluster 4
actin filament process
actin ck organization
embryonic organ develop.
endothelial cell migration
endothelial cell migration
phospholipse C pathway
vasculature development
supramolecular fiber org.
epithelium migration
cellular response to drug
tissue migration
positive reg. chemotaxis
adherens junction organ.
positive reg. external stimulus
actin filament organization
actomyosin structure
smooth muscle
cellular response to TGFbeta
smooth muscle cell prolif.
cell-substrate adhesion
response to TGFbeta
actin organization
stem cell proliferation
endothelium development
stem cell differentitation
response to stradiol
cell. response to acid chemical
blood coagulation
myeloid cell differentiation
endoderm development
p.adjust

Figure S4. GATA2 induction enhances mesodermal cell generation and the activation of hemato-endothelial program. Related to Figure 4:

(A) Representative Flow cytometry analysis of mesodermal (KDR+CD31-CD34-) and hemato-endothelial (KDR+CD43+CD45-) phenotype in EBs at d5 and d7, following Dox-inducible GATA2 expression from day 2 to 7 of differentiation. (B) t-SNE projection of single cell, based on new clustering using DEGs of d10. (C) Hierarchical clustering showing the expression in single cells of the top 20 DEGs identified (adjusted p-value < 0.05) at d10 from bulk RNA-seq. Colored top bar indicates assigned cluster and treatment. Red indicates the highest scaled expression and blue the lowest. (D) Over Representation Analysis (ORA) showing enrichment of genes within GO biological processes for each indicated clusters. Size of the bar represents the number of DEGs for each GO category. The color represents the adjusted p-value for each GO category.

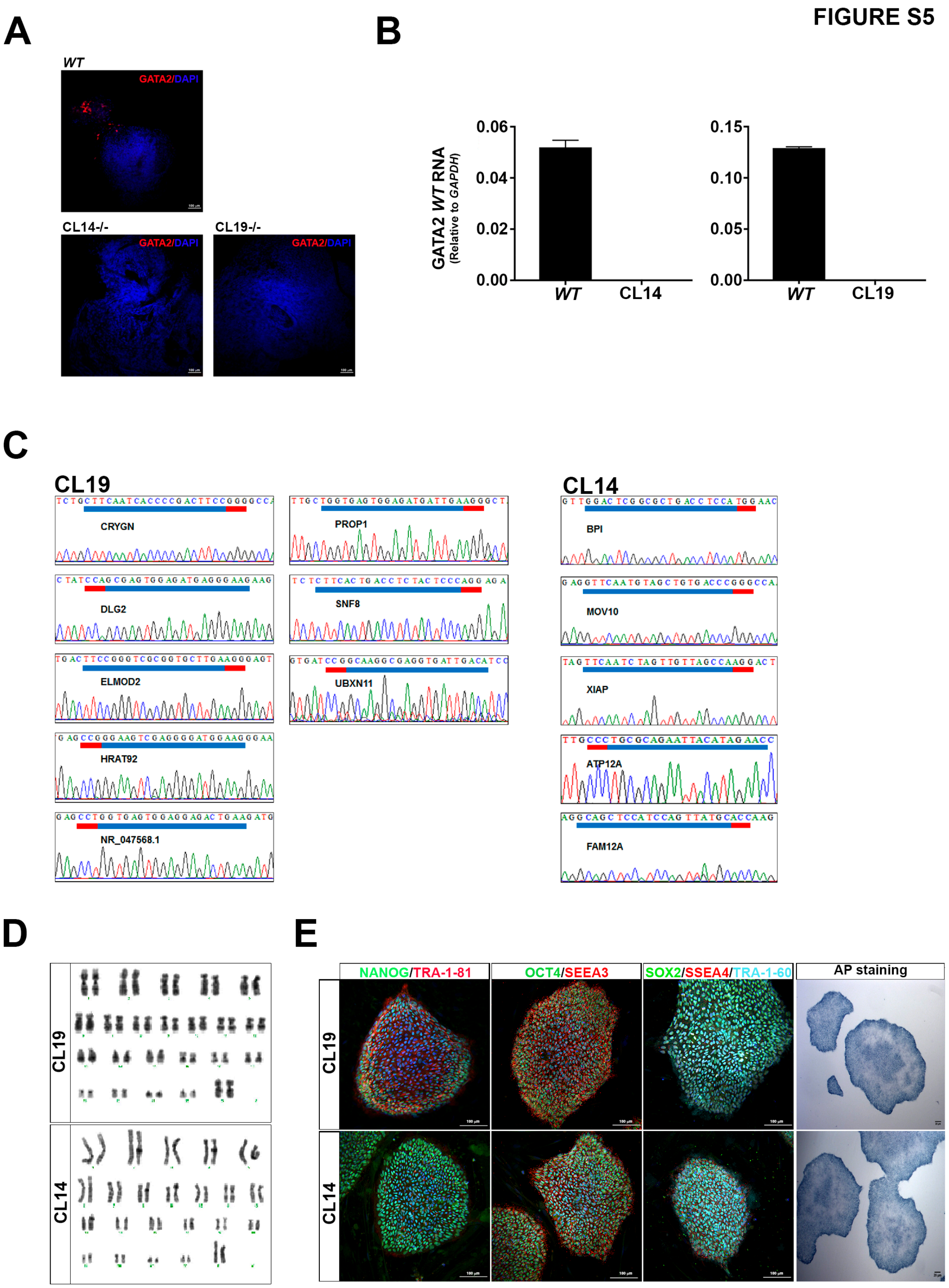

Figure S5. Generation of GATA2^{KO} hiPSC lines. Related to Figure 5: (A) Representative immunofluorescence images of wild-type (WT) and hiPSC-GATA2^{KO} EBs at day 15 of development. GATA2 protein was absent in hiPSC-GATA2^{KO} clones. Scale bars are 100µm. (B) qRT-PCR analysis showing endogenous GATA2 knockout in 2 selected hiPSC clones at day 15 of EB development. (C) Representative histograms of the putative off-target genes in the two selected GATA2^{KO} clones. Blue lines indicate sgRNA homology to target sequence, red lines the PAM sequences. (D and E) Established hiPSC-GATA2^{KO} clones showed normal karyotypes (D) and retained the expression of pluripotency markers (E). Scale bars are 100 µm (pluripotency markers) and 20 µm (AP staining).

Supplemental Tables

Table S1: Primers used for qRT-PCR analysis

Name	Forward primer (5'to 3')	Reverse primer (5' to 3')
**hGAPDH**	GCACCGTCAAGGCTGAGAAC	AGGGATCTCGCTCCTGGAA
**Pluripotency**		
**hCRIPTO**	CGGAACTGTGAGCACGATGT	GGGCAGCCAGGTGTCATG
**hDNMT3B**	GCTCACAGGGCCCGATACTT	GCAGTCCTGCAGCTCGAGTTTA
**hNANOG**	ACAACTGGCCGAAGAATAGCA	GGTTCCCAGTCGGGTTCAC
**hOCT4**	GGGTTTTTGGGATTAAGTTCTTCA	GCCCCCACCCTTTGTGTT
**hREX1**	CCTGCAGGCGGAAATAGAAC	GCACACATAGCCATCACATAAGG
**hSOX2**	CAAAAATGGCCATGCAGGTT	AGTTGGGATCGAACAAAAGCTATT
**Hematopoietic**		
**GATA2 TRANS**	ACTACAAGGACGACGATGACAAG	CGAGTCGAGGTGATTGAAGAAGA
**hGATA2 3'**	AGCTTTACTGTGGCTGTCTGGAT	CCGTCACCGCATACAGAATCTA
**hBRACHYURY**	ATGAGCCTCGAATCCACATAGT	TCCTCGTTCTGATAAGCAGTCA
**hGATA1**	ACCTGCACTGCCTTCATCACT	AAAGTCAGGGCCCCCATAAG
**hMIXL1**	CCGAGTCCAGGATCCAGGTA	CCACTCTGACGCCGAGACTT
**hPU.1**	TGCAAAATGGAAGGGTTTCC	GTCATAGGGCACCAGGTCTTCT
**hRUNX1**	GTGGTCCTATTTAAGCCAGCCC	CTGAAGACACCAGCTTGACAGTTC
**hSCL/TAL1**	GCGGGCCCAGCACCTTT	ATGCAGAGACCAACGCAATTC
**hSOX17**	TGGCGCAGCAGAATCCA	CCACGACTTGCCCAGCAT
**Cardiac**		
**hMYOCD**	ACCAGTCAGATGCGGGGAA	CCAAGGATTTGGACTTTACAGCA
**hNKX2.5**	CTTCTATCCACGTGCCTACAGC	CGCACAGCTCTTTCTTTTCGG
**hTBX3**	CACCAACAACATTTCAGACAAACA	TGCCACGTAGCGTGATCACT
**hTNNC1**	GGACGACAGCAAAGGGAAATCT	TCCAGGTCGATGTAGCCATCAG

Supplemental Experimental Procedures

Human iPSC culture

Human iPSCs were generated from CB-derived CD133+ cells using integrative (retrovirus, line CBI08#4) and non-integrative vectors (Sendai virus, line CBI01#40-Sv), as described (Arellano-Viera et al., 2019; Ban et al., 2011; Giorgetti et al., 2009). Human iPSC lines were maintained in a feeder-free culture system on Matrigel (BD Biosciences)-coated 60-mm plates in mTESR1 medium (StemCell Technologies). The culture medium was changed daily and cells were passaged weekly by dissociation with EDTA. Approval from the Spanish National Embryo Ethical Committee was obtained to work with hPSCs.

Genome engineering

Generation of inducible GATA2-hiPSC lines was performed using the Lenti-X™ Tet-On® Advanced Inducible Expression System (Clontech; Cat#632162). GATA2 cDNA was PCR amplified and cloned in frame with a vector containing a P2A-GFP cassette. GATA2-GFP was subcloned with EcoRI in pLVX-TRE-tight vector. CMV promoter of the pTet-On Advanced vector was replaced by Ubiquin C promoter in order to avoid promoter silencing in hiPSCs (Figure S1A). Cells were transduced by lentivirus infection at a multiplicity of infection of 15, and single-cell-derived clones carrying GATA2 transgene were selected in puromycin (0.25 µg/mL) and neomycin (75 µg/mL).

GATA2-knockout hiPSCs (iPSC-GATA2^{KO}) were generated using CRISPR/Cas9 technology. The CRISPR Design Tool (http://tools.genome-engineering.org) was used for guide RNA design, and the site-specific cleavage efficiency of up to four guide RNAs was tested in 293T cells using the T7 surveyor assay (Ran et al., 2013). Undifferentiated hiPSCs were treated with 10 µM ROCK inhibitor (Y27632; Sigma) for 24 hours and 2×10^5 cells were electroporated using the Neon® Transfection System 10 µL Kit (Thermo Fisher Scientific). Cells were transfected with 5 µg of pSpCas9(BB)-2A-GFP (PX458) (Addgene; #48138, www.addgene.org), allowing the expression of both the guide RNA and the Cas9-GFP. At 3 days post-transfection, cells were GFP-sorted and plated as single cells on Matrigel-coated 48-well plates. Single-cell-derived clones were expanded and screened by sequencing. Previously predicted off-targets identified using CRISPR Design Tool were analyzed in iPSC-GATA2^{KO} cells by Sanger sequencing.

Embryoid body-based hematopoietic differentiation

Human iPSCs were differentiated as previously described (Giorgetti et al., 2017). Briefly, for EB generation, undifferentiated hiPSCs were dissociated with collagenase IV, scraped off gently from the Matrigel, transferred to low-attachment plates, and incubated overnight in mTESR1 medium supplemented with bone morphogenetic protein 4 (BMP-4; 50 ng/mL). The following day (day 1), the medium was changed for serum-free defined medium (StemPro-34; Invitrogen) supplemented with monothioglycerol (0.16 µM; Sigma), holo-transferrin (150 µg/mL; Sigma), 50 ng/mL BMP-4, and 10 ng/mL FGFb (both purchased from R&D Systems). To promote the definitive hematopoietic program, EBs were treated with the GSK3 inhibitor CHIR99021 (3 µM) from day 2 to day 3 of culture (Sturgeon et al., 2014). On the third day of differentiation, EBs were changed to differentiation medium comprising serum-free defined medium (StemPro-34; Invitrogen) supplemented with monothioglycerol, holo-transferrin, 50 ng/mL BMP-4, 300 ng/mL Fms-related tyrosine kinase 3 ligand (Flt-3L), 300 ng/mL stem cell factor (SCF), 10 ng/mL IL-3 and 10 ng/mL IL-6 (all purchased from R&D Systems). EBs were cultured until day 15 with medium changes every 3 days. Dissociated cells were analyzed by flow cytometry for the presence of HEPs (CD31+CD34+CD45- and CD34+CD43-CD45-), early HPCs (CD34+CD43+CD45+), and mature blood cells (CD45+), at day 10 and day 15 of differentiation.

Hemogenic endothelial progenitors/ OP9 cocultures

Hemogenic endothelial progenitor cell-potential was assayed as described (Ramos-Mejia et al., 2014). Briefly, day 10 EBs were dissociated and HEPs were purified by magnetic bead separation using the CD34 Microbead Kit (Miltenyi). Purified HEPs (3×10^4) were plated on OP9 stroma on 6-well plates with differentiation medium. Cells were harvested at day 4 and analyzed by FACS as described above.

Clonal experiments with FACS-sorted single mesodermal and hemato-endothelial cells

Single mesodermal (KDR+CD34-CD31-) and hemato-endothelial (CD31CD34+CD43-) cells FACS sorted from day 5 and day 7 EBs respectively, were transferred to individual wells of 96-well plates containing OP9 feeder cells. The cells were cultured for up to 10 days with hematopoietic differentiation medium with/without Dox. Fresh medium was provided every other day. Culture plates were fixed and

stained with CD144 (rabbit, eBioscience) and anti-CD43 (mouse, BD Bioscience). Hematopoietic, endothelial and mix clusters were analyzed using a fluorescent microscope.

Flow cytometry analysis and FACS

Differentiating EBs were dissociated with collagenase B (Roche) for 2 hours at 37°C and then incubated with Cell Dissociation Buffer (Invitrogen) for 20 minutes at 37°C. Single-cell suspensions were stained with anti-CD31-APC (Miltenyi), anti-CD34-PE, anti-CD45-APC-H7, anti-CD43-APC, anti-CD34-PE-Cy7 (all from BD Biosciences). hiPSC-OP9-cocultures were treated for 1 hour with collagenase IV, incubated for 20 minutes with TrypLE (Invitrogen), dissociated by pipetting, and filtered through a 70-μm cell strainer. Single-cell suspensions were stained with anti-CD34-PE, anti-CD31-APC, anti-CD45-APC-H7, and anti-mouse CD29-FITC (Miltenyi) to exclude OP9 cells from further analysis. Cardiomyocytes were stained with anti-MHC-PE (IgG2b, 1:400 BD Biosciences) and anti-cTnI-Alexa Flour 647 (IgG2b, 1:100, BD Biosciences). Mouse IgG2b PE (1:400 BD Biosciences) and mouse IgG2b Alexa Fluor 647 (1:100, BD Biosciences) antibodies were used as isotype controls.

Live cells identified by propidium iodide (PI) exclusion were analyzed on a Flow Cytometer (Gallios, Beckman Coulter) equipped with Kaluza analysis software, or on a FACSCanto II cytometer equipped with FACS Diva analysis software (BD Biosciences). Sorting of purified mesodermal and hemato-endothelial cells was performed using a FACSAria cell sorter (BD Biosciences), as described (Ramos-Mejia et al., 2014).

CFU assay

CFU assays were performed by plating 50000 cells from day 10 EBs onto serum-free methylcellulose (MethoCult® SF H4436; StemCell Technologies). Colonies were counted 14 days later using standard morphological criteria.

Cell cycle analysis

EBs at day 10 and day 15 were dissociated and fixed in 70% cold ethanol and stored at -20°C. The next day, the cells were stained with anti-CD34-FITC, anti-CD31-FITC, and anti-CD45-APC for 15 minutes. After washing, the cells were suspended in PBS and 50 μg/mL PI. Cell cycle distribution was analyzed on a FACSCanto II cytometer using Motif software. Cell proliferation was assessed using the Click-iT Plus EdU cell proliferation kit (molecular Probe). EBs were incubated with 10 μM 5-ethylnyl-2'-deoxyuridine (EdU) for 5 hours, dissociated and single-cell suspensions were analyzed by flow cytometry as previously described.

Apoptosis analysis

Apoptosis in HEPs (CD31+CD34+CD45-) and HPCs (CD34+CD45+) was analyzed by flow cytometry using the Annexin V Apoptosis Detection Kit (BD Biosciences).

Western blotting

Human iPSC lines and day 7 differentiating EBs were collected, washed in PBS and lysed in 50 μl RIPA Buffer (Tris-HCl pH 8, 50 mM, NaCl 150 mM, 1% NP-40, 0.5% deoxycholate, 0.1% SDS) with 0.25 mM PMSF and complete protease inhibitor cocktail (Roche) for 30 minutes on ice. Lysates were centrifuged at maximum speed for 10 minutes at 4ºC. Protein supernatant extracts were quantified using the Bio-Rad Protein Assay. For electrophoretic separation, 40 μg of protein was run on 8% SDS-polyacrylamide gels and transferred to PVDF membranes that were then blocked with 5% BSA (Sigma) in TBS / 0.5% Tween-20 (TBS-T), and incubated overnight at 4ºC with the appropriate antibody in TBS-T with 5% BSA. Membranes were washed with TBS-T three times and incubated with an HRP-conjugated secondary antibody for 1 hour. Immunoreactive proteins were detected using the Amersham ECL Prime Western Blotting Detection Reagent (GE Healthcare). Primary antibodies used were anti-GATA2 rabbit polyclonal antibody (Im et al., 2005), anti-GATA2 (Cell Signaling Technologies #4595; rabbit 1:500), anti-β actin (Sigma; mouse 1:5000) and anti-Tubulin (Sigma, mouse 1:500).

RNA purification and quantitative RT-PCR

Total RNA was isolated using the RNAqueous-Micro Kit (Ambion). RNA (1 μg) was retrotranscribed to cDNA using the SuperScript III Reverse Transcriptase Kit (Invitrogen). Quantitative real-time PCR (qPCR) was performed using SYBR Green or IDT PrimeTime chemistry. Expression of *GAPDH* was used to normalize data. Primer sequences are listed in Table S1.

RNA-sequencing

HEPs were FACS-purified from three independent biological replicates (CL6, CL9 and CL201) in Dox and no Dox conditions. For each clone, we analyzed three different pools of 50 HEP cells in Dox and No Dox conditions. Pools of 50 cells were sorted directly into 2.3 µL of lysis buffer (0.2 % (v/v) Triton X-100 and 2 U/µL RNase inhibitor (Clontech)) in 96-well plates and stored at -80°C. RNA-seq was carried out using the Smart-seq2 protocol according to Picelli et al. (Picelli et al., 2014). Libraries were prepared using the Illumina Nextera XT DNA preparation kit. Pooled libraries were sequenced on the Illumina Hi-Seq 2500 platform. Reads were mapped simultaneously to the *Homo sapiens* genome (version GRCh38.81) and the ERCC sequences using STAR (version 2.5.2a) (Dobin et al., 2013) with default parameters. HTseq-count (Anders et al., 2015) was used to count the number of reads mapped to each gene (default options). Samples with less than 500,000 reads mapping to endogenous RNA, with greater than 20% reads mapping to mitochondrial genes or with less than 8000 genes detected at 10 counts per million were considered low quality and removed from downstream analyses. All samples passed quality control. The data were normalized using size factors (Love et al., 2014) defined by DESeq2. Highly variable genes were identified as described (Brennecke et al., 2013). In brief, we fitted the squared coefficient of variation as a function of the mean normalized ERCC counts and selected the genes that exceeded 50% of biological coefficient of variation. In the fitting procedure, to minimize the skewing effect due to the low expressed genes, only ERCCs with a mean normalized count greater than the 80th centile were used. Genes with an adjusted *p*-value (Benjamini-Hochberg method) less than 0.1 were considered significant. This resulted in 501 highly variable genes. PCA and tSNE dimensionality reduction were performed using prcomp (Krijthe, 2015; Venables and Ripley, 2002) package in R. Differentially expressed genes were identified using DESeq2 (1.16.1) (Love et al., 2014) for all genes with a false discovery rate (Benjamini-Hochberg) lower than 0.1.

Single-cell RNA sequencing

Full-length single-cell RNA sequencing libraries were prepared using the Smart-seq2 protocol (Picelli et al., 2014) with minor modifications. Briefly, freshly harvested single cells were sorted into 96-well plates containing the cell lysis buffer. Reverse transcription was performed using SuperScrpit II (Invitrogen) in the presence of oligo-dT30VN, the template-switching oligonucleotide and betaine. The cDNA was amplified using the KAPA Hifi Hotstart ReadyMix (Kappa Biosystems), ISPCR primer and 24 cycles of amplification. Following purification with Agencourt Ampure XP beads (Beckmann Coulter), product size distribution and quantity were assessed on a Bioanalyzer using a High Sensitvity DNA Kit (Agilent Technologies). In total, 140 pg of the amplified cDNA was fragmented using Nextera® XT (Illumina) and amplified with indexed Nextera® PCR primers. Products were purified twice with Agencourt Ampure XP beads and quantified again using a Bioanalyzer High Sensitvity DNA Kit. Sequencing of Nextera® libraries from 384 cells was carried out using one sequencing lane on a Illumina HiSeq2500 v4. The sequencing data were aligned with STAR against the GRCh38 human reference. Cells with less than 65% of mapped reads or a read depth lower than four median absolute deviations from the median of total mapped reads were discarded. Genes expressed in fewer than five cells were also removed. Read counts from 444 single cells (91% of total cells) were used to perform the downstream analyses. Normalization, clustering, gene marker detection and visualization were performed using the Seurat package (version 2.3.4). The number of principal components was selected according to their significance and the resolution parameter set to 0.6, resulting in cluster numbers reflecting the expected biological variability. Cluster-specific markers were then identified based on a Wilcoxon rank-sum test. Chi-squared tests were used for comparing differences between treatments, and the resulting raw *p*-values where adjusted by the Benjamini-Hochberg false discovery rate (FDR) method (Benjamini and Hochberg, 1995).

Bioinformatics analysis

ChIP-seq samples were mapped against the hg19 human genome assembly using Bowtie with the option –m 1 to discard those reads that could not be uniquely mapped in just one region (Langmead et al., 2009). MACS was run with the default parameters but adjusting the shiftsize to 100 bp, to perform the peak calling against the corresponding control sample (Zhang et al., 2008). The UCSC genome browser (Tyner et al., 2017) was used to provide screenshots. The genome distribution of each set of peaks was calculated by counting the number of peaks fitting on each class of region according to RefSeq annotations. The distal region was defined as the region within 2.5 kbp and 0.5 kbp upstream of the transcriptional start site (TSS), whereas the proximal region was defined as the region within 0.5 kbp and the TSS. Those peaks overlapping more than one genomic feature were proportionally counted the same number of times. To generate the spie charts, we first calculated the genome distribution of all features in the full genome and then we used the Caroline package in R to combine the spie chart of each set of peaks with the full genome distribution (Feitelson, 2003).

Each set of target genes was retrieved by matching the ChIP-seq peaks in the region 2.5 kbp upstream of the TSS until the end of the transcript, as annotated in RefSeq. The Enrichr tool was used to generate the reports of functional GO enrichments and other categories (Kuleshov et al., 2016).

The meta-gene plots showing the average distribution of ChIP-seq reads along the region between the TSS and the transcription end site of each target gene were generated by counting the number of reads on this region and normalizing by the total number of mapped reads of the ChIP-seq sample and the length of the gene according to RefSeq. This value was finally averaged for the total number of genes in the particular gene set.

Motif analysis of the resulting peaks and promoter sequences was performed with the MEME-ChIP software of the MEME suite of programs (Machanick and Bailey, 2011).

Luciferase assay

PITX2 and ISL1 promoter regions were amplified by PCR from human genomic DNA and inserted into the KpnI and NheI sites of pGL-3 Basic (Promega, Madison, WI) to generate reporter plasmids. The nucleotide sequences of primer sets are: PITX2 Fw: GGTACCTGACAAGCCTAGCTCGTTCG and Rv: GCTAGCGGCTCCCAGGTAATTCGCTT; ISL1 Fw: GGTACCCCTAGGTCTATCCAACTTTCGC and Rv: GCTAGCTTCACATCTCTGGGCATTGACTG). The pGL3-TBX3-3′ UTR vector was kindly provided by Prof. Sharon Prince, Cancer Research Laboratory University of Cape Town (Peres et al., 2017). The plasmid pWPI-GATA2, available in our laboratory, and its empty vector pWPI, were used in this study. For luciferase assays, COS7 cells were transfected with 120 ng of reporter plasmid, 500 ng of expression plasmid, and 10 ng of pRL-null (Promega) using Lipofectamine 3000 (ThermoFisher Scientific) and harvested 48 hours after transfection. Luciferase activity was analyzed using Biotek and a dual-luciferase assay kit (Promega).

Each experiment was performed in triplicate, while all experiments were repeated at least three times.

Supplemental References

Anders, S., Pyl, P.T., and Huber, W. (2015). HTSeq--a Python framework to work with high-throughput sequencing data. Bioinformatics *31*, 166-169.

Arellano-Viera, E., Zabaleta, L., Castano, J., Azkona, G., Carvajal-Vergara, X., and Giorgetti, A. (2019). Generation of two transgene-free human iPSC lines from CD133(+) cord blood cells. Stem Cell Res *36*, 101410.

Ban, H., Nishishita, N., Fusaki, N., Tabata, T., Saeki, K., Shikamura, M., Takada, N., Inoue, M., Hasegawa, M., Kawamata, S.*, et al.* (2011). Efficient generation of transgene-free human induced pluripotent stem cells (iPSCs) by temperature-sensitive Sendai virus vectors. Proc Natl Acad Sci U S A *108*, 14234-14239.

Benjamini, Y., and Hochberg, Y. (1995). Controlling the False Discovery Rate: A Practical and Powerful Approach to Multiple Testing. Journal of the Royal Statistical Society, Series B (Methodological) *57*, 12.

Brennecke, P., Anders, S., Kim, J.K., Kolodziejczyk, A.A., Zhang, X., Proserpio, V., Baying, B., Benes, V., Teichmann, S.A., Marioni, J.C.*, et al.* (2013). Accounting for technical noise in single-cell RNA-seq experiments. Nat Methods *10*, 1093-1095.

Dobin, A., Davis, C.A., Schlesinger, F., Drenkow, J., Zaleski, C., Jha, S., Batut, P., Chaisson, M., and Gingeras, T.R. (2013). STAR: ultrafast universal RNA-seq aligner. Bioinformatics *29*, 15-21.

Feitelson, D. (2003). Comparing Partitions with Spie Charts. Technical Report 2003-87 *School of Computer Science and Engigeering, The Hebrew University of Jerusalem*.

Giorgetti, A., Castano, J., Bueno, C., Diaz de la Guardia, R., Delgado, M., Bigas, A., Espinosa, L., and Menendez, P. (2017). Proinflammatory signals are insufficient to drive definitive hematopoietic specification of human HSCs in vitro. Exp Hematol *45*, 85-93 e82.

Giorgetti, A., Montserrat, N., Aasen, T., Gonzalez, F., Rodriguez-Piza, I., Vassena, R., Raya, A., Boue, S., Barrero, M.J., Corbella, B.A.*, et al.* (2009). Generation of induced pluripotent stem cells from human cord blood using OCT4 and SOX2. Cell Stem Cell *5*, 353-357.

Im, H., Grass, J.A., Johnson, K.D., Kim, S.I., Boyer, M.E., Imbalzano, A.N., Bieker, J.J., and Bresnick, E.H. (2005). Chromatin domain activation via GATA-1 utilization of a small subset of dispersed GATA motifs within a broad chromosomal region. Proc Natl Acad Sci U S A *102*, 17065-17070.

Krijthe, J.H. (2015). Rtsne: T-Distributed Stochastic Neighbor Embedding using a Barnes-Hut Implementation.

Kuleshov, M.V., Jones, M.R., Rouillard, A.D., Fernandez, N.F., Duan, Q., Wang, Z., Koplev, S., Jenkins, S.L., Jagodnik, K.M., Lachmann, A.*, et al.* (2016). Enrichr: a comprehensive gene set enrichment analysis web server 2016 update. Nucleic Acids Res *44*, W90-97.

Langmead, B., Trapnell, C., Pop, M., and Salzberg, S.L. (2009). Ultrafast and memory-efficient alignment of short DNA sequences to the human genome. Genome Biol *10*, R25.

Love, M.I., Huber, W., and Anders, S. (2014). Moderated estimation of fold change and dispersion for RNA-seq data with DESeq2. Genome Biol *15*, 550.

Machanick, P., and Bailey, T.L. (2011). MEME-ChIP: motif analysis of large DNA datasets. Bioinformatics *27*, 1696-1697.

Peres, J., Kwesi-Maliepaard, E.M., Rambow, F., Larue, L., and Prince, S. (2017). The tumour suppressor, miR-137, inhibits malignant melanoma migration by targetting the TBX3 transcription factor. Cancer Lett *405*, 111-119.

Picelli, S., Faridani, O.R., Bjorklund, A.K., Winberg, G., Sagasser, S., and Sandberg, R. (2014). Full-length RNA-seq from single cells using Smart-seq2. Nat Protoc *9*, 171-181.

Ramos-Mejia, V., Navarro-Montero, O., Ayllon, V., Bueno, C., Romero, T., Real, P.J., and Menendez, P. (2014). HOXA9 promotes hematopoietic commitment of human embryonic stem cells. Blood *124*, 3065-3075.

Ran, F.A., Hsu, P.D., Wright, J., Agarwala, V., Scott, D.A., and Zhang, F. (2013). Genome engineering using the CRISPR-Cas9 system. Nat Protoc *8*, 2281-2308.

Sturgeon, C.M., Ditadi, A., Awong, G., Kennedy, M., and Keller, G. (2014). Wnt signaling controls the specification of definitive and primitive hematopoiesis from human pluripotent stem cells. Nat Biotechnol *32*, 554-561.

Tyner, C., Barber, G.P., Casper, J., Clawson, H., Diekhans, M., Eisenhart, C., Fischer, C.M., Gibson, D., Gonzalez, J.N., Guruvadoo, L.*, et al.* (2017). The UCSC Genome Browser database: 2017 update. Nucleic Acids Res *45*, D626-D634.

Venables, W.N., and Ripley, B.D. (2002). Modern Applied Statistics with S (Springer-Verlag New York).

Zhang, Y., Liu, T., Meyer, C.A., Eeckhoute, J., Johnson, D.S., Bernstein, B.E., Nusbaum, C., Myers, R.M., Brown, M., Li, W.*, et al.* (2008). Model-based analysis of ChIP-Seq (MACS). Genome Biol *9*, R137.
