# Supplementary material for: GATA2 Promotes Hematopoietic Development and Represses Cardiac Differentiation of Human Mesoderm
Source: Stem Cell Reports. 2019 Aug 8;13(3):515–29. doi: 10.1016/j.stemcr.2019.07.009 (PMC6742600; doi:10.1016/j.stemcr.2019.07.009)
Supplement: Document S2. Article plus Supplemental Information [file mmc8.pdf]

Stem Cell Reports
Article

ISSCR

OPEN ACCESS

GATA2 Promotes Hematopoietic Development and Represses Cardiac Differentiation of Human Mesoderm

Julio Castaño,[1,2] Sergi Aranda,[3] Clara Bueno,[4] Fernando J. Calero-Nieto,[5] Eva Mejia-Ramirez,[1] Jose Luis Mosquera,[6] Enrique Blanco,[3] Xiaonan Wang,[5] Cristina Prieto,[4] Lorea Zabaleta,[7] Elisabetta Mereu,[8] Meritxell Rovira,[1] Senda Jiménez-Delgado,[1] Daniel R. Matson,[9] Holger Heyn,[8,10] Emery H. Bresnick,[9] Berthold Göttgens,[4] Luciano Di Croce,[3,11] Pablo Menendez,[4,11,12] Angel Raya,[1,2,11] and Alessandra Giorgetti[1,*]

[1]Center of Regenerative Medicine in Barcelona (CMRB), Hospital Duran i Reynals, Gran Via de L'Hospitalet, 199-203, Hospitalet de Llobregat, Barcelona 08908, Spain
[2]Center for Networked Biomedical Research on Bioengineering, Biomaterials, and Nanomedicine (CIBER-BBN), Madrid 28029, Spain
[3]Center for Genomic Regulation (CRG), The Barcelona Institute of Science and Technology, Universitat Pompeu Fabra, Barcelona 08003, Spain
[4]Josep Carreras Leukemia Research Institute and Department of Biomedicine, School of Medicine, University of Barcelona, Barcelona 08036, Spain
[5]Department of Hematology, Wellcome and MRC Cambridge Stem Cell Institute and Cambridge Institute for Medical Research, University of Cambridge, Cambridge, UK
[6]Bioinformatics Unit, Bellvitge Biomedical Research Institute (IDIBELL), Barcelona, 08908 Spain
[7]Laboratory of Hematological Diseases, Fundación Inbiomed, San Sebastian, 20009, Spain
[8]CNAG-CRG, Center for Genomic Regulation (CRG), Barcelona Institute of Science and Technology (BIST), Barcelona, Spain
[9]Department of Cell and Regenerative Biology, UW-Madison Blood Research Program, Carbone Cancer Center, University of Wisconsin School of Medicine and Public Health, Madison, WI 53705, USA
[10]Universitat Pompeu Fabra, Barcelona, Spain
[11]Institució Catalana de Recerca i Estudis Avançats (ICREA), Barcelona 08010, Spain
[12]Centro de Investigación Biomedica en Red en Cancer (CIBERONIC) ISCIII, Barcelona, Spain
*Correspondence: agiorgetti@cmrb.eu
https://doi.org/10.1016/j.stemcr.2019.07.009

SUMMARY

In vertebrates, GATA2 is a master regulator of hematopoiesis and is expressed throughout embryo development and in adult life. Although the essential role of GATA2 in mouse hematopoiesis is well established, its involvement during early human hematopoietic development is not clear. By combining time-controlled overexpression of *GATA2* with genetic knockout experiments, we found that GATA2, at the mesoderm specification stage, promotes the generation of hemogenic endothelial progenitors and their further differentiation to hematopoietic progenitor cells, and negatively regulates cardiac differentiation. Surprisingly, genome-wide transcriptional and chromatin immunoprecipitation analysis showed that GATA2 bound to regulatory regions, and repressed the expression of cardiac development-related genes. Moreover, genes important for hematopoietic differentiation were upregulated by GATA2 in a mostly indirect manner. Collectively, our data reveal a hitherto unrecognized role of GATA2 as a repressor of cardiac fates, and highlight the importance of coordinating the specification and repression of alternative cell fates.

INTRODUCTION

During embryonic development, hematopoietic stem cells (HSCs) emerge from hemogenic endothelium in the ventral wall of the aorta-gonad-mesonephros (AGM) region (Dzierzak and Speck, 2008; Ivanovs et al., 2011, 2014), and their specification is tightly orchestrated by temporal changes in the expression of master regulators during endothelial-to-hematopoietic transition (EHT). While animal models have been crucial in identifying several master regulators, such as GATA2, RUNX1, and TAL1 (Robert-Moreno et al., 2005; Wilson et al., 2010a, 2010b), how these factors drive human HSC emergence during EHT remains poorly understood.

GATA2 belongs to an evolutionarily conserved family of zinc finger transcription factors comprising six members: GATA1 to GATA6 (Merika and Orkin, 1993; Molkentin, 2000). GATA2, together with GATA1 and GATA3, are categorized as "hematopoietic" GATA factors and regulate the development of diverse hematopoietic lineages (Bresnick et al., 2012; Johnson et al., 2012; Katsumura et al., 2017; Orkin, 1992). The importance of GATA2 in HSC specification was first highlighted by gene targeting studies, because ablation of *Gata2* is embryonic lethal at embryonic day (E) 10.5 due to the collapse of primitive and definitive hematopoiesis (Gao et al., 2013; Ling et al., 2004; Tsai and Orkin, 1997). Notably, analysis of chimeric embryos generated with *Gata2*-null embryonic stem cells (ESCs) indicated that these cells failed to contribute to any hematopoietic lineage (Tsai et al., 1994). Likewise, mouse *Gata2*-null endothelial cells failed to produce HSCs because of impaired EHT (de Pater et al., 2013; Gao et al., 2013; Johnson et al., 2012; Lim et al., 2012). A primary role of GATA2 in promoting EHT has been recently demonstrated in humans (Gomes et al., 2018; Kang et al., 2018; Zhou et al., 2019).

This is an open access article under the CC BY license (http://creativecommons.org/licenses/by/4.0/).

In adult hematopoiesis, GATA2 is expressed at high levels in HSCs, early hematopoietic progenitors, and in erythroid/megakaryocyte lineages (Vicente et al., 2012). Recent studies showed that *GATA2* haploinsufficiency is associated with some familial cases of myelodysplastic syndrome, bone marrow failure, immunodeficiency, and MonoMAc syndrome (Dickinson et al., 2011; Hahn et al., 2011; Wlodarski et al., 2016), further supporting its important role in HSCs. Conversely, enforced expression of *GATA2* in cord blood-derived HSCs confers increased quiescence, an important hallmark of HSCs (Tipping et al., 2009).

We sought to explore the role of GATA2 during human hematopoietic development by inducing *GATA2* expression in differentiating human induced pluripotent stem cells (hiPSCs) (Takahashi et al., 2007). We show that *GATA2* induction during mesoderm patterning robustly promotes the generation of hemogenic endothelial progenitors (HEPs), and their further differentiation into hematopoietic progenitor cells (HPCs). Global transcriptome analysis and chromatin immunoprecipitation sequencing (ChIP-seq) combined with DNA massive sequencing revealed that GATA2 directly represses genes that promote cardiac cell fate differentiation and activates master hematopoietic regulators via direct and indirect mechanisms. Remarkably, *GATA2* knockout impaired hematopoietic development and enhanced cardiac potential of mesodermal progenitors.

RESULTS

GATA2 Promotes Robust Hematopoietic Differentiation

To analyze the impact of GATA2 in early human hematopoiesis, we first examined endogenous GATA2 expression in hiPSCs induced to form embryoid bodies (EBs) in serum-free medium with the successive addition of BMP4 (days 0–3), CHIR92001 (days 2–3), and hematopoietic cytokines (days 3–15) (Figure 1A). This protocol promotes mesoderm induction (days 2–3), specification of mesodermal cells to bipotential hemato-endothelial progenitors ($CD31^{+}CD34^{+}CD43^{-}CD45^{-}$; days 3–10) that can originate both endothelial and hematopoietic cells and could be considered equivalent to HEPs (Ayllon et al., 2015), and further commitment of HEPs to definitive HPCs ($CD34^{+}CD43^{+}CD45^{+}$; days 10–15) (Giorgetti et al., 2017; Sturgeon et al., 2014). *GATA2* was initially expressed at day 2 (Figure 1B), at the onset of mesoderm formation marked by the expression of *T* and *MIXL1* (Figure 1C). Its expression then progressively increased along with the emergence of HEPs and HPCs, in parallel with the master hemogenic regulators *RUNX1* and *SCL* (Figure 1B).

We next established transgenic hiPSCs in which the expression of transgenic *GATA2* could be temporally controlled by doxycycline (Dox) administration (hereafter termed iGATA2-hiPSCs) (Figure S1A). Robust transgenic overexpression of *GATA2* was confirmed in four clones (CL6, CL9, CL201, CL204) derived from two independent iGATA2-hiPSC lines by western blotting after 2 days of Dox treatment (Figure S1B). qRT-PCR analysis and *in vivo* functional assays showed that iGATA2-hiPSCs retained the expression of pluripotency markers and also the capacity to generate teratomas (Figure S1C).

Then, considering the expression of endogenous *GATA2*, we induced *GATA2* expression from day 2 to 7 in EBs generated from iGATA2-hiPSCs (Figures 1A and S1D–S1G). Flow cytometry analysis showed that enforced expression of *GATA2* significantly enhanced the production of HEPs (~2.5-fold increase of $CD31^{+}CD34^{+}CD45^{-}$ cells and ~2-fold increase of $CD34^{+}CD43^{-}CD45^{-}$ cells) in EBs at day 10 (Figures 1D and 1E), and promoted the generation of HPCs (~5-fold increase of $CD34^{+}CD43^{+}CD45^{+}$ cells) at day 15 (Figures 1D and 1E).

We used colony-forming unit (CFU) assays to confirm that GATA2 overexpression promotes hematopoiesis from iGATA2-hiPSCs. Dox treatment (days 2–7) significantly increased the total number of hematopoietic CFCs in day 10 EBs (Figure 2A). Notably, CFU scoring revealed an enhancement in all types of hematopoietic colonies (Figure 2A), suggesting that GATA2 expression promotes hematopoietic commitment by inducing mesodermal specification to HEPs at very early stages.

To better understand the role of GATA2 in early hematopoiesis, we treated iGATA2-hiPSCs with Dox at distinct stages of EB development (days 2–7, 7–15, and 10–15). Induction of GATA2 at days 2–7 had the greatest effect on HEP and HPC generation (Figure 2B), while treatment at days 7–15 had no significant effects on the HEP population, and only a small effect was observed for early HPCs. By contrast, GATA2 overexpression at days 10–15 led to a significant decrease in HPC numbers (Figure 2B), consistent with previous findings showing that high GATA2 expression in HSCs blocks normal hematopoiesis (Persons et al., 1999; Tipping et al., 2009).

To address whether the higher hematopoietic output could be a consequence of a higher HEP generation, $CD34^{+}$ cells were purified from day 10 EBs with or without Dox treatment, and were cultured on OP9 stromal cells (Choi et al., 2009; Ramos-Mejia et al., 2014) (Figure 2C, upper panel). After 4 days of coculture, both $CD34^{+}CD45^{+}$ and total $CD45^{+}$ cell subpopulations increased significantly in the cocultures derived from Dox-treated cells (~3- and 4-fold, respectively), whereas the number of HEPs decreased slightly (Figure 2C, lower panel). To further characterize the effect of GATA2 during mesoderm patterning, we

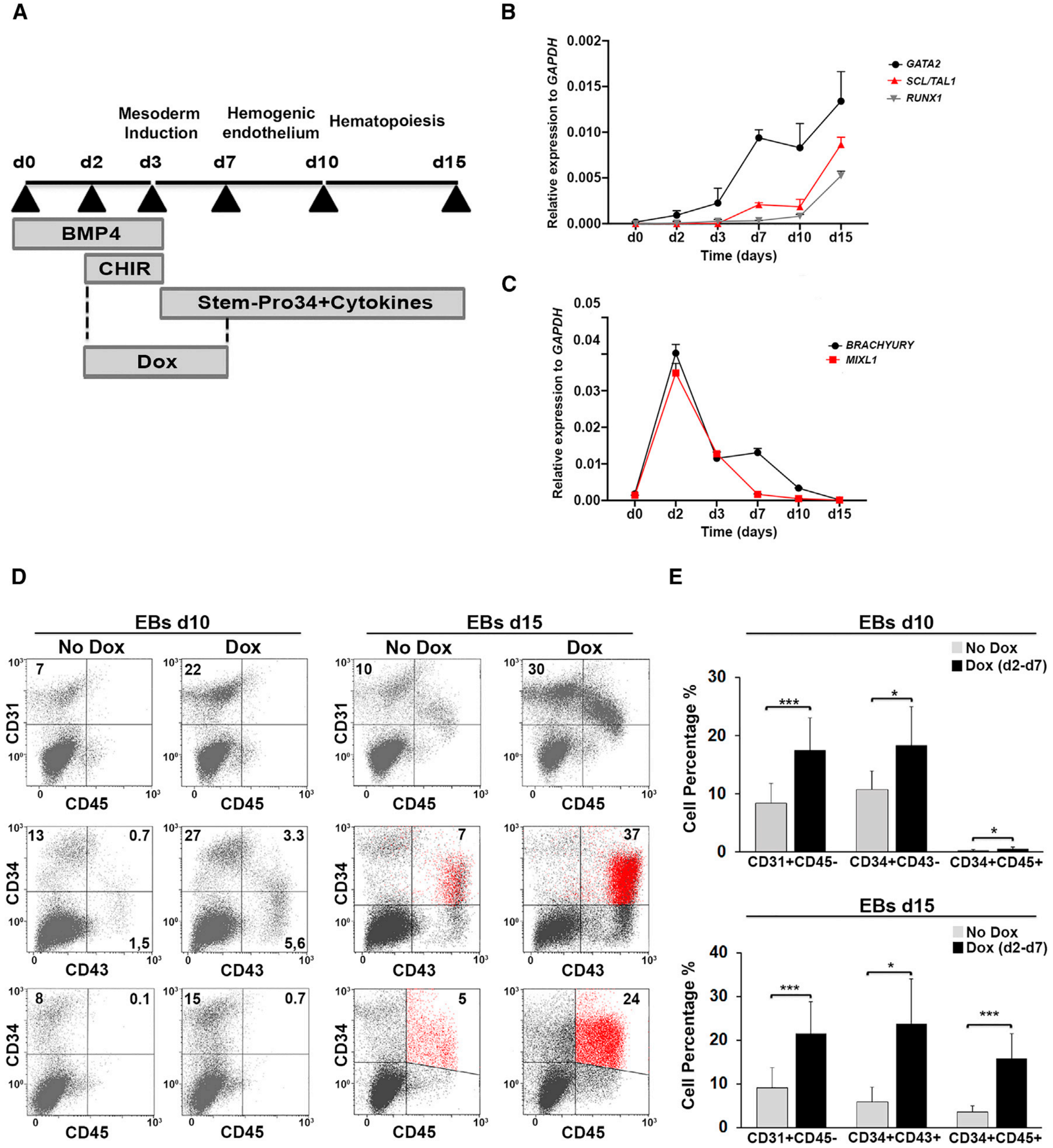

Figure 1. Early GATA2 Induction Enhances Hematopoietic Development from hiPSCs

(A) hiPSC hematopoietic differentiation based on EB generation.

(B) Time course of endogenous *GATA2*, *SCL/TAL1*, and *RUNX1* expression during EB development, normalized to *GAPDH*.

(C) Time course of endogenous mesodermal marker expression (*BRACHYURY* and *MIXL1*) during EB development, normalized to *GAPDH*. In (B) and (C), data represent the mean ± SD of three independent experiments.

(D) Representative flow cytometry analysis of HEPs ($CD31^+CD34^+CD45^-$ and $CD34^+CD43^-CD45^-$) and HPCs ($CD34^+CD45^+$ and $CD43^+CD45^+$) in EBs at days 10 and 15 in control and Dox-treated cells.

(E) Quantitative summary of HEP and HPC analysis at days 10 and 15 of EB differentiation in control and Dox-treated cells. Data represent the mean ± SD of 10 independent experiments. *$p < 0.05$, ***$p < 0.001$.

performed single-cell cloning assays of mesodermal cells ($KDR^+CD34^-CD31^-$) using OP9 stromal cells and conditions that support hemato-endothelial differentiation. Dox treatment slightly increased the hematopoietic/endothelial ratio over control (no-Dox) (Figure S2A, upper table). We repeated single-cell clonal analysis of $CD31^+CD34^+CD43^-$

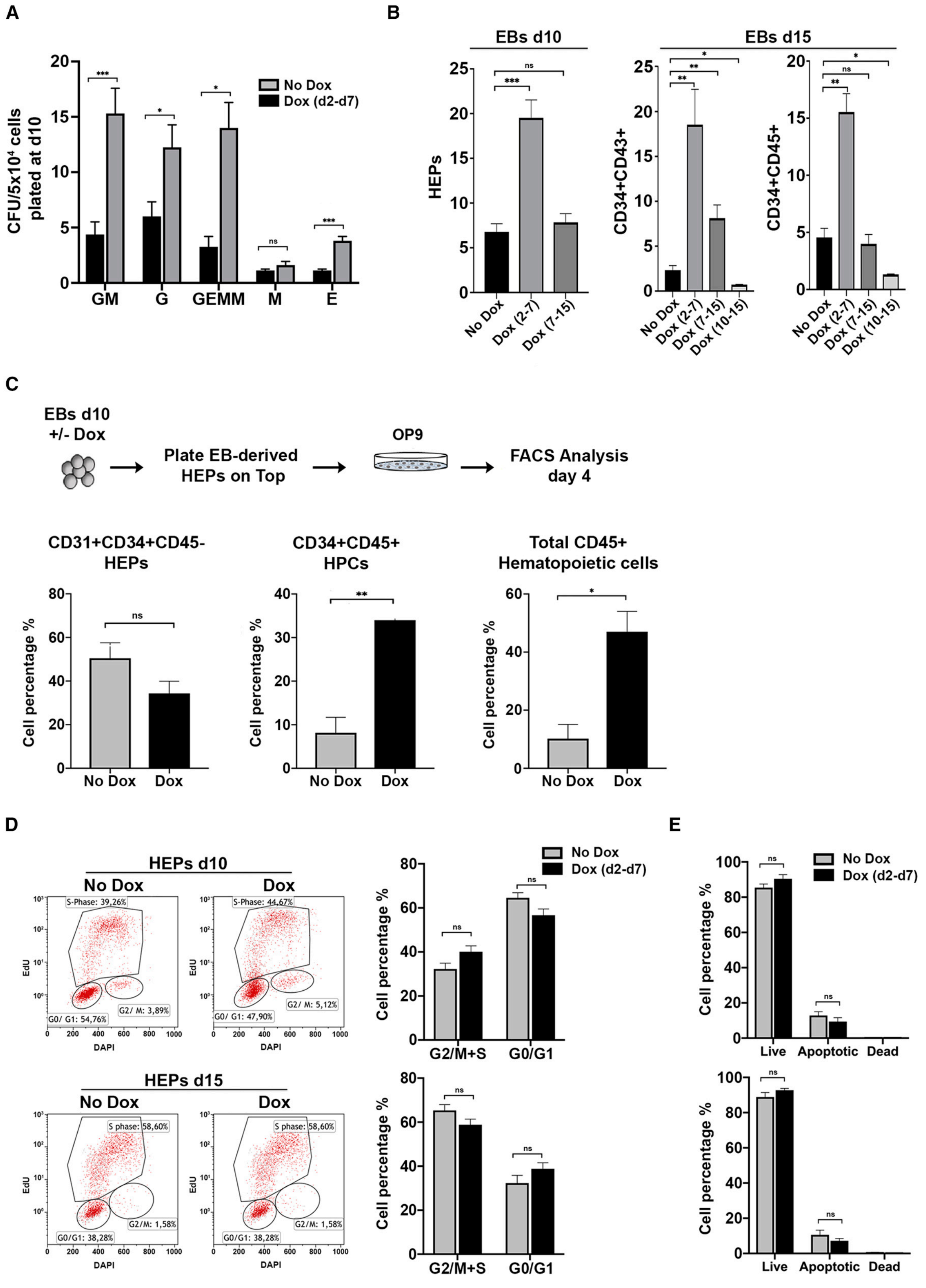

(legend on next page)

cells purified from day 7 EBs with or without Dox, finding that Dox-treated cells showed an increased number of hematopoietic colonies but a smaller proportion of endothelial growth (Figure S2A, lower table), overall increasing the hematopoietic/endothelial ratio by >4-fold.

Because GATA2 has been associated with adult HSC survival (Tipping et al., 2009), we questioned whether the increase in hematopoiesis was the consequence of GATA2-mediated proliferation/survival of emerging HEPs and HPCs. We analyzed apoptosis and cell-cycle distribution after GATA2 induction in HEPs and HPCs at days 10 and 15 of EB development, respectively, finding that GATA2 induction did not affect survival or proliferation of differentiating cells (Figures 2D, 2E, and S2B).

Overall, our data are consistent with recent reports (Kang et al., 2018; Zhou et al., 2019) showing that, rather than induction of HEP specification or the selective proliferation/survival of HEPs/HPCs, GATA2 induces hematopoietic development by promoting EHT.

GATA2 Activates the Hematopoietic Program and Inhibits Cardiac Genes

To gain mechanistic insight into how GATA2 promotes hematopoietic development, we performed RNA sequencing (RNA-seq) of fluorescence-activated cell sorting (FACS)-sorted control or GATA2-overexpressing HEPs from day 2 to 7 of differentiation. We used the criteria of >1.5-fold change and adjusted p value < 0.05 to identify differentially expressed genes (DEGs) in the two treatment groups.

Among 1,127 genes significantly deregulated by GATA2 induction, 700 were downregulated and 427 were upregulated (Table S2). Consistent with the *in vitro* results, GATA2 activated a broad spectrum of genes regulating HSC/HPC development (e.g., *RUNX1*, *MYB*, *STAT1*, *ITGA2B/CD41*, *SPN/CD43*, *SPI1/PU.1*, *ZBT3*, and *ALDH1A1*), as well as of genes of myeloid (*CD33*, *CD53*, *CD48*, *CSFR1*, and *MPO*) and erythroid (*NFE2*, *GATA1*, *KLF1*, *HBZ*, *HBE1*, *HBA1*, and *HBG2*) lineages (Figure S3A). Proinflammatory cytokines have been proposed as positive regulators of definitive hematopoiesis in the mouse AGM region and its zebrafish equivalent (Espin-Palazon et al., 2014; He et al., 2015; Sawamiphak et al., 2014). In accord with this, gene ontology (GO) and gene set enrichment analysis showed that GATA2-overexpressing HEPs were highly enriched for genes associated with immune response (*IRF7*, *IFI27*, *IFIT1*, *TMEM173*, *IFI6*, *IFITM1*, *TRIM6*, *TRIM14*, and *TRIM25*) (Figure S3A). Surprisingly, several highly significant GO categories of downregulated genes were related to heart development and cardiogenesis (Figure S3B), including transcription factors such as *TBX3*, *MYOCD*, *PTX2*, *NR2F2*, and *FOXC2*, and structural genes including *TNNC1*, *RYR3*, *SPNS2*, *DVL3*, *SMO*, *NEBL*, *HEG1*, and *CCM2L* (Figure S3B).

Furthermore, expression of genes related to angiogenesis and endothelial cell differentiation (*JAG-1*, *KDR*, *SOX17*, *PCDH12*, *TEK*, *ESM1*, and *SCUBE1*) were also found to be downregulated (Figure S3B). These data strongly suggest that GATA2 has a dual activity during mesodermal patterning.

GATA2 Directly Binds Cardiac Genes

To determine the direct effects of GATA2 on gene transcription, we performed ChIP-seq analysis for GATA2 occupancy on FACS-sorted GFP$^+$ cells at day 7 of EB development, coinciding with the maximum expression of the GATA2 transgene (Figure S1E). We identified 2,097 GATA2 binding-associated genes (Figures 3A, S3C, and S3D). GATA2 was found to be enriched around the transcriptional start site of many genes harboring a GATA2 binding motif (Figures 3B–3D and S3E), indicating that GATA2 does not occupy unscheduled genomic binding sites in iGATA2-hiPSCs. Integrated analysis with RNA-seq data indicated that only 8% of upregulated genes were decorated with GATA2 (35 genes in total), whereas up to 20% of downregulated genes were occupied by GATA2 (143) (Figure 3E). Probabilistic analysis indicated that GATA2 occupancy on repressed genes was significantly higher than would be expected by random chance ($p < 10^{-16}$), suggesting a predominant function of GATA2 repressing gene transcription during mesodermal lineage

Figure 2. GATA2 Induction Promotes Hemogenic Endothelium Transition

(A) CFU potential of day 10 EB progenitors in control and Dox-treated cells. Colonies were counted from each group after 2 weeks of culture and scored for the following morphological subsets: burst-forming unit-erythroid (E); CFU-granulocyte, macrophage (GM); CFU-granulocyte, erythroid, macrophage, megakaryocyte (GEMM); CFU-granulocyte (CFU-G); and CFU-macrophage (CFU-M). Data represent the mean ± SD of the total number of colonies per 50,000 cells seeded of 6 independent experiments.

(B) Quantitative summary of HEP and HPC analysis at days 10 and 15 of EB differentiation following stepwise treatment with Dox. Data represent the mean ± SD of 6 independent experiments.

(C) Schematic of sorted HEP cell differentiation using OP9 coculture (upper panel). GATA2-induced HEPs showed a higher capacity to differentiate into CD34$^+$/CD45$^+$ and CD45$^+$ hematopoietic cells (lower panel). Data represent the mean ± SD of 3 independent experiments.

(D) Representative dot plots and bar graphs show cell-cycle analysis using EdU and DAPI staining on HEPs and HPCs treated or not with Dox. Data represent the mean ± SD of 3 independent experiments.

(E) Apoptosis analysis in HEPs and HPCs in control and Dox-treated cells.

Data represent the mean ± SD of 3 independent experiments. *p < 0.05, **p < 0.01, ***p < 0.001.

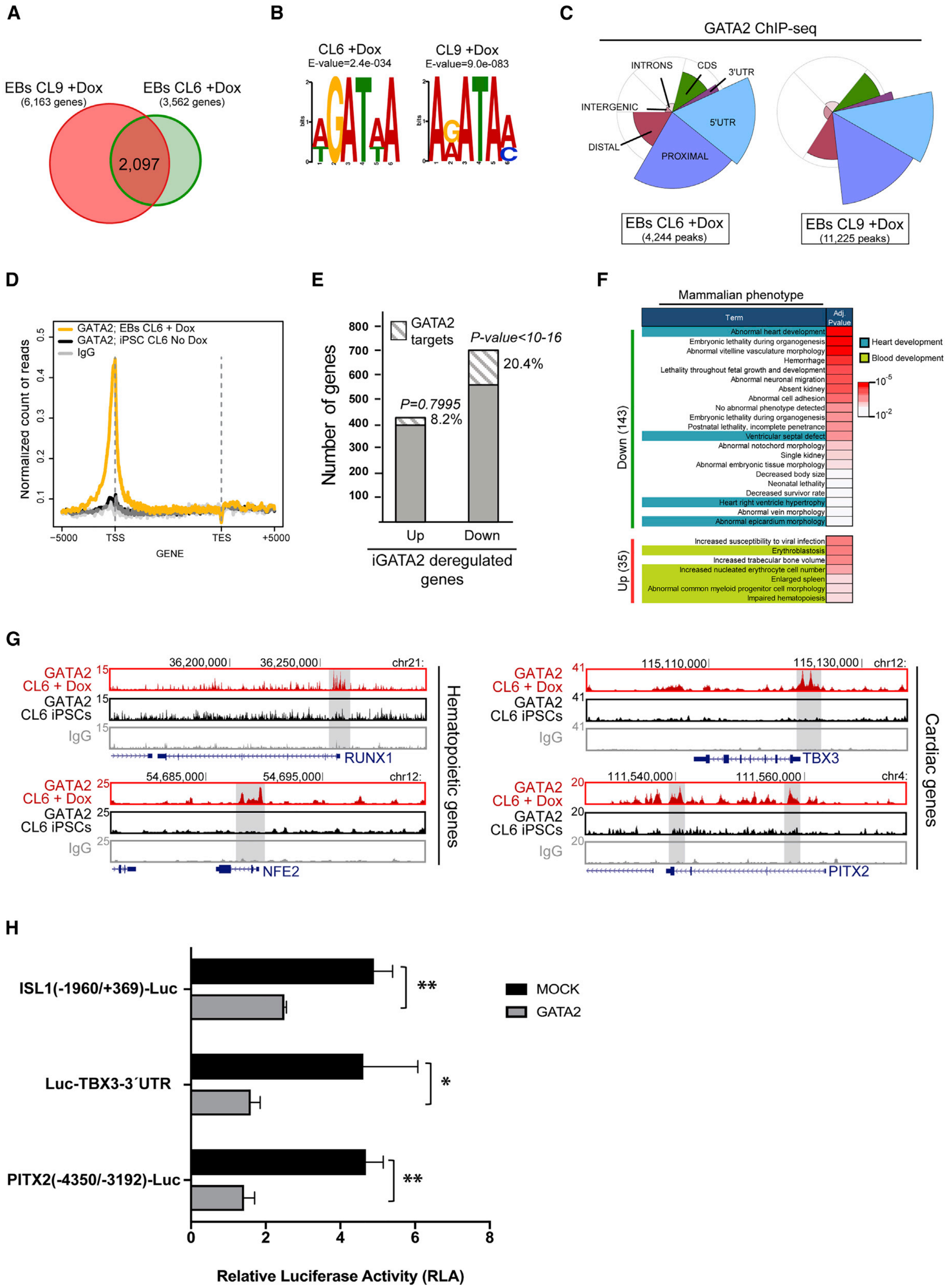

(legend on next page)

differentiation (Figure 3E). Among the upregulated GATA2 target genes, we identified determinants for hematopoietic development such as *RUNX1* and *NFE2* (Figures 3F and 3G). Of note, several of the downregulated GATA2 target genes were associated with heart development, such as *TBX3* and *PITX2* (Figures 3F and 3G), confirming the RNA-seq data.

The specific binding of GATA2 to proximal gene regions of cardiac regulators strongly suggests that GATA2 directly controls the repression of genes involved in cardiac development. To test this, we used a luciferase reporter assay in COS7 cells for three cardiac genes (*TBX3*, *PITX2*, and *ISL-1*), according to the ChIP-seq data. Luciferase activity driven by the 3′ UTR of the *TBX3* promoter, the −1,960/+369 region of the *ISL-1* promoter, and the −4,350/−3,192 region of the *PITX2* promoter, was significantly reduced by GATA2 coexpression (Figure 3H). Thus, in addition to validating GATA2 as a pro-hematopoietic fate regulator, these data point to an additional and undescribed role for GATA2 in repressing cardiac regulation.

Single-Cell RNA-Seq of Mesodermal and Hemato-Endothelial Progenitors

To better characterize the effect of GATA2 overexpression on mesodermal diversification, we performed single-cell RNA-seq (scRNA-seq) on FACS-purified mesodermal ($KDR^{+}CD34^{-}CD31^{-}$; EBs d5) and hemato-endothelial progenitor ($KDR^{+}CD34^{+}CD43^{-}$; EBs d7) cells with or without Dox (Figure S4A). EB-derived cells from day 2 were profiled as the starting population.

In unsupervised clustering based on the Seurat method (Butler et al., 2018), we identified five transcriptionally distinct cell clusters (Figure 4A). The t-distributed stochastic neighbor embedding (t-SNE) projection visualized that day 2 EB-derived cells formed one expression signature cluster, whereas mesodermal (day 5) and hemato-endothelial (day 7) cells showed the presence of two subpopulations in each cluster (Figure 4B). We assigned biological identities to each cluster based on the expression of key marker genes (adjusted p value < 0.05) (Table S3). Cluster 1 (EBs day 2) showed high expression of pluripotency genes (*NANOG*, *OCT4/POU5f1*, *ZFP42/REX1*, *DPPA4*, and *SALL2*), indicating maintenance of undifferentiated pluripotent cells. Cluster 2 (mesodermal S1) showed a multi-lineage mesodermal identity (*BMP4*, *MSX2*, *PDGFRA*, *LGR5*, *CDX2*, *FRZB*, *HOXA1*, *BMP5*, *FTH1*, *WNT5A*, *WNT5B*, *HAND1*, *TNNT1*, and *TBX3*). Cluster 3 (mesodermal S2) lacked expression of mesodermal markers, but co-expressed *ETV2*, its target SCL/*TAL1*, and *EGFL7*, which are responsible for restricting mesoderm specification to endothelium fate (Wareing et al., 2012), and genes suggestive of a mesenchymal phenotype (*COL1A1*, *COL6A3*, *COL3A1*, *COL6A2*, *ACTA2*, and *LUM*). Cluster 4 (hemato-endothelial S1) and cluster 5 (hemato-endothelial S2) shared the expression of several key hemato-endothelial markers (*CDH5*, *PECAM1*, *ICAM2*, *CD40*, *ESAM*, *FLI1*, *ERG*, *ETS1*, and *HHEX*); however, hemato-endothelial S2 cells showed a more restrictive endothelial identity (expressing selectively *NOTCH4*, *SOX7*, *NRP2*, *TEK*, and *NOS3*). Also, we noted that hemato-endothelial S1 was enriched for genes that regulate cell cycle (*AURKB*, *TOP2A*, *CDK1*, *MKI67*, *BRCA2*, *CASC5*, and *CDCA5*).

Next, we retrospectively colored each cluster on the basis of Dox and no-Dox conditions (Figure 4C). For each cluster,

Figure 3. Identification of GATA2 Target Genes during Mesoderm Specification

(A) Venn diagram indicating the overlap between the number of genes found targeted by GATA2 in each replicate of the ChIP-seq of GATA2 in EBs (plus Dox).

(B) MEME-ChIP motif analysis on the sequence of the GATA2 peaks in two independent ChIP-seq replicates showing an enrichment of GATA2 motifs at the center of the ChIP-seq peaks, as the top-ranked motif.

(C) Genomic distribution of ChIP-seq peaks of GATA2 compared with the whole genome in two independent clones. The pie chart represents the distribution of GATA2 peaks corrected by the genome-wide distribution of each gene feature (background circle distribution). The pie chart indicates that GATA2 preferentially occupies TSS neighborhood regions, including 5′ UTR and PROXIMAL regions. The DISTAL region is the region within 2.5 and 0.5 kbp upstream of the TSS. The PROXIMAL region is the region within 0.5 kbp of the TSS. CDS is the protein CoDing Sequence. INTRONS are intronic regions. INTERGENIC is the remainder of the genome. TSS is the transcription start site.

(D) Meta-gene plot showing the GATA2 ChIP-seq profile occupancy in iPSCs (CL6 no-Dox) and EBs (CL6 plus Dox) and IgG from −5 kb of the TSS until +5 kb of transcription end site (TES).

(E) Histogram of the proportion of GATA2 target genes deregulated after induction of GATA (iGATA2) (up or down, 1.5-fold change, false discovery rate < 0.05) during EB development (p value was calculated using chi-square test).

(F) Functional analysis on the mammalian phenotype enrichment ontology for the deregulated GATA2 target genes (MGI database).

(G) UCSC genome browser screenshots of several developmental hematopoietic and cardiac genes targeted by GATA2.

(H) Relative luciferase activity of COS7 cells transfected with reporter plasmids for *ISL1* (−1,960/+369), *PITX2* (−4,350/−3,192), or *TBX3* 3′ UTR genes and pWPI-GATA2 plasmid. Relative luciferase activity is shown as the ratio of luciferase activity to that in cells cotransfected with mock vector.

A representative result of three independent experiments performed in triplicate is shown as the mean ± SD. Differences were determined using Student's t test; *p < 0.05, **p < 0.01.

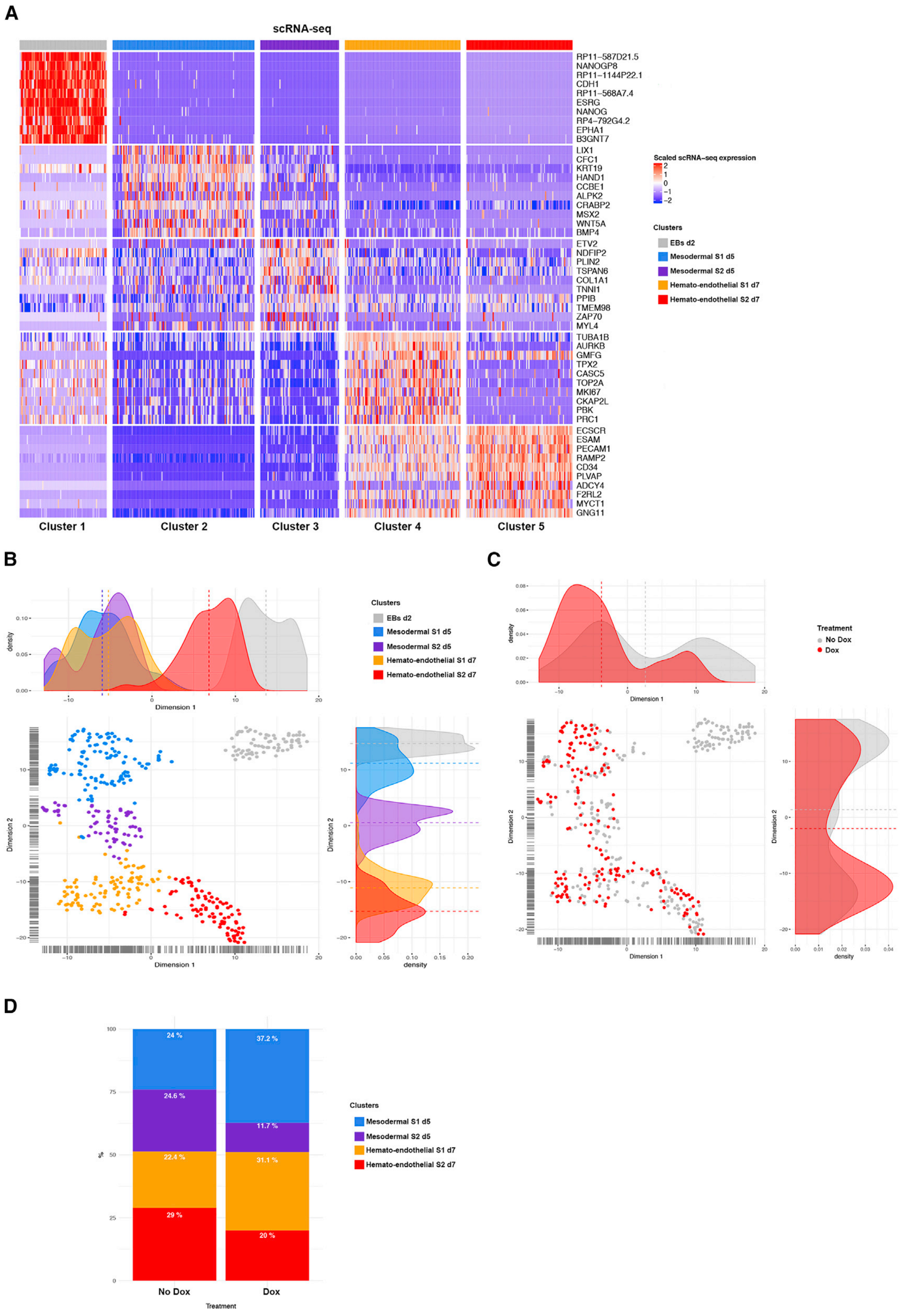

(legend on next page)

the number of cells per condition was calculated and each Dox condition was compared with that of the corresponding no-Dox condition (Table S3). A chi-square test showed that treatment and cluster populations were not independent ($p = 0.0002687$). Considering an adjusted p value < 0.05, the *post hoc* comparisons showed that, at day 5 of EB development, the proportion of mesodermal S1 cells increased significantly in Dox conditions (chi-square = 6.816191, adjusted p value = 0.018067005) (Figure 4D). At day 7, GATA2 overexpression induced an enrichment of the hemato-endothelial S1 cluster (chi-square = 3.082502, adjusted p value = 0.079138704) (Figure 4D).

Based on these data, we speculate that GATA2 enhances the proportion of cells with a multi-lineage mesodermal signature and further increases the probability, at the single-cell level, that a mesodermal progenitor acquired a hemato-endothelial transcriptional profile. These data are consistent with our FACS analysis (Figure S4A) and are in line with recent findings showing that GATA2 overexpression enhances the generation of mesodermal cells and further promotes EHT (Zhou et al., 2019).

Finally, we performed a new clustering analysis of mesodermal (day 5) and hemato-endothelial single cells (day 7) based on DEGs (1,127 genes) identified in our bulk RNA-seq analysis (HEPs day 10) (Table S4). Using t-SNE and hierarchical clustering to visualize the data, five main clusters were found (Figures S4B and S4C). GO enrichment analysis and over-representation analysis (Boyle et al., 2004) revealed that cluster 0 and cluster 4 were associated with hemato-endothelial progenitors (*ESCR*, *CDH5*, *ERG*, *SOX17*, and *HOXA9*), cluster 3 mesoderm (*TBX3* and *MSX1*), cluster 1 mesenchymal (*FN1*, *LAMA1*, and *COL5A1*), and cluster 2 epiblast (*EPCAM* and *CDH1*) (Figure S4D). These data are in line with the scRNA-seq analysis and suggest that most of the DEGs (1,056 genes) of day 10 were already differentially expressed in our single cells at d5 and d7.

GATA2 Knockout Inhibits Hematopoietic Development and Favors Cardiomyogenesis

Our results so far strongly suggest that transient GATA2 expression promotes hematopoietic differentiation and represses alternative mesodermal fates during HEP specification. To address whether GATA2 is necessary for specification of HEPs, we used CRISPR/Cas9 gene editing to target exon 2 of *GATA2* and generate knockout hiPSC-GATA2 clones (hiPSC-GATA2^{KO}). After expanding individual clones, we selected two targeted clones with biallelic mutations (CL14 and CL19; Figure 5A). Immunocytochemistry and qRT-PCR analysis confirmed the absence of GATA2 expression during hiPSC-GATA2^{KO} differentiation (Figures S5A and S5B). No predicted CRISPR off-targets were detected by genomic sequencing of hiPSC-GATA2^{KO} clones (Figure S5C). The hiPSC-GATA2^{KO} clones retained a normal karyotype and maintained the expression of pluripotency markers (Figures S5D and S5E), confirming that the *GATA2* knockout is compatible with human stem cell pluripotency.

We differentiated wild-type and hiPSC-GATA2^{KO} clones into hematopoietic cells as described earlier (see Figure 1A). As expected, *GATA2* knockout significantly affected HEP generation ($CD31^{+}CD34^{+}CD45^{-}$) in day 10 EBs (Figure 5B) and markedly decreased the number of HPCs ($CD34^{+}CD43^{+}CD45^{+}$) in day 15 EBs (Figure 5B). Moreover, we consistently failed to detect hematopoietic CFCs at day 10 of EB development (data not shown).

In line with the transcription and ChIP-seq data, qRT-PCR analysis revealed that the cardiac regulators *TBX3*, *NKX2.5*, and *MYOCD* were progressively upregulated during hiPSC-GATA2^{KO} EB development, whereas the hematopoietic transcription factors *SCL*, *GATA1*, and *PU.1* were markedly suppressed (Figure 5C).

As these observations strongly suggest that interfering with *GATA2* expression might be an effective strategy to generate cardiomyocytes *ex vivo*, we differentiated hiPSC-GATA2^{KO} and iGATA2-hiPSC lines (with or without Dox administration, days 2–7) to cardiomyocytes using a well-characterized protocol (Lian et al., 2013) (Figure 6A), and measured the expression of the cardiac structural proteins troponin I (cTnI) and myosin heavy chain (MHC) after 20 days. Remarkably, we found a ~3-fold increase in the number of $cTnI^{+}$ MHC^{+} cells in differentiated hiPSC-GATA2^{KO} cells compared with iGATA2-iPSC cells in the absence of Dox (Figures 6B and 6C). Conversely, Dox administration led to a significant decrease in the number of $cTnI^{+}$ MHC^{+} cells in the iGATA2-iPSC line (Figures 6B and 6C). As a functional readout of cardiomyocyte generation, we monitored for the appearance of beating cells. Whereas the majority of hiPSC-GATA2^{KO} cells started beating at days 8–10 of differentiation and persisted throughout the 20-day experiment (Videos S1 and S2),

Figure 4. Single-Cell RNA-Seq Analysis Reveals GATA2 as a Driver of Hemato-Endothelial Specification
(A) Heatmap showing the expression pattern of top 10 gene markers distinguishing 5 clusters from scRNA-seq analysis. Colored top bar indicates assigned cluster. Red indicates the highest scaled expression and blue the lowest.
(B) t-SNE visualization plot of day 2 (d2) EBs, mesodermal d5 and hemato-endothelial d7 cells according to clusters in (A).
(C) t-SNE visualization plot of single cells of each cluster retrospectively colored by Dox (red) and no-Dox treatment (gray).
(D) Stacked bar diagram showing the percentage of cells in each cluster with respect to treatment conditions.

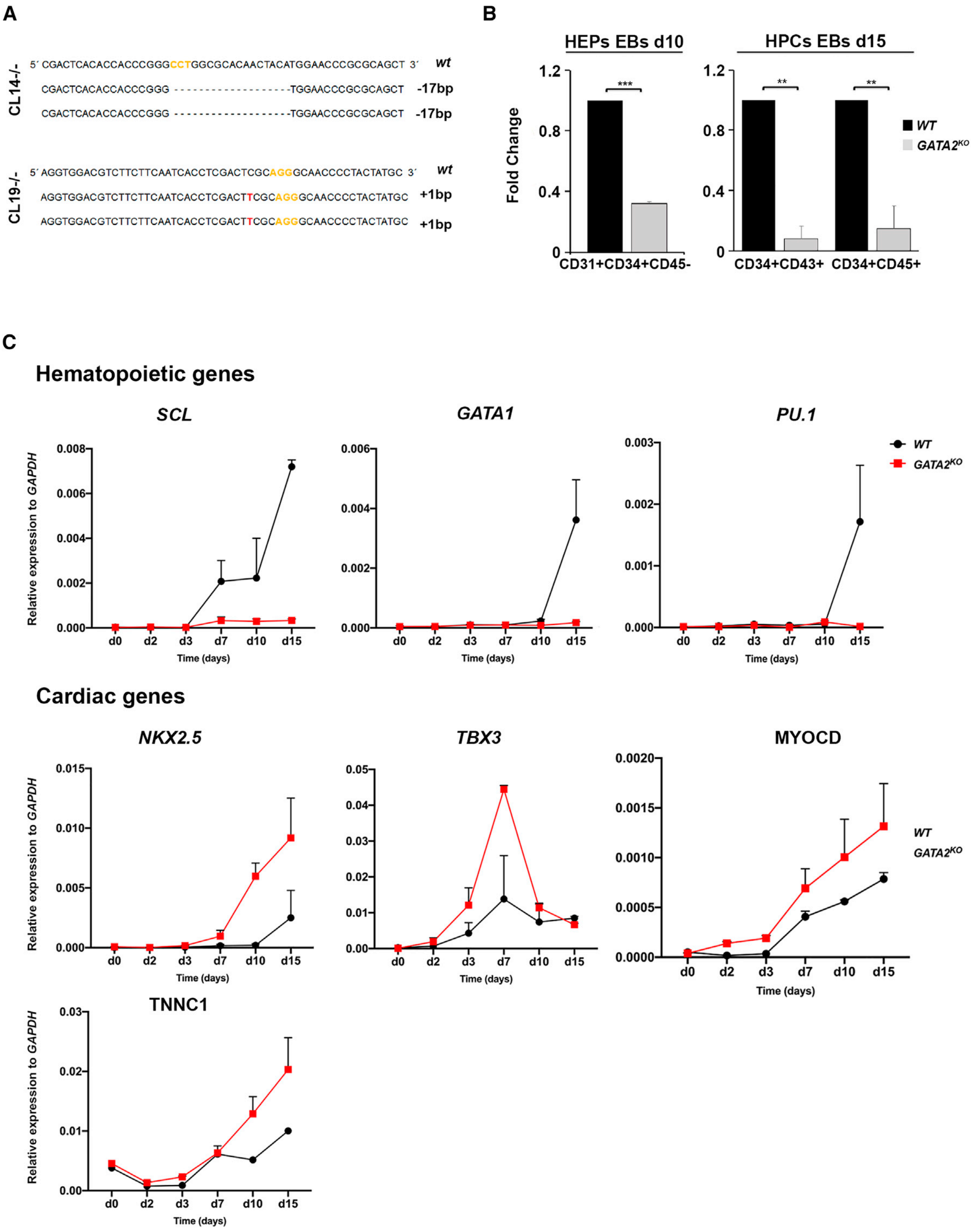

Figure 5. GATA2 Is Essential for HPC Development

(A) Representative sequencing of targeted homozygous hiPSC clones at the *GATA2* locus using sgRNA1 and sgRNA2; PAM sequences are labeled in yellow.

(B) Quantitative summary of HEP and HPC FACS analysis at d10 and d15 of EB development in wild-type (WT) and hiPSC-GATA2^{KO} clones. Data represent the mean ± SD fold change relative to *WT* iPSC lines.

(C) qRT-PCR analysis of hematopoietic and cardiac regulators in WT and hiPSC-GATA^{KO} clones during EB development.

Data represent the mean ± SD of 3 independent experiments. $^{**}p < 0.01$, $^{***}p < 0.001$.

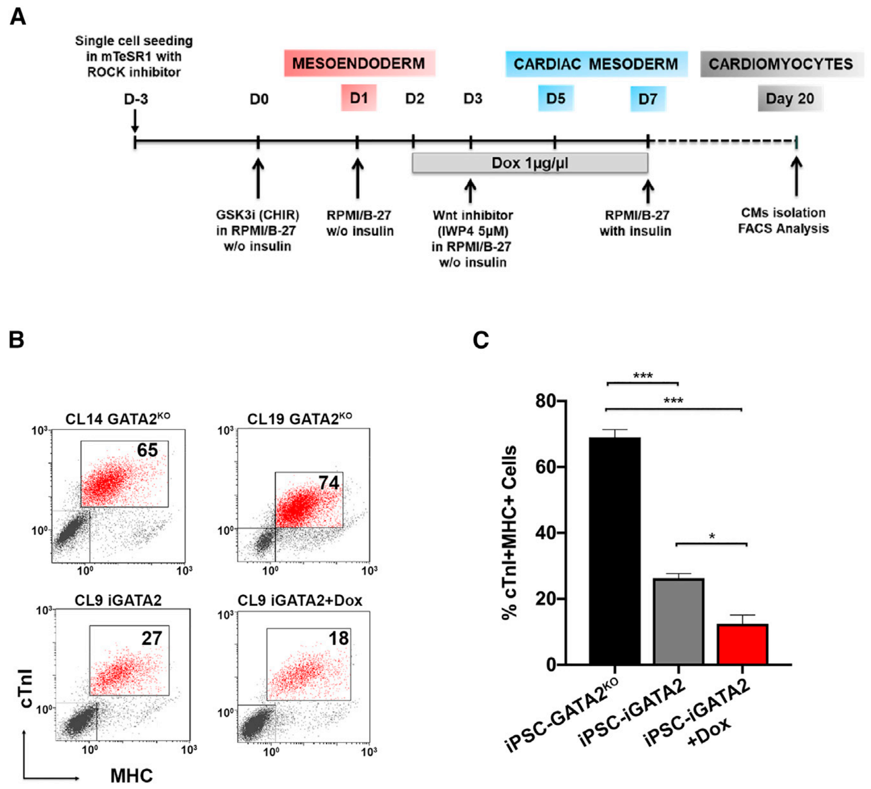

Figure 6. GATA2 Knockout Promotes Cardiomyocyte Differentiation

(A) Schematic of the cardiomyocyte differentiation protocol.

(B) Representative FACS analysis of the percentage of cTnI$^+$ MHC$^+$ cells in hiPSC-GATA2^{KO} and iGATA2-iPSC lines (with and without Dox treatment) at day 20 of differentiation.

(C) Bar graph showing the mean percentage of cTnI$^+$ MHC$^+$ cells in (B). Data represent the mean ± SD of 3 independent experiments. *$p < 0.05$, ***$p < 0.001$.

very few iGATA2-iPSC cells treated with Dox were beating (Video S3). Taken together, these results suggest that GATA2 is both an activator of hematopoiesis and a repressor of cardiac cell fate.

DISCUSSION

GATA2 has long been implicated as a master regulator of murine hematopoiesis (Tsai et al., 1994; Tsai and Orkin, 1997), and its dysregulation is associated with human immunodeficiency syndromes (Spinner et al., 2014). However, its role in early human hematopoiesis is less clear. Human iPSCs are a useful model to interrogate the molecular mechanisms driving early hematopoietic development.

To our knowledge, this is the first study showing a dual activity of GATA2 in human early hematopoiesis. We show that GATA2 drives mesoderm progenitors to differentiate into blood cells and represses cardiac fates. Indeed, our *GATA2* knockout model confirmed that loss of GATA2 activity impairs hematopoietic development and enhances cardiomyocyte differentiation.

Earlier studies both *in vivo* and in stem cell models suggested that hematopoietic and cardiac lineages develop in close proximity and are mutually antagonistic (Bussmann et al., 2007; Chagraoui et al., 2018; Chan et al., 2013; Freire et al., 2017; Kouskoff et al., 2005; Liu et al., 2013; Schoenebeck et al., 2007; Van Handel et al., 2012). For example, overexpression of *Scl* in mesodermal cells promotes hematopoietic development at the expense of cardiomyogenesis in differentiated mouse ESCs (Ismailoglu et al., 2008), whereas *Scl*-deficient mice show ectopic cardiomyocytes in yolk salc endothelium and die at E9.5 due to the complete absence of hematopoiesis (Shivdasani et al., 1995; Van Handel et al., 2012). Similarly, *Etv2/Er71* deficiency leads to a complete block of hemato-endothelial development and a concomitant expansion of the cardiac lineage in mutant embryos (Koyano-Nakagawa and Garry, 2017; Lee et al., 2008; Liu et al., 2012; Rasmussen et al., 2011), whereas *Etv2* overexpression in differentiating mouse ESCs shows the opposite phenotype (Liu et al., 2012).

To date, a direct role for GATA2 in cardiac development has not been demonstrated. *Gata2*-null mice have no apparent cardiovascular phenotype, but to our knowledge studies specifically addressing heart malformation in *Gata2* mutants have not been performed. Whether GATA2 overexpression *in vivo* enhances hematopoietic cell specification while concurrently retarding cardiac development should be addressed in the future.

GATA2 is known to cooperate with ER71/ETV2 and SCL/TAL1 to regulate endothelial and hematopoietic programs in stem cells (Elcheva et al., 2014; Shi et al., 2014).

The recent observation that coexpression of ER71/ETV2, SCL/TAL1, and GATA2 during mouse ESC differentiation enhances FLK-1^{+} hemangioblast production while blocking cardiac output (Liu et al., 2013) suggests a critical regulatory relationship between these factors during mesoderm diversification. Nevertheless, the gene regulatory network governing hematopoietic and cardiac development is poorly understood. Our bulk RNA-seq data revealed that GATA2 overexpression failed to upregulate ER71/ETV2 expression in our HEP model, which is consistent with the finding that ER71 is expressed before GATA2 during both early mouse and ESC development (Liu et al., 2013). Based on recent studies (Chagraoui et al., 2018; Org et al., 2015), it is reasonable to presume that GATA2 and SCL collaborate to promote blood specification at the expense of cardiac fates. Yet, our deep DNA motif enrichment analysis of GATA2 targets demonstrate the absence of SCL binding sites (CANNTG) in both GATA2-bound activated and repressed genes, which suggests that these two factors—at least in our experimental model—act independently. Of note, recent analysis at the single-cell level revealed that only a subset of mouse E8.5 *Scl*$^{-/-}$ endothelial cells upregulated expression of a few cardiac-related genes. However, those cells did not display a full cardiomyocyte transcriptional program and continued to express key endothelial markers (Pijuan-Sala et al., 2019; Scialdone et al., 2016). Therefore, the role of SCL in the specification of hematopoietic fate and as a cadiac repressor needs to be determined.

ChIP-seq indicated that GATA2 acts more as a repressor than an activator during mesodermal diversification. Specifically, GATA2 binds directly to cardiac regulator promoters, leading to their downregulation. Although previous studies in HPCs and mature blood cells have shown that endogenous GATA2 preferentially occupies sites distant to promoters (Calero-Nieto et al., 2014; Fujiwara et al., 2009; Huang et al., 2016; Wilson et al., 2015), our analysis revealed GATA2 binding promoter regions. Whether this apparent discrepancy is a consequence of cell-type-specific differences in GATA2 occupancy, the fact that GATA2 is overexpressed in our studies, or both, is unclear.

Enforced expression of instructive factors is an accepted strategy to guide lineage fate commitment (Doulatov et al., 2013; Elcheva et al., 2014; Nakajima-Takagi et al., 2013; Navarro-Montero et al., 2017; Ramos-Mejia et al., 2014; Sugimura et al., 2017), and offers the possibility to generate any differentiated cell type from hPSCs. Our study provides cellular and molecular evidence that GATA2 induction promotes an enhancement of mesodermal cells with hemato-endothelial potential, and decreases the probability that alternative mesodermal fates occur. Accordingly, GATA2 could be a target for manipulation to improve the yield of target cells (blood or cardiomyocytes) from hPSCs for drug screening and disease modeling.

In summary, we establish a novel role for GATA2 during mesodermal lineage specification and provide new insights into the complex regulatory network that controls human early hematopoietic development.

EXPERIMENTAL PROCEDURES

Human iPSC Culture

Human GATA2-hiPSC and iPSC-GATA2^{KO} lines were maintained on Matrigel-coated 60-mm plates in mTESR1 medium (Giorgetti et al., 2017). Culture medium was changed daily and cells were passaged weekly by EDTA dissociation.

EB-Based Hematopoietic Differentiation

Human iPSCs were differentiated as described (Giorgetti et al., 2017). In brief, EBs were treated with the GSK3 inhibitor CHIR99021 (3 μM) from day 2 to 3 of culture (Sturgeon et al., 2014). From day 3, EBs were cultured in differentiation medium until day 15. To induce transgene GATA2 expression Dox (1 μg/mL) was added from day 2 to 7 of differentiation.

Cardiac Differentiation

Human iPSCs were differentiated in monolayer cultures with modulators of canonical Wnt signaling (Lian et al., 2013). Contracting cardiomyocytes could be observed between day 8 and 10 of differentiation. Differentiated cells were disaggregated at day 20 with 0.25% trypsin-EDTA for 5–8 min at 37°C for FACS analysis.

ChIP-Seq Combined with DNA Massive Sequencing

GATA2-iPSCs (CL6 and CL9) were differentiated in the presence of Dox (from day 2 to 7). ChIP experiments were performed with $\sim 1.5 \times 10^6$ cells using the ChIP-IT High Sensitivity Kit from Active Motif (no. 53040), and specific antibodies for GATA2 (Santa Cruz; sc-9008) or rabbit IgG as isotype control (Abcam; 172730), Libraries were prepared according to Illumina instructions and sequenced using the HiSeq 2000 platform (Illumina).

Statistical Analysis

All data are expressed as mean ± SD. Statistical comparisons were performed using Student's t test (95% confidence interval). Statistical significance was defined as $p < 0.05$.

ACCESSION NUMBERS

The accession number for the ChiP-seq data reported in this paper is GEO: GSE107639; the accession number for the single-cell RNA-seq data is GEO: GSE133996; and the accession number for the RNA-seq data is GEO: GSE118980.

SUPPLEMENTAL INFORMATION

Supplemental Information can be found online at https://doi.org/10.1016/j.stemcr.2019.07.009.

AUTHOR CONTRIBUTIONS

Contribution, J.C. conceived the study, designed and performed the experiments, analyzed the data, and wrote the manuscript. S.A., F.J.C.-N., J.L.M., E.M.-R., C.B., E.B., X.W., C.P., L.Z., S.J.-D., E.M., D.R.M., and M.R. performed the experiments and analyzed the data. H.H., E.H.B., B.G., L.d.C., P.M., and A.R. analyzed the data and interpreted the results. A.G. conceived the study, designed and performed experiments, analyzed data, and wrote the manuscript.

ACKNOWLEDGMENTS

The authors thank Dr Catherine Porcher (University of Oxford) for the SCL antibody, Olalla Iglesias, Anna Duarri, and Yvonne Richaud for help with cell differentiation, and core of CMRB for technical assistance. A.G. is supported by Ramón y Cajal Program (RyC-2013-13221) and MINECO (SAF2016-80205-R). C.B. is supported by the FIS/ISCIII (PI14/01191), Spanish Cancer Association (CI15152720BUEN). J.L.M. is supported by ISCIII (CA18/00045) and by the European Social Fund (ESF). M.R. is supported by MINECO (SAF2015-73226-JIN). P.M. is funded by the PERIS Program of the Generalitat de Catalunya and by the Obra Social la Caixa-Fundacion Josep Carreras. H.H. is supported by Miguel Servet (CP14/00229). CNAG-CRG Core funding is from the ISCIII, and the Generalitat de Catalunya. Gottgens laboratory is supported by The Wellcome Trust, MRC, CRUK, NIH-NIDDK, and core support grants by The Wellcome Trust to the Wellcome and MRC Cambridge Stem Cell Institute. Additional funding came from Generalitat de Catalunya (2014-SGR-1460 and PERIS SLT002/16/00234); MINECO (SAF2015-69706-R); ISCIII/FEDER (RD16/0011/0024); ACCIÓ/FEDER (AdvanceCat); Fundació la Marató de TV3 (201534-30); and CERCA Program/Generalitat de Catalunya.

Received: May 3, 2018
Revised: July 12, 2019
Accepted: July 15, 2019
Published: August 8, 2019

REFERENCES

Ayllon, V., Bueno, C., Ramos-Mejia, V., Navarro-Montero, O., Prieto, C., Real, P.J., Romero, T., Garcia-Leon, M.J., Toribio, M.L., Bigas, A., et al. (2015). The Notch ligand DLL4 specifically marks human hematoendothelial progenitors and regulates their hematopoietic fate. Leukemia *29*, 1741–1753.

Boyle, E.I., Weng, S., Gollub, J., Jin, H., Botstein, D., Cherry, J.M., and Sherlock, G. (2004). GO::TermFinder–open source software for accessing Gene Ontology information and finding significantly enriched Gene Ontology terms associated with a list of genes. Bioinformatics *20*, 3710–3715.

Bresnick, E.H., Katsumura, K.R., Lee, H.Y., Johnson, K.D., and Perkins, A.S. (2012). Master regulatory GATA transcription factors: mechanistic principles and emerging links to hematologic malignancies. Nucleic Acids Res. *40*, 5819–5831.

Bussmann, J., Bakkers, J., and Schulte-Merker, S. (2007). Early endocardial morphogenesis requires Scl/Tal1. PLoS Genet. *3*, e140.

Butler, A., Hoffman, P., Smibert, P., Papalexi, E., and Satija, R. (2018). Integrating single-cell transcriptomic data across different conditions, technologies, and species. Nat. Biotechnol. *36*, 411–420.

Calero-Nieto, F.J., Ng, F.S., Wilson, N.K., Hannah, R., Moignard, V., Leal-Cervantes, A.I., Jimenez-Madrid, I., Diamanti, E., Wernisch, L., and Gottgens, B. (2014). Key regulators control distinct transcriptional programmes in blood progenitor and mast cells. EMBO J. *33*, 1212–1226.

Chagraoui, H., Kristiansen, M.S., Ruiz, J.P., Serra-Barros, A., Richter, J., Hall-Ponsele, E., Gray, N., Waithe, D., Clark, K., Hublitz, P., et al. (2018). SCL/TAL1 cooperates with Polycomb RYBP-PRC1 to suppress alternative lineages in blood-fated cells. Nat. Commun. *9*, 5375.

Chan, S.S., Shi, X., Toyama, A., Arpke, R.W., Dandapat, A., Iacovino, M., Kang, J., Le, G., Hagen, H.R., Garry, D.J., et al. (2013). Mesp1 patterns mesoderm into cardiac, hematopoietic, or skeletal myogenic progenitors in a context-dependent manner. Cell Stem Cell *12*, 587–601.

Choi, K.D., Yu, J., Smuga-Otto, K., Salvagiotto, G., Rehrauer, W., Vodyanik, M., Thomson, J., and Slukvin, I. (2009). Hematopoietic and endothelial differentiation of human induced pluripotent stem cells. Stem Cells *27*, 559–567.

de Pater, E., Kaimakis, P., Vink, C.S., Yokomizo, T., Yamada-Inagawa, T., van der Linden, R., Kartalaei, P.S., Camper, S.A., Speck, N., and Dzierzak, E. (2013). Gata2 is required for HSC generation and survival. J. Exp. Med. *210*, 2843–2850.

Dickinson, R.E., Griffin, H., Bigley, V., Reynard, L.N., Hussain, R., Haniffa, M., Lakey, J.H., Rahman, T., Wang, X.N., McGovern, N., et al. (2011). Exome sequencing identifies GATA-2 mutation as the cause of dendritic cell, monocyte, B and NK lymphoid deficiency. Blood *118*, 2656–2658.

Doulatov, S., Vo, L.T., Chou, S.S., Kim, P.G., Arora, N., Li, H., Hadland, B.K., Bernstein, I.D., Collins, J.J., Zon, L.I., et al. (2013). Induction of multipotential hematopoietic progenitors from human pluripotent stem cells via respecification of lineage-restricted precursors. Cell Stem Cell *13*, 459–470.

Dzierzak, E., and Speck, N.A. (2008). Of lineage and legacy: the development of mammalian hematopoietic stem cells. Nat. Immunol. *9*, 129–136.

Elcheva, I., Brok-Volchanskaya, V., Kumar, A., Liu, P., Lee, J.H., Tong, L., Vodyanik, M., Swanson, S., Stewart, R., Kyba, M., et al. (2014). Direct induction of haematoendothelial programs in human pluripotent stem cells by transcriptional regulators. Nat. Commun. *5*, 4372.

Espin-Palazon, R., Stachura, D.L., Campbell, C.A., Garcia-Moreno, D., Del Cid, N., Kim, A.D., Candel, S., Meseguer, J., Mulero, V., and Traver, D. (2014). Proinflammatory signaling regulates hematopoietic stem cell emergence. Cell *159*, 1070–1085.

Freire, A.G., Waghray, A., Soares-da-Silva, F., Resende, T.P., Lee, D.F., Pereira, C.F., Nascimento, D.S., Lemischka, I.R., and Pinto-do, O.P. (2017). Transient HES5 activity instructs mesodermal cells toward a cardiac fate. Stem Cell Reports *9*, 136–148.

Fujiwara, T., O'Geen, H., Keles, S., Blahnik, K., Linnemann, A.K., Kang, Y.A., Choi, K., Farnham, P.J., and Bresnick, E.H. (2009).

Discovering hematopoietic mechanisms through genome-wide analysis of GATA factor chromatin occupancy. Mol. Cell *36*, 667–681.

Gao, X., Johnson, K.D., Chang, Y.I., Boyer, M.E., Dewey, C.N., Zhang, J., and Bresnick, E.H. (2013). Gata2 cis-element is required for hematopoietic stem cell generation in the mammalian embryo. J. Exp. Med. *210*, 2833–2842.

Giorgetti, A., Castano, J., Bueno, C., Diaz de la Guardia, R., Delgado, M., Bigas, A., Espinosa, L., and Menendez, P. (2017). Proinflammatory signals are insufficient to drive definitive hematopoietic specification of human HSCs in vitro. Exp. Hematol. *45*, 85–93.e82.

Gomes, A.M., Kurochkin, I., Chang, B., Daniel, M., Law, K., Satija, N., Lachmann, A., Wang, Z., Ferreira, L., Ma'ayan, A., et al. (2018). Cooperative transcription factor induction mediates hemogenic reprogramming. Cell Rep. *25*, 2821–2835.e7.

Hahn, C.N., Chong, C.E., Carmichael, C.L., Wilkins, E.J., Brautigan, P.J., Li, X.C., Babic, M., Lin, M., Carmagnac, A., Lee, Y.K., et al. (2011). Heritable GATA2 mutations associated with familial myelodysplastic syndrome and acute myeloid leukemia. Nat. Genet. *43*, 1012–1017.

He, Q., Zhang, C., Wang, L., Zhang, P., Ma, D., Lv, J., and Liu, F. (2015). Inflammatory signaling regulates hematopoietic stem and progenitor cell emergence in vertebrates. Blood *125*, 1098–1106.

Huang, J., Liu, X., Li, D., Shao, Z., Cao, H., Zhang, Y., Trompouki, E., Bowman, T.V., Zon, L.I., Yuan, G.C., et al. (2016). Dynamic control of enhancer repertoires drives lineage and stage-specific transcription during hematopoiesis. Dev. Cell *36*, 9–23.

Ismailoglu, I., Yeamans, G., Daley, G.Q., Perlingeiro, R.C., and Kyba, M. (2008). Mesodermal patterning activity of SCL. Exp. Hematol. *36*, 1593–1603.

Ivanovs, A., Rybtsov, S., Anderson, R.A., Turner, M.L., and Medvinsky, A. (2014). Identification of the niche and phenotype of the first human hematopoietic stem cells. Stem Cell Reports *2*, 449–456.

Ivanovs, A., Rybtsov, S., Welch, L., Anderson, R.A., Turner, M.L., and Medvinsky, A. (2011). Highly potent human hematopoietic stem cells first emerge in the intraembryonic aorta-gonad-mesonephros region. J. Exp. Med. *208*, 2417–2427.

Johnson, K.D., Hsu, A.P., Ryu, M.J., Wang, J., Gao, X., Boyer, M.E., Liu, Y., Lee, Y., Calvo, K.R., Keles, S., et al. (2012). Cis-element mutated in GATA2-dependent immunodeficiency governs hematopoiesis and vascular integrity. J. Clin. Invest. *122*, 3692–3704.

Kang, H., Mesquitta, W.T., Jung, H.S., Moskvin, O.V., Thomson, J.A., and Slukvin, I.I. (2018). GATA2 is dispensable for specification of hemogenic endothelium but promotes endothelial-to-hematopoietic transition. Stem Cell Reports *11*, 197–211.

Katsumura, K.R., Bresnick, E.H., and Group, G.F.M. (2017). The GATA factor revolution in hematology. Blood *129*, 2092–2102.

Kouskoff, V., Lacaud, G., Schwantz, S., Fehling, H.J., and Keller, G. (2005). Sequential development of hematopoietic and cardiac mesoderm during embryonic stem cell differentiation. Proc. Natl. Acad. Sci. U S A *102*, 13170–13175.

Koyano-Nakagawa, N., and Garry, D.J. (2017). Etv2 as an essential regulator of mesodermal lineage development. Cardiovasc. Res. *113*, 1294–1306.

Lee, D., Park, C., Lee, H., Lugus, J.J., Kim, S.H., Arentson, E., Chung, Y.S., Gomez, G., Kyba, M., Lin, S., et al. (2008). ER71 acts downstream of BMP, Notch, and Wnt signaling in blood and vessel progenitor specification. Cell Stem Cell *2*, 497–507.

Lian, X., Zhang, J., Azarin, S.M., Zhu, K., Hazeltine, L.B., Bao, X., Hsiao, C., Kamp, T.J., and Palecek, S.P. (2013). Directed cardiomyocyte differentiation from human pluripotent stem cells by modulating Wnt/beta-catenin signaling under fully defined conditions. Nat. Protoc. *8*, 162–175.

Lim, K.C., Hosoya, T., Brandt, W., Ku, C.J., Hosoya-Ohmura, S., Camper, S.A., Yamamoto, M., and Engel, J.D. (2012). Conditional Gata2 inactivation results in HSC loss and lymphatic mispatterning. J. Clin. Invest. *122*, 3705–3717.

Ling, K.W., Ottersbach, K., van Hamburg, J.P., Oziemlak, A., Tsai, F.Y., Orkin, S.H., Ploemacher, R., Hendriks, R.W., and Dzierzak, E. (2004). GATA-2 plays two functionally distinct roles during the ontogeny of hematopoietic stem cells. J. Exp. Med. *200*, 871–882.

Liu, F., Bhang, S.H., Arentson, E., Sawada, A., Kim, C.K., Kang, I., Yu, J., Sakurai, N., Kim, S.H., Yoo, J.J., et al. (2013). Enhanced hemangioblast generation and improved vascular repair and regeneration from embryonic stem cells by defined transcription factors. Stem Cell Reports *1*, 166–182.

Liu, F., Kang, I., Park, C., Chang, L.W., Wang, W., Lee, D., Lim, D.S., Vittet, D., Nerbonne, J.M., and Choi, K. (2012). ER71 specifies Flk-1+ hemangiogenic mesoderm by inhibiting cardiac mesoderm and Wnt signaling. Blood *119*, 3295–3305.

Merika, M., and Orkin, S.H. (1993). DNA-binding specificity of GATA family transcription factors. Mol. Cell. Biol. *13*, 3999–4010.

Molkentin, J.D. (2000). The zinc finger-containing transcription factors GATA-4, -5, and -6. Ubiquitously expressed regulators of tissue-specific gene expression. J. Biol. Chem. *275*, 38949–38952.

Nakajima-Takagi, Y., Osawa, M., Oshima, M., Takagi, H., Miyagi, S., Endoh, M., Endo, T.A., Takayama, N., Eto, K., Toyoda, T., et al. (2013). Role of SOX17 in hematopoietic development from human embryonic stem cells. Blood *121*, 447–458.

Navarro-Montero, O., Ayllon, V., Lamolda, M., Lopez-Onieva, L., Montes, R., Bueno, C., Ng, E., Guerrero-Carreno, X., Romero, T., Romero-Moya, D., et al. (2017). RUNX1c regulates hematopoietic differentiation of human pluripotent stem cells possibly in cooperation with proinflammatory signaling. Stem Cells *35*, 2253–2266.

Org, T., Duan, D., Ferrari, R., Montel-Hagen, A., Van Handel, B., Kerenyi, M.A., Sasidharan, R., Rubbi, L., Fujiwara, Y., Pellegrini, M., et al. (2015). Scl binds to primed enhancers in mesoderm to regulate hematopoietic and cardiac fate divergence. EMBO J. *34*, 759–777.

Orkin, S.H. (1992). GATA-binding transcription factors in hematopoietic cells. Blood *80*, 575–581.

Persons, D.A., Allay, J.A., Allay, E.R., Ashmun, R.A., Orlic, D., Jane, S.M., Cunningham, J.M., and Nienhuis, A.W. (1999). Enforced expression of the GATA-2 transcription factor blocks normal hematopoiesis. Blood *93*, 488–499.

Pijuan-Sala, B., Griffiths, J.A., Guibentif, C., Hiscock, T.W., Jawaid, W., Calero-Nieto, F.J., Mulas, C., Ibarra-Soria, X., Tyser, R.C.V., Ho, D.L.L., et al. (2019). A single-cell molecular map of mouse gastrulation and early organogenesis. Nature *566*, 490–495.

Ramos-Mejia, V., Navarro-Montero, O., Ayllon, V., Bueno, C., Romero, T., Real, P.J., and Menendez, P. (2014). HOXA9 promotes hematopoietic commitment of human embryonic stem cells. Blood *124*, 3065–3075.

Rasmussen, T.L., Kweon, J., Diekmann, M.A., Belema-Bedada, F., Song, Q., Bowlin, K., Shi, X., Ferdous, A., Li, T., Kyba, M., et al. (2011). ER71 directs mesodermal fate decisions during embryogenesis. Development *138*, 4801–4812.

Robert-Moreno, A., Espinosa, L., de la Pompa, J.L., and Bigas, A. (2005). RBPjkappa-dependent Notch function regulates Gata2 and is essential for the formation of intra-embryonic hematopoietic cells. Development *132*, 1117–1126.

Sawamiphak, S., Kontarakis, Z., and Stainier, D.Y. (2014). Interferon gamma signaling positively regulates hematopoietic stem cell emergence. Dev. Cell *31*, 640–653.

Schoenebeck, J.J., Keegan, B.R., and Yelon, D. (2007). Vessel and blood specification override cardiac potential in anterior mesoderm. Dev. Cell *13*, 254–267.

Scialdone, A., Tanaka, Y., Jawaid, W., Moignard, V., Wilson, N.K., Macaulay, I.C., Marioni, J.C., and Gottgens, B. (2016). Resolving early mesoderm diversification through single-cell expression profiling. Nature *535*, 289–293.

Shi, X., Richard, J., Zirbes, K.M., Gong, W., Lin, G., Kyba, M., Thomson, J.A., Koyano-Nakagawa, N., and Garry, D.J. (2014). Cooperative interaction of Etv2 and Gata2 regulates the development of endothelial and hematopoietic lineages. Dev. Biol. *389*, 208–218.

Shivdasani, R.A., Mayer, E.L., and Orkin, S.H. (1995). Absence of blood formation in mice lacking the T-cell leukaemia oncoprotein tal-1/SCL. Nature *373*, 432–434.

Spinner, M.A., Sanchez, L.A., Hsu, A.P., Shaw, P.A., Zerbe, C.S., Calvo, K.R., Arthur, D.C., Gu, W., Gould, C.M., Brewer, C.C., et al. (2014). GATA2 deficiency: a protean disorder of hematopoiesis, lymphatics, and immunity. Blood *123*, 809–821.

Sturgeon, C.M., Ditadi, A., Awong, G., Kennedy, M., and Keller, G. (2014). Wnt signaling controls the specification of definitive and primitive hematopoiesis from human pluripotent stem cells. Nat. Biotechnol. *32*, 554–561.

Sugimura, R., Jha, D.K., Han, A., Soria-Valles, C., da Rocha, E.L., Lu, Y.F., Goettel, J.A., Serrao, E., Rowe, R.G., Malleshaiah, M., et al. (2017). Haematopoietic stem and progenitor cells from human pluripotent stem cells. Nature *545*, 432–438.

Takahashi, K., Tanabe, K., Ohnuki, M., Narita, M., Ichisaka, T., Tomoda, K., and Yamanaka, S. (2007). Induction of pluripotent stem cells from adult human fibroblasts by defined factors. Cell *131*, 861–872.

Tipping, A.J., Pina, C., Castor, A., Hong, D., Rodrigues, N.P., Lazzari, L., May, G.E., Jacobsen, S.E., and Enver, T. (2009). High GATA-2 expression inhibits human hematopoietic stem and progenitor cell function by effects on cell cycle. Blood *113*, 2661–2672.

Tsai, F.Y., Keller, G., Kuo, F.C., Weiss, M., Chen, J., Rosenblatt, M., Alt, F.W., and Orkin, S.H. (1994). An early haematopoietic defect in mice lacking the transcription factor GATA-2. Nature *371*, 221–226.

Tsai, F.Y., and Orkin, S.H. (1997). Transcription factor GATA-2 is required for proliferation/survival of early hematopoietic cells and mast cell formation, but not for erythroid and myeloid terminal differentiation. Blood *89*, 3636–3643.

Van Handel, B., Montel-Hagen, A., Sasidharan, R., Nakano, H., Ferrari, R., Boogerd, C.J., Schredelseker, J., Wang, Y., Hunter, S., Org, T., et al. (2012). Scl represses cardiomyogenesis in prospective hemogenic endothelium and endocardium. Cell *150*, 590–605.

Vicente, C., Conchillo, A., Garcia-Sanchez, M.A., and Odero, M.D. (2012). The role of the GATA2 transcription factor in normal and malignant hematopoiesis. Crit. Rev. Oncol. Hematol. *82*, 1–17.

Wareing, S., Eliades, A., Lacaud, G., and Kouskoff, V. (2012). ETV2 expression marks blood and endothelium precursors, including hemogenic endothelium, at the onset of blood development. Dev. Dyn. *241*, 1454–1464.

Wilson, N.K., Foster, S.D., Wang, X., Knezevic, K., Schutte, J., Kaimakis, P., Chilarska, P.M., Kinston, S., Ouwehand, W.H., Dzierzak, E., et al. (2010a). Combinatorial transcriptional control in blood stem/progenitor cells: genome-wide analysis of ten major transcriptional regulators. Cell Stem Cell *7*, 532–544.

Wilson, N.K., Kent, D.G., Buettner, F., Shehata, M., Macaulay, I.C., Calero-Nieto, F.J., Sanchez Castillo, M., Oedekoven, C.A., Diamanti, E., Schulte, R., et al. (2015). Combined single-cell functional and gene expression analysis resolves heterogeneity within stem cell populations. Cell Stem Cell *16*, 712–724.

Wilson, N.K., Timms, R.T., Kinston, S.J., Cheng, Y.H., Oram, S.H., Landry, J.R., Mullender, J., Ottersbach, K., and Gottgens, B. (2010b). Gfi1 expression is controlled by five distinct regulatory regions spread over 100 kilobases, with Scl/Tal1, Gata2, PU.1, Erg, Meis1, and Runx1 acting as upstream regulators in early hematopoietic cells. Mol. Cell Biol. *30*, 3853–3863.

Wlodarski, M.W., Hirabayashi, S., Pastor, V., Stary, J., Hasle, H., Masetti, R., Dworzak, M., Schmugge, M., van den Heuvel-Eibrink, M., Ussowicz, M., et al. (2016). Prevalence, clinical characteristics, and prognosis of GATA2-related myelodysplastic syndromes in children and adolescents. Blood *127*, 1387–1397, quiz 1518.

Zhou, Y., Zhang, Y., Chen, B., Dong, Y., Zhang, Y., Mao, B., Pan, X., Lai, M., Chen, Y., Bian, G., et al. (2019). Overexpression of GATA2 enhances development and maintenance of human embryonic stem cell-derived hematopoietic stem cell-like progenitors. Stem Cell Reports *13*, 31–47.

Stem Cell Reports, Volume *13*

Supplemental Information

GATA2 Promotes Hematopoietic Development and Represses Cardiac Differentiation of Human Mesoderm

Julio Castaño, Sergi Aranda, Clara Bueno, Fernando J. Calero-Nieto, Eva Mejia-Ramirez, Jose Luis Mosquera, Enrique Blanco, Xiaonan Wang, Cristina Prieto, Lorea Zabaleta, Elisabetta Mereu, Meritxell Rovira, Senda Jiménez-Delgado, Daniel R. Matson, Holger Heyn, Emery H. Bresnick, Berthold Göttgens, Luciano Di Croce, Pablo Menendez, Angel Raya, and Alessandra Giorgetti

Inventory of Supplemental Information

- Figure S1 with Legend
- Figure S2 with Legend
- Figure S3 with Legend
- Figure S4 with Legend
- Figure S5 with Legend
- Table S1
- Table S2
- Table S3
- Table S4
- Movie S1
- Movie S2
- Movie S3
- Supplemental Experimental Procedures
- Supplemental References

Supplemental Figures and legends

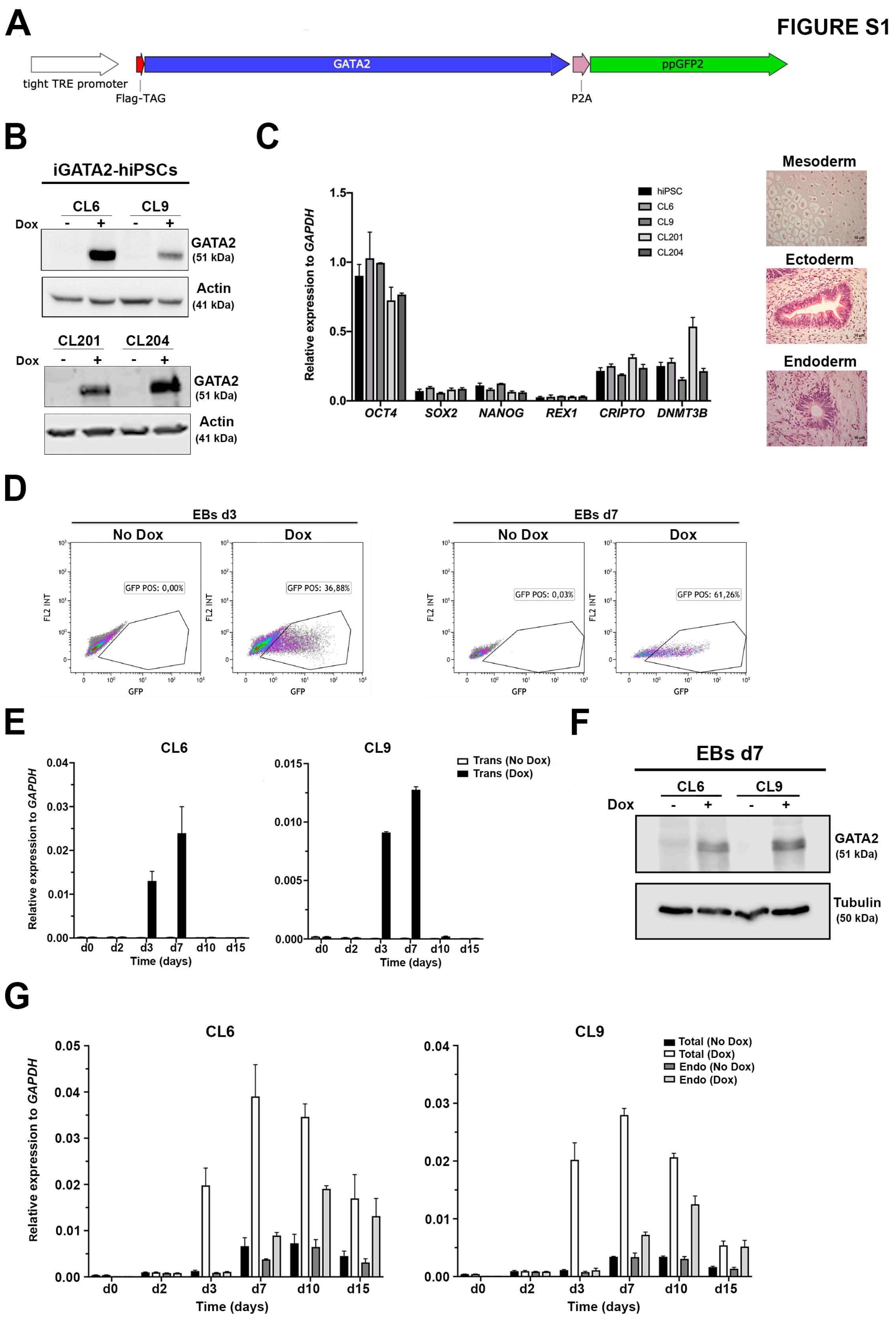

Figure S1. Generation of iGATA2-iPSC lines. Related to Figure 1:
(A) Schematic illustration of the cassette used to generate inducible GATA2-iPSC lines (iGATA2-iPSC). (B) Western blot analysis of GATA2 protein expression in four selected hiPSC lines with and without Dox treatment. (C) qRT-PCR analysis of pluripotency genes (left panel) and teratoma assay (right panel) showing that *GATA2* transgene does not interfere with hiPSC pluripotency. The data represent the mean ± SD of 3 independent experiments. Scale bars are 10 μm. (D) Representative flow cytometry analysis of GFP expression after Dox treatment in EBs d3 and d7. (E) qRT-PCR analysis showing *GATA2* transgene induction in 2 selected hiPSC clones (CL6 and CL9) during EB differentiation. (F) Western blot analysis of GATA2 protein expression in EBs d7 of differentiation for two selected hiPSC lines with and without Dox treatment. (G) qRT-PCR analysis showing endogenous and wild type *GATA2* expression in 2 selected hiPSC clones (CL6 and CL9) during EB differentiation with and without Dox treatment.

A

FIGURE S2

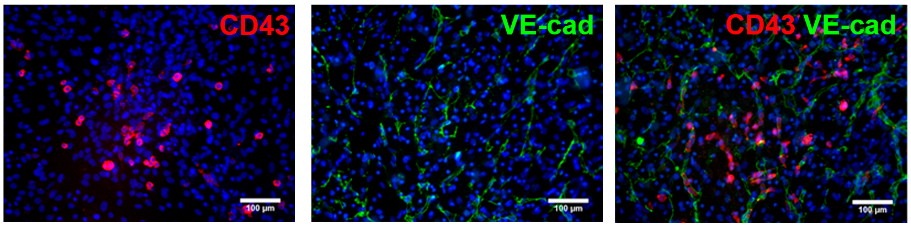

Hematopoietic colonies (HC) Endothelial colonies (EC) Mixed colonies (MC)

Colonies per 192 wells (KDR+CD31-CD34- EBs d5)

Condition	HC	EC	MC	HC/EC
No Dox	3	8	1	0.4
Dox	5	7	3	0.7

Colonies per 288 wells (CD31+CD34+CD43- EBs d7)

Condition	HC	EC	MC	HC/EC
No Dox	8	11	9	0.72
Dox	12	4	12	3

B

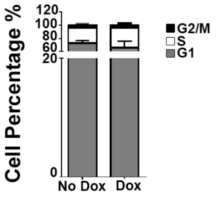

HEPs d10	G1	G2/M	S
No Dox	72.8 ±3.83	4.8±1.19	23.13±3.6
Dox	70.7±2.86	4.53±1.47	26±1.53

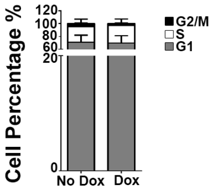

HPCs d15	G1	G2/M	S
No Dox	70.83 ±4.24	4.4±1.11	24.77±11.5
Dox	76.1±2.97	2.93±1.10	27.4±1.10

Figure S2. GATA2 promote hematopoietic differentiation. Related to Figure 2: (A) Representative pictures of hematopoietic (HC), endothelial (EC) and Mixed colonies (MC) derived from single mesodermal cell (KDR+CD31-CD34-; EBs d5) and/or Hemato-endothelial cells (CD31+CD34+CD43-, EBs d7) cultured on OP9 in Dox and No Dox conditions (top table). The number of HC, EC and MC was scored based on CD43 and Ve-cad expression after 7 days of culture (botton table). Scale bars are 100 µm. (B) Flow cytometry analysis of cell cycle of HEPs and HPCs in Dox and No Dox conditions. A similar number of cycling cells (S, G2, and M) were found in HEPs and HPCs of control and Dox-treated cells.

FIGURE S3

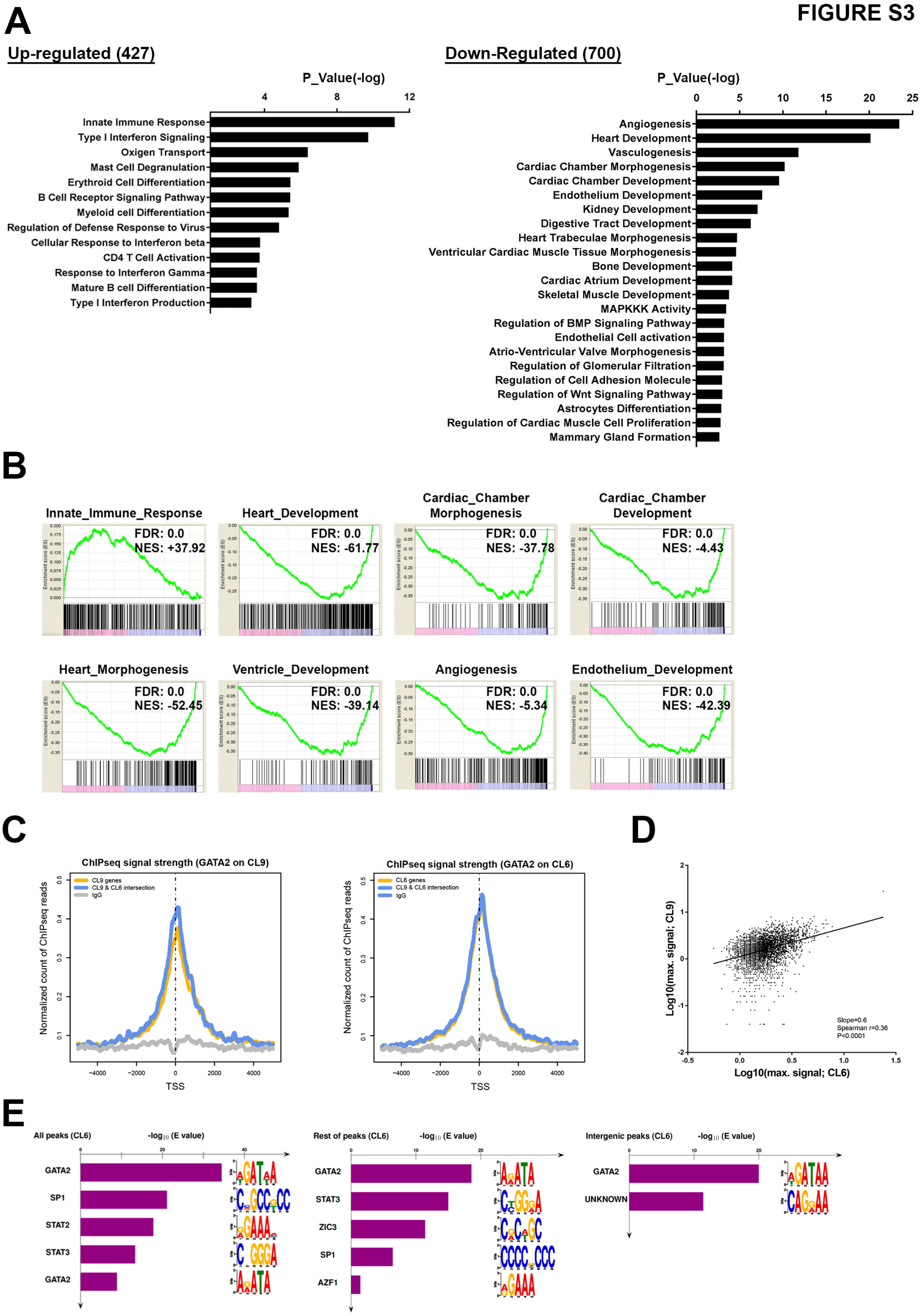
A
Up-regulated (427)
P_Value(-log)
4
8
12
Innate Immune Response
Type I Interferon Signaling
Oxigen Transport
Mast Cell Degranulation
Erythroid Cell Differentiation
B Cell Receptor Signaling Pathway
Myeloid cell Differentiation
Regulation of Defense Response to Virus
Cellular Response to Interferon beta
CD4 T Cell Activation
Response to Interferon Gamma
Mature B cell Differentiation
Type I Interferon Production
Down-Regulated (700)
P_Value(-log)
0
5
10
15
20
25
Angiogenesis
Heart Development
Vasculogenesis
Cardiac Chamber Morphogenesis
Cardiac Chamber Development
Endothelium Development
Kidney Development
Digestive Tract Development
Heart Trabeculae Morphogenesis
Ventricular Cardiac Muscle Tissue Morphogenesis
Bone Development
Cardiac Atrium Development
Skeletal Muscle Development
MAPKKK Activity
Regulation of BMP Signaling Pathway
Endothelial Cell activation
Atrio-Ventricular Valve Morphogenesis
Regulation of Glomerular Filtration
Regulation of Cell Adhesion Molecule
Regulation of Wnt Signaling Pathway
Astrocytes Differentiation
Regulation of Cardiac Muscle Cell Proliferation
Mammary Gland Formation
B
Innate_Immune_Response
FDR: 0.0
NES: +37.92
Heart_Development
FDR: 0.0
NES: -61.77
Cardiac_Chamber Morphogenesis
FDR: 0.0
NES: -37.78
Cardiac_Chamber Development
FDR: 0.0
NES: -4.43
Heart_Morphogenesis
FDR: 0.0
NES: -52.45
Ventricle_Development
FDR: 0.0
NES: -39.14
Angiogenesis
FDR: 0.0
NES: -5.34
Endothelium_Development
FDR: 0.0
NES: -42.39
C
ChIPseq signal strength (GATA2 on CL9)
CL9 genes
CL9 & CL6 intersection
IgG
Normalized count of ChIPseq reads
TSS
ChIPseq signal strength (GATA2 on CL6)
CL6 genes
CL9 & CL6 intersection
IgG
Normalized count of ChIPseq reads
TSS
D
Log10(max. signal; CL9)
Log10(max. signal; CL6)
Slope=0.6
Spearman r=0.36
P<0.0001
E
All peaks (CL6)
-log10 (E value)
GATA2
SP1
STAT2
STAT3
GATA2
Rest of peaks (CL6)
-log10 (E value)
GATA2
STAT3
ZIC3
SP1
AZF1
Intergenic peaks (CL6)
-log10 (E value)
GATA2
UNKNOWN

Figure S3. Gene expression and ChIP-Seq analysis in GATA2 induced Cells. Related to Figure 3: (A) GO function classification analysis of up-regulated and down-regulated genes in GATA2-induced HEPs compared with control cells (HEPs No-Dox). (B) Gene set enrichment analysis (GSEA) showing genes up- and downregulated in HEPs at day 10 of EB differentiation following Dox-inducible *GATA2* expression from day 2 to 7. The false discovery rate (FDR) q value and normalized enrichment score (NES) are shown. (C) Meta-gene plots showing the GATA2 ChIP-seq occupancy profile on CL9 or CL6 replicates for the respective target genes found in each replicate and the common bona fide GATA2 target genes. IgG distribution is indicated with the grey line. (D) Correlation plot between GATA2 ChIP-seq replicates CL6 and CL9 replicates on CL6 peaks found by MACS in CL6. (E) The histograms indicate the Top ranked motifs identified by DREME tool.

FIGURE S4

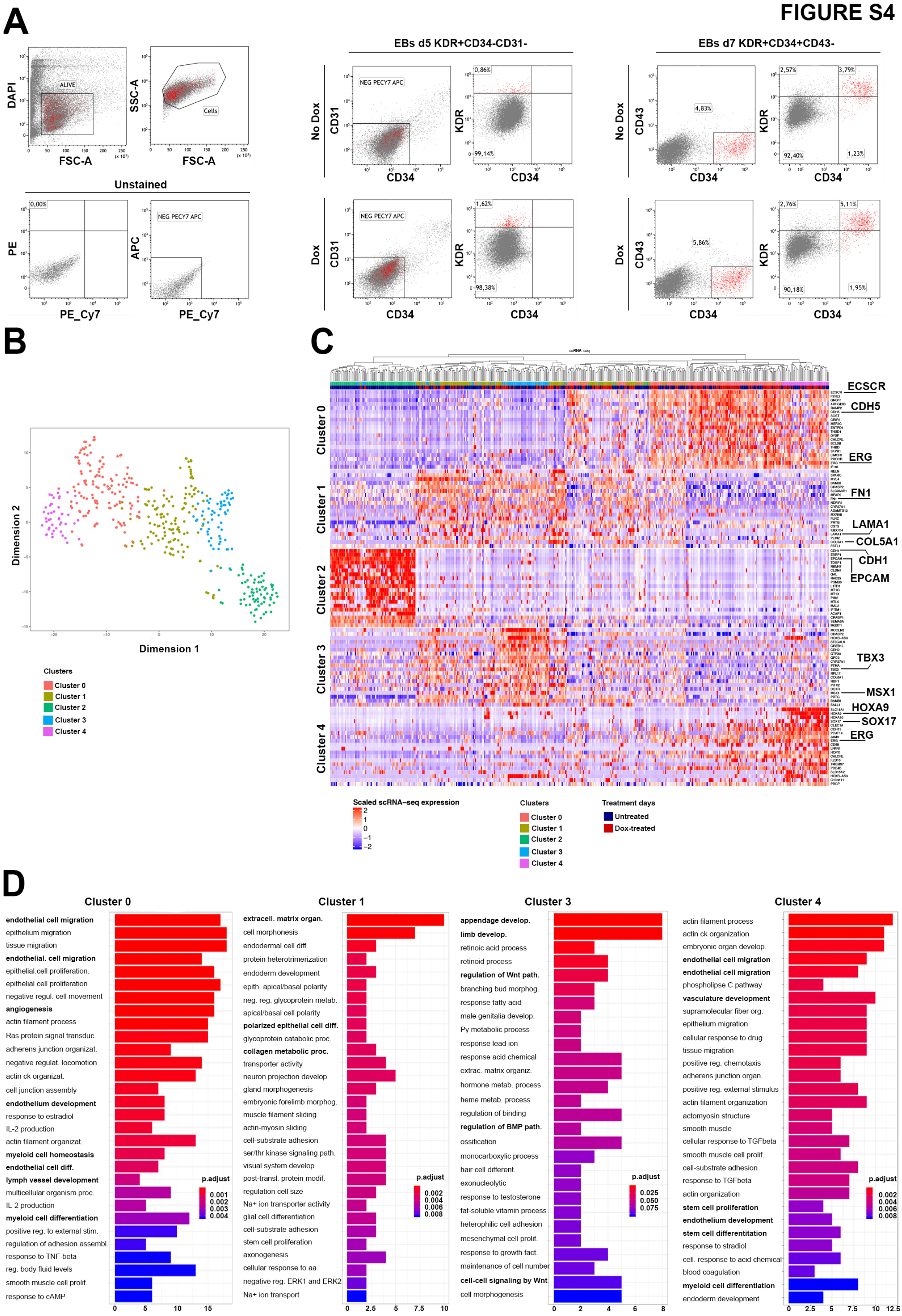

A
DAPI
FSC-A
ALIVE
SSC-A
Cells
Unstained
PE
PE_Cy7
APC
NEG PECY7 APC
EBs d5 KDR+CD34-CD31-
EBs d7 KDR+CD34+CD43-
No Dox
Dox
CD31
CD34
KDR
CD43
B
Dimension 2
Dimension 1
Clusters
Cluster 0
Cluster 1
Cluster 2
Cluster 3
Cluster 4
C
ECSCR
CDH5
ERG
FN1
LAMA1
COL5A1
CDH1
EPCAM
TBX3
MSX1
HOXA9
SOX17
ERG
Scaled scRNA-seq expression
Clusters
Treatment days
Untreated
Dox-treated
D
Cluster 0
endothelial cell migration
epithelium migration
tissue migration
endothelial. cell migration
epithelial.cell proliferation.
epithelial cell proliferation
negative regul. cell movement
angiogenesis
actin filament process
Ras protein signal transduc.
adherens junction organizat.
negative regulat. locomotion
actin ck organizat.
cell junction assembly
endothelium development
response to estradiol
IL-2 production
actin filament organizat.
myeloid cell homeostasis
endothelial cell diff.
lymph vessel development
multicellular organism proc.
IL-2 production
myeloid cell differentiation
positive reg. to external stim.
regulation of adhesion assembl.
response to TNF-beta
reg. body fluid levels
smooth muscle cell prolif.
response to cAMP
p.adjust
Cluster 1
extracell. matrix organ.
cell morphogenesis
endodermal cell diff.
protein heterotrimerization
endoderm development
epith. apical/basal polarity
neg. reg. glycoprotein metab.
apical/basal cell polarity
polarized epithelial cell diff.
glycoprotein catabolic proc.
collagen metabolic proc.
transporter activity
neuron projection develop.
gland morphogenesis
embryonic forelimb morphog.
muscle filament sliding
actin-myosin sliding
cell-substrate adhesion
ser/thr kinase signaling path.
visual system develop.
post-transl. protein modif.
regulation cell size
Na+ ion transporter activity
glial cell differentiation
cell-substrate adhesion
stem cell proliferation
axonogenesis
cellular response to aa
negative reg. ERK1 and ERK2
Na+ ion transport
p.adjust
Cluster 3
appendage develop.
limb develop.
retinoic acid process
retinoid process
regulation of Wnt path.
branching bud morphog.
response fatty acid
male genitalia develop.
Py metabolic process
response lead ion
response acid chemical
extrac. matrix organiz.
hormone metab. process
heme metab. process
regulation of binding
regulation of BMP path.
ossification
monocarboxylic process
hair cell different.
exonucleolytic
response to testosterone
fat-soluble vitamin process
heterophilic cell adhesion
mesenchymal cell prolif.
response to growth fact.
maintenance of cell number
cell-cell signaling by Wnt
cell morphogenesis
p.adjust
Cluster 4
actin filament process
actin ck organization
embryonic organ develop.
endothelial cell migration
endothelial cell migration
phospholipse C pathway
vasculature development
supramolecular fiber org.
epithelium migration
cellular response to drug
tissue migration
positive reg. chemotaxis
adherens junction organ.
positive reg. external stimulus
actin filament organization
actomyosin structure
smooth muscle
cellular response to TGFbeta
smooth muscle cell prolif.
cell-substrate adhesion
response to TGFbeta
actin organization
stem cell proliferation
endothelium development
stem cell differentitation
response to stradiol
cell. response to acid chemical
blood coagulation
myeloid cell differentiation
endoderm development
p.adjust

Figure S4. GATA2 induction enhances mesodermal cell generation and the activation of hemato-endothelial program. Related to Figure 4:

(A) Representative Flow cytometry analysis of mesodermal (KDR+CD31-CD34-) and hemato-endothelial (KDR+CD43+CD45-) phenotype in EBs at d5 and d7, following Dox-inducible GATA2 expression from day 2 to 7 of differentiation. (B) t-SNE projection of single cell, based on new clustering using DEGs of d10. (C) Hierarchical clustering showing the expression in single cells of the top 20 DEGs identified (adjusted *p*-value < 0.05) at d10 from bulk RNA-seq. Colored top bar indicates assigned cluster and treatment. Red indicates the highest scaled expression and blue the lowest. (D) Over Representation Analysis (ORA) showing enrichment of genes within GO biological processes for each indicated clusters. Size of the bar represents the number of DEGs for each GO category. The color represents the adjusted *p*-value for each GO category.

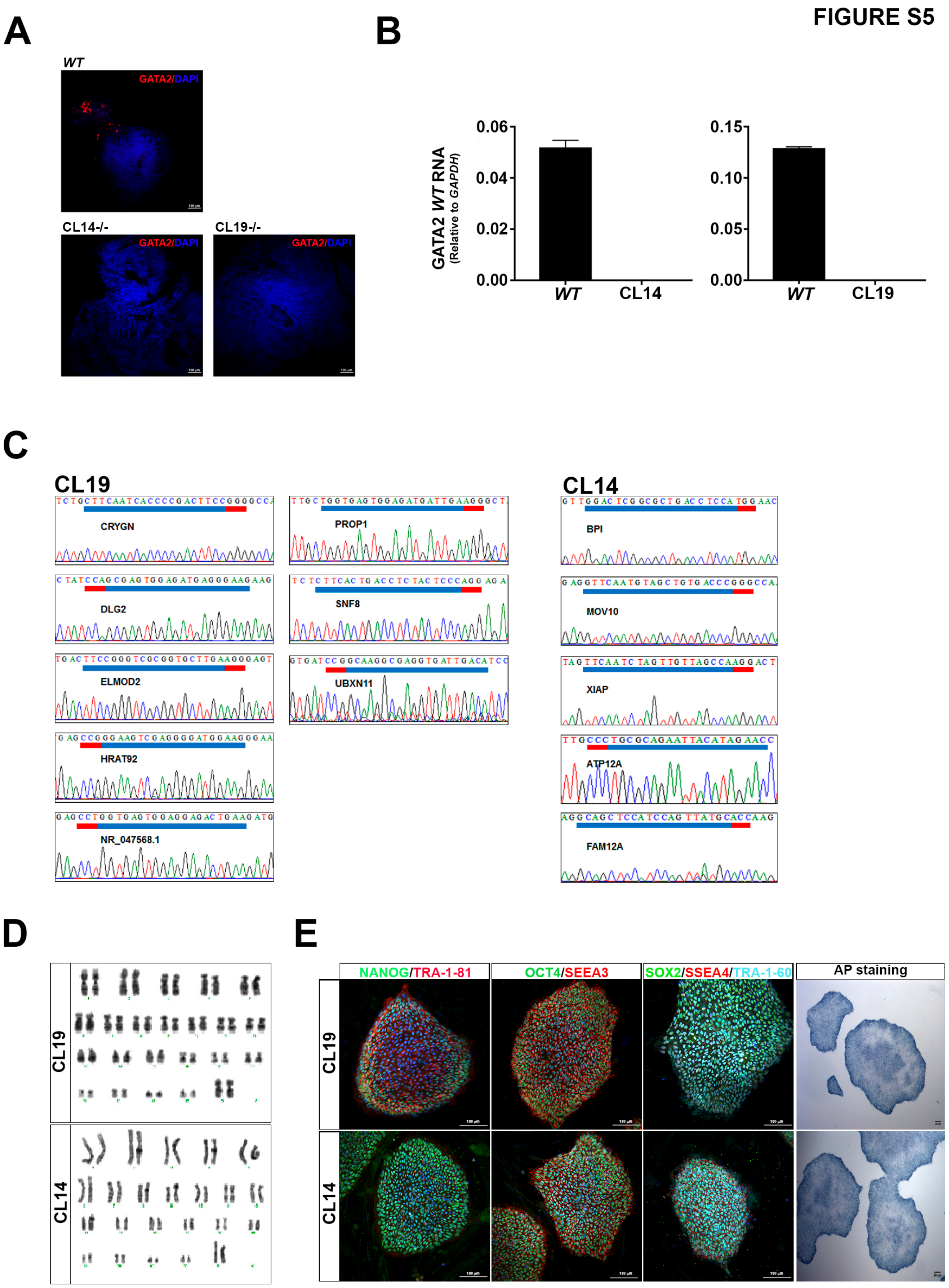

Figure S5. Generation of GATA2^{KO} hiPSC lines. Related to Figure 5: (A) Representative immunofluorescence images of wild-type (WT) and hiPSC-GATA2^{KO} EBs at day 15 of development. GATA2 protein was absent in hiPSC-GATA2^{KO} clones. Scale bars are 100µm. (B) qRT-PCR analysis showing endogenous GATA2 knockout in 2 selected hiPSC clones at day 15 of EB development. (C) Representative histograms of the putative off-target genes in the two selected GATA2^{KO} clones. Blue lines indicate sgRNA homology to target sequence, red lines the PAM sequences. (D and E) Established hiPSC-GATA2^{KO} clones showed normal karyotypes (D) and retained the expression of pluripotency markers (E). Scale bars are 100 µm (pluripotency markers) and 20 µm (AP staining).

Supplemental Tables

Table S1: Primers used for qRT-PCR analysis

Name	Forward primer (5'to 3')	Reverse primer (5' to 3')
**hGAPDH**	GCACCGTCAAGGCTGAGAAC	AGGGATCTCGCTCCTGGAA
**Pluripotency**		
**hCRIPTO**	CGGAACTGTGAGCACGATGT	GGGCAGCCAGGTGTCATG
**hDNMT3B**	GCTCACAGGGCCCGATACTT	GCAGTCCTGCAGCTCGAGTTTA
**hNANOG**	ACAACTGGCCGAAGAATAGCA	GGTTCCCAGTCGGGTTCAC
**hOCT4**	GGGTTTTTGGGATTAAGTTCTTCA	GCCCCCACCCTTTGTGTT
**hREX1**	CCTGCAGGCGGAAATAGAAC	GCACACATAGCCATCACATAAGG
**hSOX2**	CAAAAATGGCCATGCAGGTT	AGTTGGGATCGAACAAAAGCTATT
**Hematopoietic**		
**GATA2 TRANS**	ACTACAAGGACGACGATGACAAG	CGAGTCGAGGTGATTGAAGAAGA
**hGATA2 3'**	AGCTTTACTGTGGCTGTCTGGAT	CCGTCACCGCATACAGAATCTA
**hBRACHYURY**	ATGAGCCTCGAATCCACATAGT	TCCTCGTTCTGATAAGCAGTCA
**hGATA1**	ACCTGCACTGCCTTCATCACT	AAAGTCAGGGCCCCCATAAG
**hMIXL1**	CCGAGTCCAGGATCCAGGTA	CCACTCTGACGCCGAGACTT
**hPU.1**	TGCAAAATGGAAGGGTTTCC	GTCATAGGGCACCAGGTCTTCT
**hRUNX1**	GTGGTCCTATTTAAGCCAGCCC	CTGAAGACACCAGCTTGACAGTTC
**hSCL/TAL1**	GCGGGCCCAGCACCTTT	ATGCAGAGACCAACGCAATTC
**hSOX17**	TGGCGCAGCAGAATCCA	CCACGACTTGCCCAGCAT
**Cardiac**		
**hMYOCD**	ACCAGTCAGATGCGGGGAA	CCAAGGATTTGGACTTTACAGCA
**hNKX2.5**	CTTCTATCCACGTGCCTACAGC	CGCACAGCTCTTTCTTTTCGG
**hTBX3**	CACCAACAACATTTCAGACAAACA	TGCCACGTAGCGTGATCACT
**hTNNC1**	GGACGACAGCAAAGGGAAATCT	TCCAGGTCGATGTAGCCATCAG

Supplemental Experimental Procedures

Human iPSC culture

Human iPSCs were generated from CB-derived CD133+ cells using integrative (retrovirus, line CBI08#4) and non-integrative vectors (Sendai virus, line CBI01#40-Sv), as described (Arellano-Viera et al., 2019; Ban et al., 2011; Giorgetti et al., 2009). Human iPSC lines were maintained in a feeder-free culture system on Matrigel (BD Biosciences)-coated 60-mm plates in mTESR1 medium (StemCell Technologies). The culture medium was changed daily and cells were passaged weekly by dissociation with EDTA. Approval from the Spanish National Embryo Ethical Committee was obtained to work with hPSCs.

Genome engineering

Generation of inducible GATA2-hiPSC lines was performed using the Lenti-X™ Tet-On® Advanced Inducible Expression System (Clontech; Cat#632162). GATA2 cDNA was PCR amplified and cloned in frame with a vector containing a P2A-GFP cassette. GATA2-GFP was subcloned with EcoRI in pLVX-TRE-tight vector. CMV promoter of the pTet-On Advanced vector was replaced by Ubiquin C promoter in order to avoid promoter silencing in hiPSCs (Figure S1A). Cells were transduced by lentivirus infection at a multiplicity of infection of 15, and single-cell-derived clones carrying GATA2 transgene were selected in puromycin (0.25 µg/mL) and neomycin (75 µg/mL).

GATA2-knockout hiPSCs (iPSC-GATA2^{KO}) were generated using CRISPR/Cas9 technology. The CRISPR Design Tool (http://tools.genome-engineering.org) was used for guide RNA design, and the site-specific cleavage efficiency of up to four guide RNAs was tested in 293T cells using the T7 surveyor assay (Ran et al., 2013). Undifferentiated hiPSCs were treated with 10 µM ROCK inhibitor (Y27632; Sigma) for 24 hours and 2 × 10^5 cells were electroporated using the Neon® Transfection System 10 µL Kit (Thermo Fisher Scientific). Cells were transfected with 5 µg of pSpCas9(BB)-2A-GFP (PX458) (Addgene; #48138, www.addgene.org), allowing the expression of both the guide RNA and the Cas9-GFP. At 3 days post-transfection, cells were GFP-sorted and plated as single cells on Matrigel-coated 48-well plates. Single-cell-derived clones were expanded and screened by sequencing. Previously predicted off-targets identified using CRISPR Design Tool were analyzed in iPSC-GATA2^{KO} cells by Sanger sequencing.

Embryoid body-based hematopoietic differentiation

Human iPSCs were differentiated as previously described (Giorgetti et al., 2017). Briefly, for EB generation, undifferentiated hiPSCs were dissociated with collagenase IV, scraped off gently from the Matrigel, transferred to low-attachment plates, and incubated overnight in mTESR1 medium supplemented with bone morphogenetic protein 4 (BMP-4; 50 ng/mL). The following day (day 1), the medium was changed for serum-free defined medium (StemPro-34; Invitrogen) supplemented with monothioglycerol (0.16 µM; Sigma), holo-transferrin (150 µg/mL; Sigma), 50 ng/mL BMP-4, and 10 ng/mL FGFb (both purchased from R&D Systems). To promote the definitive hematopoietic program, EBs were treated with the GSK3 inhibitor CHIR99021 (3 µM) from day 2 to day 3 of culture (Sturgeon et al., 2014). On the third day of differentiation, EBs were changed to differentiation medium comprising serum-free defined medium (StemPro-34; Invitrogen) supplemented with monothioglycerol, holo-transferrin, 50 ng/mL BMP-4, 300 ng/mL Fms-related tyrosine kinase 3 ligand (Flt-3L), 300 ng/mL stem cell factor (SCF), 10 ng/mL IL-3 and 10 ng/mL IL-6 (all purchased from R&D Systems). EBs were cultured until day 15 with medium changes every 3 days. Dissociated cells were analyzed by flow cytometry for the presence of HEPs (CD31+CD34+CD45- and CD34+CD43-CD45-), early HPCs (CD34+CD43+CD45+), and mature blood cells (CD45+), at day 10 and day 15 of differentiation.

Hemogenic endothelial progenitors/ OP9 cocultures

Hemogenic endothelial progenitor cell-potential was assayed as described (Ramos-Mejia et al., 2014). Briefly, day 10 EBs were dissociated and HEPs were purified by magnetic bead separation using the CD34 Microbead Kit (Miltenyi). Purified HEPs (3 x 10^4) were plated on OP9 stroma on 6-well plates with differentiation medium. Cells were harvested at day 4 and analyzed by FACS as described above.

Clonal experiments with FACS-sorted single mesodermal and hemato-endothelial cells

Single mesodermal (KDR+CD34-CD31-) and hemato-endothelial (CD31CD34+CD43-) cells FACS sorted from day 5 and day 7 EBs respectively, were transferred to individual wells of 96-well plates containing OP9 feeder cells. The cells were cultured for up to 10 days with hematopoietic differentiation medium with/without Dox. Fresh medium was provided every other day. Culture plates were fixed and

stained with CD144 (rabbit, eBioscience) and anti-CD43 (mouse, BD Bioscience). Hematopoietic, endothelial and mix clusters were analyzed using a fluorescent microscope.

Flow cytometry analysis and FACS

Differentiating EBs were dissociated with collagenase B (Roche) for 2 hours at 37°C and then incubated with Cell Dissociation Buffer (Invitrogen) for 20 minutes at 37°C. Single-cell suspensions were stained with anti-CD31-APC (Miltenyi), anti-CD34-PE, anti-CD45-APC-H7, anti-CD43-APC, anti-CD34-PE-Cy7 (all from BD Biosciences). hiPSC-OP9-cocultures were treated for 1 hour with collagenase IV, incubated for 20 minutes with TrypLE (Invitrogen), dissociated by pipetting, and filtered through a 70-µm cell strainer. Single-cell suspensions were stained with anti-CD34-PE, anti-CD31-APC, anti-CD45-APC-H7, and anti-mouse CD29-FITC (Miltenyi) to exclude OP9 cells from further analysis. Cardiomyocytes were stained with anti-MHC-PE (IgG2b, 1:400 BD Biosciences) and anti-cTnI-Alexa Flour 647 (IgG2b, 1:100, BD Biosciences). Mouse IgG2b PE (1:400 BD Biosciences) and mouse IgG2b Alexa Fluor 647 (1:100, BD Biosciences) antibodies were used as isotype controls.

Live cells identified by propidium iodide (PI) exclusion were analyzed on a Flow Cytometer (Gallios, Beckman Coulter) equipped with Kaluza analysis software, or on a FACSCanto II cytometer equipped with FACS Diva analysis software (BD Biosciences). Sorting of purified mesodermal and hemato-endothelial cells was performed using a FACSAria cell sorter (BD Biosciences), as described (Ramos-Mejia et al., 2014).

CFU assay

CFU assays were performed by plating 50000 cells from day 10 EBs onto serum-free methylcellulose (MethoCult® SF H4436; StemCell Technologies). Colonies were counted 14 days later using standard morphological criteria.

Cell cycle analysis

EBs at day 10 and day 15 were dissociated and fixed in 70% cold ethanol and stored at -20°C. The next day, the cells were stained with anti-CD34-FITC, anti-CD31-FITC, and anti-CD45-APC for 15 minutes. After washing, the cells were suspended in PBS and 50 µg/mL PI. Cell cycle distribution was analyzed on a FACSCanto II cytometer using Motif software. Cell proliferation was assessed using the Click-iT Plus EdU cell proliferation kit (molecular Probe). EBs were incubated with 10 µM 5-ethylnyl-2'-deoxyuridine (EdU) for 5 hours, dissociated and single-cell suspensions were analyzed by flow cytometry as previously described.

Apoptosis analysis

Apoptosis in HEPs (CD31+CD34+CD45-) and HPCs (CD34+CD45+) was analyzed by flow cytometry using the Annexin V Apoptosis Detection Kit (BD Biosciences).

Western blotting

Human iPSC lines and day 7 differentiating EBs were collected, washed in PBS and lysed in 50 µl RIPA Buffer (Tris-HCl pH 8, 50 mM, NaCl 150 mM, 1% NP-40, 0.5% deoxycholate, 0.1% SDS) with 0.25 mM PMSF and complete protease inhibitor cocktail (Roche) for 30 minutes on ice. Lysates were centrifuged at maximum speed for 10 minutes at 4ºC. Protein supernatant extracts were quantified using the Bio-Rad Protein Assay. For electrophoretic separation, 40 µg of protein was run on 8% SDS-polyacrylamide gels and transferred to PVDF membranes that were then blocked with 5% BSA (Sigma) in TBS / 0.5% Tween-20 (TBS-T), and incubated overnight at 4ºC with the appropriate antibody in TBS-T with 5% BSA. Membranes were washed with TBS-T three times and incubated with an HRP-conjugated secondary antibody for 1 hour. Immunoreactive proteins were detected using the Amersham ECL Prime Western Blotting Detection Reagent (GE Healthcare). Primary antibodies used were anti-GATA2 rabbit polyclonal antibody (Im et al., 2005), anti-GATA2 (Cell Signaling Technologies #4595; rabbit 1:500), anti-β actin (Sigma; mouse 1:5000) and anti-Tubulin (Sigma, mouse 1:500).

RNA purification and quantitative RT-PCR

Total RNA was isolated using the RNAqueous-Micro Kit (Ambion). RNA (1 µg) was retrotranscribed to cDNA using the SuperScript III Reverse Transcriptase Kit (Invitrogen). Quantitative real-time PCR (qPCR) was performed using SYBR Green or IDT PrimeTime chemistry. Expression of *GAPDH* was used to normalize data. Primer sequences are listed in Table S1.

RNA-sequencing

HEPs were FACS-purified from three independent biological replicates (CL6, CL9 and CL201) in Dox and no Dox conditions. For each clone, we analyzed three different pools of 50 HEP cells in Dox and No Dox conditions. Pools of 50 cells were sorted directly into 2.3 µL of lysis buffer (0.2 % (v/v) Triton X-100 and 2 U/µL RNase inhibitor (Clontech)) in 96-well plates and stored at -80°C. RNA-seq was carried out using the Smart-seq2 protocol according to Picelli et al. (Picelli et al., 2014). Libraries were prepared using the Illumina Nextera XT DNA preparation kit. Pooled libraries were sequenced on the Illumina Hi-Seq 2500 platform. Reads were mapped simultaneously to the *Homo sapiens* genome (version GRCh38.81) and the ERCC sequences using STAR (version 2.5.2a) (Dobin et al., 2013) with default parameters. HTseq-count (Anders et al., 2015) was used to count the number of reads mapped to each gene (default options). Samples with less than 500,000 reads mapping to endogenous RNA, with greater than 20% reads mapping to mitochondrial genes or with less than 8000 genes detected at 10 counts per million were considered low quality and removed from downstream analyses. All samples passed quality control. The data were normalized using size factors (Love et al., 2014) defined by DESeq2. Highly variable genes were identified as described (Brennecke et al., 2013). In brief, we fitted the squared coefficient of variation as a function of the mean normalized ERCC counts and selected the genes that exceeded 50% of biological coefficient of variation. In the fitting procedure, to minimize the skewing effect due to the low expressed genes, only ERCCs with a mean normalized count greater than the 80th centile were used. Genes with an adjusted *p*-value (Benjamini-Hochberg method) less than 0.1 were considered significant. This resulted in 501 highly variable genes. PCA and tSNE dimensionality reduction were performed using prcomp (Krijthe, 2015; Venables and Ripley, 2002) package in R. Differentially expressed genes were identified using DESeq2 (1.16.1) (Love et al., 2014) for all genes with a false discovery rate (Benjamini-Hochberg) lower than 0.1.

Single-cell RNA sequencing

Full-length single-cell RNA sequencing libraries were prepared using the Smart-seq2 protocol (Picelli et al., 2014) with minor modifications. Briefly, freshly harvested single cells were sorted into 96-well plates containing the cell lysis buffer. Reverse transcription was performed using SuperScrpit II (Invitrogen) in the presence of oligo-dT30VN, the template-switching oligonucleotide and betaine. The cDNA was amplified using the KAPA Hifi Hotstart ReadyMix (Kappa Biosystems), ISPCR primer and 24 cycles of amplification. Following purification with Agencourt Ampure XP beads (Beckmann Coulter), product size distribution and quantity were assessed on a Bioanalyzer using a High Sensitvity DNA Kit (Agilent Technologies). In total, 140 pg of the amplified cDNA was fragmented using Nextera® XT (Illumina) and amplified with indexed Nextera® PCR primers. Products were purified twice with Agencourt Ampure XP beads and quantified again using a Bioanalyzer High Sensitvity DNA Kit. Sequencing of Nextera® libraries from 384 cells was carried out using one sequencing lane on a Illumina HiSeq2500 v4. The sequencing data were aligned with STAR against the GRCh38 human reference. Cells with less than 65% of mapped reads or a read depth lower than four median absolute deviations from the median of total mapped reads were discarded. Genes expressed in fewer than five cells were also removed. Read counts from 444 single cells (91% of total cells) were used to perform the downstream analyses. Normalization, clustering, gene marker detection and visualization were performed using the Seurat package (version 2.3.4). The number of principal components was selected according to their significance and the resolution parameter set to 0.6, resulting in cluster numbers reflecting the expected biological variability. Cluster-specific markers were then identified based on a Wilcoxon rank-sum test. Chi-squared tests were used for comparing differences between treatments, and the resulting raw *p*-values where adjusted by the Benjamini-Hochberg false discovery rate (FDR) method (Benjamini and Hochberg, 1995).

Bioinformatics analysis

ChIP-seq samples were mapped against the hg19 human genome assembly using Bowtie with the option –m 1 to discard those reads that could not be uniquely mapped in just one region (Langmead et al., 2009). MACS was run with the default parameters but adjusting the shiftsize to 100 bp, to perform the peak calling against the corresponding control sample (Zhang et al., 2008). The UCSC genome browser (Tyner et al., 2017) was used to provide screenshots. The genome distribution of each set of peaks was calculated by counting the number of peaks fitting on each class of region according to RefSeq annotations. The distal region was defined as the region within 2.5 kbp and 0.5 kbp upstream of the transcriptional start site (TSS), whereas the proximal region was defined as the region within 0.5 kbp and the TSS. Those peaks overlapping more than one genomic feature were proportionally counted the same number of times. To generate the spie charts, we first calculated the genome distribution of all features in the full genome and then we used the Caroline package in R to combine the spie chart of each set of peaks with the full genome distribution (Feitelson, 2003).

Each set of target genes was retrieved by matching the ChIP-seq peaks in the region 2.5 kbp upstream of the TSS until the end of the transcript, as annotated in RefSeq. The Enrichr tool was used to generate the reports of functional GO enrichments and other categories (Kuleshov et al., 2016).
The meta-gene plots showing the average distribution of ChIP-seq reads along the region between the TSS and the transcription end site of each target gene were generated by counting the number of reads on this region and normalizing by the total number of mapped reads of the ChIP-seq sample and the length of the gene according to RefSeq. This value was finally averaged for the total number of genes in the particular gene set.
Motif analysis of the resulting peaks and promoter sequences was performed with the MEME-ChIP software of the MEME suite of programs (Machanick and Bailey, 2011).

Luciferase assay
PITX2 and ISL1 promoter regions were amplified by PCR from human genomic DNA and inserted into the KpnI and NheI sites of pGL-3 Basic (Promega, Madison, WI) to generate reporter plasmids. The nucleotide sequences of primer sets are: PITX2 Fw: GGTACCTGACAAGCCTAGCTCGTTCG and Rv: GCTAGCGGCTCCCAGGTAATTCGCTT; ISL1 Fw: GGTACCCCTAGGTCTATCCAACTTTCGC and Rv: GCTAGCTTCACATCTCTGGGCATTGACTG). The pGL3-TBX3-3′ UTR vector was kindly provided by Prof. Sharon Prince, Cancer Research Laboratory University of Cape Town (Peres et al., 2017). The plasmid pWPI-GATA2, available in our laboratory, and its empty vector pWPI, were used in this study. For luciferase assays, COS7 cells were transfected with 120 ng of reporter plasmid, 500 ng of expression plasmid, and 10 ng of pRL-null (Promega) using Lipofectamine 3000 (ThermoFisher Scientific) and harvested 48 hours after transfection. Luciferase activity was analyzed using Biotek and a dual-luciferase assay kit (Promega).
Each experiment was performed in triplicate, while all experiments were repeated at least three times.

Supplemental References

Anders, S., Pyl, P.T., and Huber, W. (2015). HTSeq--a Python framework to work with high-throughput sequencing data. Bioinformatics *31*, 166-169.
Arellano-Viera, E., Zabaleta, L., Castano, J., Azkona, G., Carvajal-Vergara, X., and Giorgetti, A. (2019). Generation of two transgene-free human iPSC lines from CD133(+) cord blood cells. Stem Cell Res *36*, 101410.
Ban, H., Nishishita, N., Fusaki, N., Tabata, T., Saeki, K., Shikamura, M., Takada, N., Inoue, M., Hasegawa, M., Kawamata, S.*, et al.* (2011). Efficient generation of transgene-free human induced pluripotent stem cells (iPSCs) by temperature-sensitive Sendai virus vectors. Proc Natl Acad Sci U S A *108*, 14234-14239.
Benjamini, Y., and Hochberg, Y. (1995). Controlling the False Discovery Rate: A Practical and Powerful Approach to Multiple Testing. Journal of the Royal Statistical Society, Series B (Methodological) *57*, 12.
Brennecke, P., Anders, S., Kim, J.K., Kolodziejczyk, A.A., Zhang, X., Proserpio, V., Baying, B., Benes, V., Teichmann, S.A., Marioni, J.C.*, et al.* (2013). Accounting for technical noise in single-cell RNA-seq experiments. Nat Methods *10*, 1093-1095.
Dobin, A., Davis, C.A., Schlesinger, F., Drenkow, J., Zaleski, C., Jha, S., Batut, P., Chaisson, M., and Gingeras, T.R. (2013). STAR: ultrafast universal RNA-seq aligner. Bioinformatics *29*, 15-21.
Feitelson, D. (2003). Comparing Partitions with Spie Charts. Technical Report 2003-87 *School of Computer Science and Engigeering, The Hebrew University of Jerusalem*.
Giorgetti, A., Castano, J., Bueno, C., Diaz de la Guardia, R., Delgado, M., Bigas, A., Espinosa, L., and Menendez, P. (2017). Proinflammatory signals are insufficient to drive definitive hematopoietic specification of human HSCs in vitro. Exp Hematol *45*, 85-93 e82.
Giorgetti, A., Montserrat, N., Aasen, T., Gonzalez, F., Rodriguez-Piza, I., Vassena, R., Raya, A., Boue, S., Barrero, M.J., Corbella, B.A.*, et al.* (2009). Generation of induced pluripotent stem cells from human cord blood using OCT4 and SOX2. Cell Stem Cell *5*, 353-357.
Im, H., Grass, J.A., Johnson, K.D., Kim, S.I., Boyer, M.E., Imbalzano, A.N., Bieker, J.J., and Bresnick, E.H. (2005). Chromatin domain activation via GATA-1 utilization of a small subset of dispersed GATA motifs within a broad chromosomal region. Proc Natl Acad Sci U S A *102*, 17065-17070.
Krijthe, J.H. (2015). Rtsne: T-Distributed Stochastic Neighbor Embedding using a Barnes-Hut Implementation.
Kuleshov, M.V., Jones, M.R., Rouillard, A.D., Fernandez, N.F., Duan, Q., Wang, Z., Koplev, S., Jenkins, S.L., Jagodnik, K.M., Lachmann, A.*, et al.* (2016). Enrichr: a comprehensive gene set enrichment analysis web server 2016 update. Nucleic Acids Res *44*, W90-97.

Langmead, B., Trapnell, C., Pop, M., and Salzberg, S.L. (2009). Ultrafast and memory-efficient alignment of short DNA sequences to the human genome. Genome Biol *10*, R25.
Love, M.I., Huber, W., and Anders, S. (2014). Moderated estimation of fold change and dispersion for RNA-seq data with DESeq2. Genome Biol *15*, 550.
Machanick, P., and Bailey, T.L. (2011). MEME-ChIP: motif analysis of large DNA datasets. Bioinformatics *27*, 1696-1697.
Peres, J., Kwesi-Maliepaard, E.M., Rambow, F., Larue, L., and Prince, S. (2017). The tumour suppressor, miR-137, inhibits malignant melanoma migration by targetting the TBX3 transcription factor. Cancer Lett *405*, 111-119.
Picelli, S., Faridani, O.R., Bjorklund, A.K., Winberg, G., Sagasser, S., and Sandberg, R. (2014). Full-length RNA-seq from single cells using Smart-seq2. Nat Protoc *9*, 171-181.
Ramos-Mejia, V., Navarro-Montero, O., Ayllon, V., Bueno, C., Romero, T., Real, P.J., and Menendez, P. (2014). HOXA9 promotes hematopoietic commitment of human embryonic stem cells. Blood *124*, 3065-3075.
Ran, F.A., Hsu, P.D., Wright, J., Agarwala, V., Scott, D.A., and Zhang, F. (2013). Genome engineering using the CRISPR-Cas9 system. Nat Protoc *8*, 2281-2308.
Sturgeon, C.M., Ditadi, A., Awong, G., Kennedy, M., and Keller, G. (2014). Wnt signaling controls the specification of definitive and primitive hematopoiesis from human pluripotent stem cells. Nat Biotechnol *32*, 554-561.
Tyner, C., Barber, G.P., Casper, J., Clawson, H., Diekhans, M., Eisenhart, C., Fischer, C.M., Gibson, D., Gonzalez, J.N., Guruvadoo, L.*, et al.* (2017). The UCSC Genome Browser database: 2017 update. Nucleic Acids Res *45*, D626-D634.
Venables, W.N., and Ripley, B.D. (2002). Modern Applied Statistics with S (Springer-Verlag New York).
Zhang, Y., Liu, T., Meyer, C.A., Eeckhoute, J., Johnson, D.S., Bernstein, B.E., Nusbaum, C., Myers, R.M., Brown, M., Li, W.*, et al.* (2008). Model-based analysis of ChIP-Seq (MACS). Genome Biol *9*, R137.
